# α-Glucosidase Inhibitors From the Coral-Associated Fungus *Aspergillus terreus*

**DOI:** 10.3389/fchem.2018.00422

**Published:** 2018-09-13

**Authors:** Mengting Liu, Changxing Qi, Weiguang Sun, Ling Shen, Jianping Wang, Junjun Liu, Yongji Lai, Yongbo Xue, Zhengxi Hu, Yonghui Zhang

**Affiliations:** ^1^Hubei Key Laboratory of Natural Medicinal Chemistry and Resource Evaluation, School of Pharmacy, Tongji Medical College, Huazhong University of Science and Technology, Wuhan, China; ^2^Department of Pharmacy, the Central Hospital of Wuhan, Wuhan, China

**Keywords:** coral-associated fungus, *Aspergillus terreus*, butenolide derivatives, structure reassignments, α-glucosidase inhibitors

## Abstract

Nine novel butenolide derivatives, including four pairs of enantiomers, named (±)-asperteretones A–D (**1a/1b–4a/4b**), and a racemate, named asperteretone E (**5**), were isolated and identified from the coral**-**associated fungus *Aspergillus terreus*. All the structures were established based on extensive spectroscopic analyses, including HRESIMS and NMR data. The chiral chromatography analyses allowed the separation of (±)-asperteretones A–D, whose absolute configurations were further confirmed by experimental and calculated electronic circular dichroism (ECD) analysis. Structurally, compounds **2**–**5** represented the first examples of prenylated γ-butenolides bearing 2-phenyl-3-benzyl-4*H*-furan-1-one motifs, and their crucial biogenetically related metabolite, compound **1**, was uniquely defined by an unexpected cleavage of oxygen bridge between C-1 and C-4. Importantly, (±)-asperteretal D and (4*S*)-4-decarboxylflavipesolide C were revised to (±)-asperteretones B (**2a**/**2b**) and D (**4**), respectively. Additionally, compounds **1a/1b–4a/4b** and **5** were evaluated for the α-glucosidase inhibitory activity, and all these compounds exhibited potent inhibitory potency against α-glucosidase, with IC_50_ values ranging from 15.7 ± 1.1 to 53.1 ± 1.4 μM, which was much lower than that of the positive control acarbose (IC_50_ = 154.7 ± 8.1 μM), endowing them as promising leading molecules for the discovery of new α-glucosidase inhibitors for type-2 diabetes mellitus treatment.

## Introduction

Diabetes mellitus (DM) is one of the most serious chronic diseases with the ever-increasing incidence rates of obesity and aging of the general population throughout the world (Kopelman, 2000) In 2013, it was estimated that over 382 million people all over the world have DM and this number is predicted to increase up to 500 million in 2030, when this disease will be excepted to be the 7th leading cause of death (Lauritano and Ianora, 2016). Globally, type-2 diabetes (non-insulin-dependent DM) covered 90–95% of all the diabetes cases (Lauritano and Ianora, 2016). Postprandial hyperglycemia is an important factor for the induction of type-2 diabetes and complications related to the diseases, such as micro- and macro-vascular diseases (Baron, 1998). A good strategy to maintain the normal level of postprandial plasma glucose is to medicate in combination with dietary restriction and an exercise plan (Kim et al., 2008). In type-2 diabetes, delaying glucose absorption after meals by inhibition of α-glucosidase is known to help the therapy (Kim et al., 2008). For diabetic patients, α-glucosidase inhibitors (AGIs) are widely applied either as monotherapy or in combination with other oral hypoglycemic agents or insulin (Hung et al., 2012). However, AGIs-induced serious liver injuries and gastrointestinal side effects restricted the clinical practice (Yin et al., 2014; Kao et al., 2016). In view of the limited number of safe anti-diabetic drugs with low toxicity and ever-increasing number of diabetic patients, the exploration for new α-glucosidase inhibitors, attracted, and still attract great interests from scientific community.

**Figure 1:**
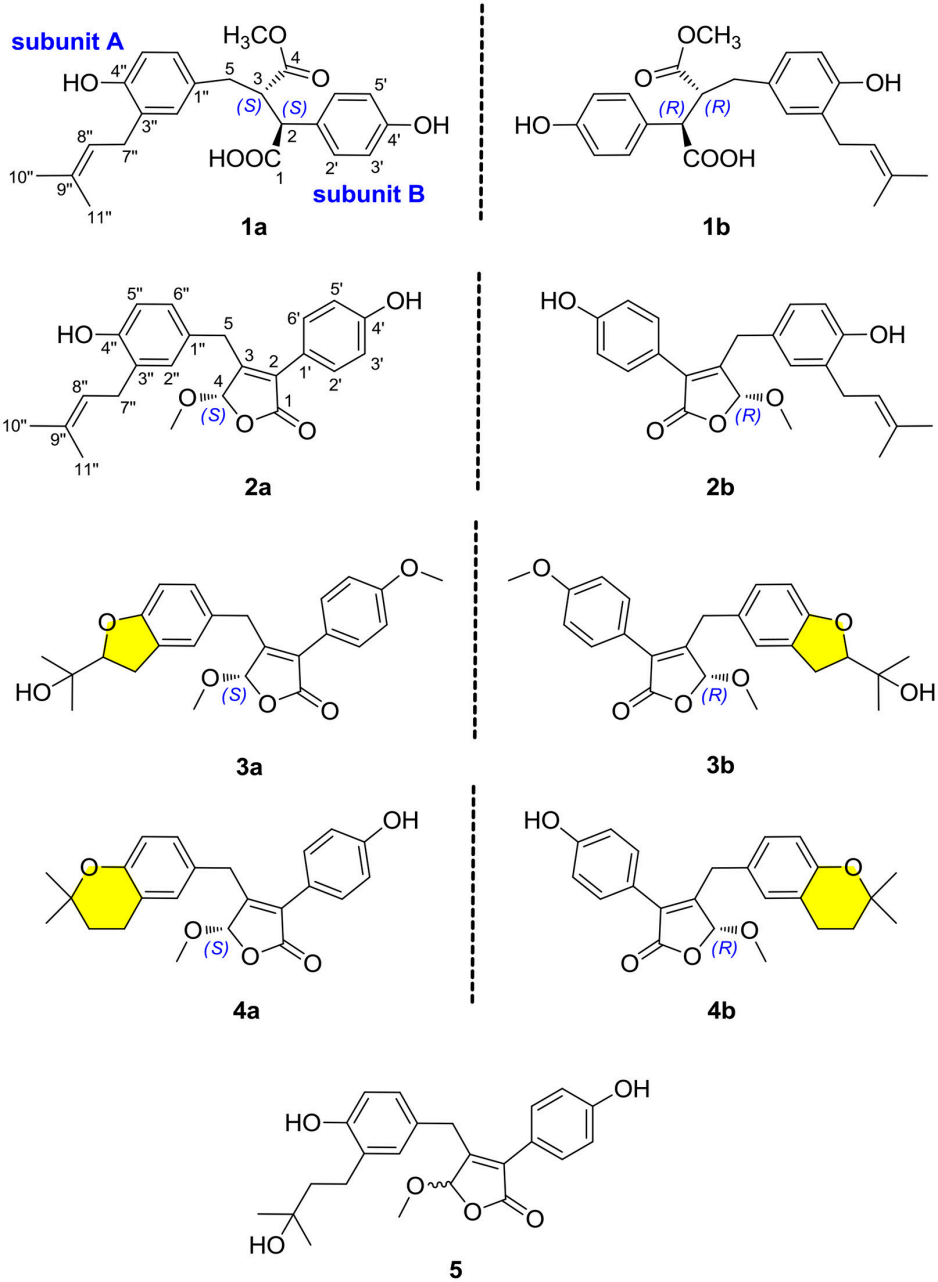
Chemical structures of compounds **1**–**5**.

In our continuous search for chemically novel and bioactive secondary metabolites from marine fungi (Hu et al., 2014; Liu et al., 2018a,b; Yang et al., 2018), we focused our attention on a coral-associated fungus *Aspergillus terreus*. A systematic chemical investigation on the ethyl acetate extracts of rice medium of this fungal strain facilitated the isolation and identification of nine novel butenolide derivatives, including four pairs of enantiomers, named (±)-asperteretones A–D **(1a/1b–4a/4b)**, and a racemate, named asperteretone E **(5)**. Structurally, compounds **2**–**5** represented the first examples of prenylated γ-butenolides bearing 2-phenyl-3-benzyl-4*H*-furan-1-one motifs, and their crucial biogenetically related metabolite, compound **1**, was uniquely defined by an unexpected cleavage of oxygen bridge between C-1 and C-4. Importantly, by an experimental validation method, (±)-asperteretal D and (4*S*)-4-decarboxylflavipesolide C were revised to (±)-asperteretones B **(2a/2b)** and D **(4)**, respectively. Herein, the detailed isolation, structure elucidation, and α-glucosidase inhibitory activity of these compounds (Figure 1) are described.

## Materials and methods

### General experimental procedures

Optical rotations and IR data were collected from a PerkinElmer PE-341 instrument (PerkinElmer, Waltham, MA, USA) and a Bruker Vertex 70 FT-IR spectrophotometer (Bruker, Karlsruhe, Germany) with KBr pellets, respectively. UV and ECD spectra were collected from a Varian Cary 50 UV/vis spectrophotometer (Varian, Salt Lake City, UT, USA) and a JASCO-810 spectrometer, respectively. 1D and 2D NMR spectra were measured by using a Bruker AM-400 NMR spectrometer (Bruker, Karlsruhe, Germany). The solvent or solvent impurity peaks for methanol-*d*_4_ (δ_H_ 3.31 and δ_C_ 49.0) and CDCl_3_ (δ_H_ 7.24 and δ_C_ 77.23) were referenced to the ^1^H and ^13^C NMR chemical shifts. High-resolution electrospray ionization mass spectrometry (HRESIMS) were measured by using a Thermo Fisher LTQ XL LC/MS (Thermo Fisher, Palo Alto, CA, USA). Column chromatography (CC) was carried out by using silica gel (200–300 mesh; Qingdao Marine Chemical Inc., China), Sephadex LH-20 (40–70 μm, Amersham Pharmacia Biotech AB, Uppsala, Sweden, Sweden), and octadecylsilyl (ODS, 50 μm, YMC Co. Ltd., Japan). Semi-preparative HPLC separations were carried out on an Agilent 1100 liquid chromatograph with a Zorbax SB-C_18_ (9.4 × 250 mm) and a Daicel IC column (5 μm, 4.6 × 250 mm, Daicel Chiral Technologies Co., Ltd., China) column. Thin-layer chromatography (TLC) was carried out by using silica gel 60 F_254_ (Yantai Chemical Industry Research Institute) and RP-C_18_ F_254_ plates (Merck, Germany).

### Fungus material

The fungus *Aspergillus terreus* was isolated from the soft coral *Sarcophyton subviride*, which was collected from the Xisha Island in the South China Sea. This strain was cultivated on potato dextrose agar (PDA) medium and identified by one of the authors (JW), based on its morphological properties and ITS sequence analysis (GenBank access no. MF972904). The fungal strain was reserved in the culture collection of Tongji Medical College, Huazhong University of Science and Technology.

### Cultivation, extraction, and isolation

The strain *Aspergillus terreus* was cultivated on PDA (Potato Dextrose Agar) medium at 28°C for 1 week to prepare the seed cultures. Agar plugs were cut into small pieces (approximately 0.5 × 0.5 × 0.5 cm^3^) and then was inoculated in 300 × 500 mL Erlenmeyer flasks which were previously sterilized by autoclaving, each containing 200 g rice and 200 mL distilled water. All flasks were incubated at 28°C for 28 days. Then, the whole rice solid medium was extracted seven times in 95% aqueous EtOH at room temperature, and the solvent was removed under reduced pressure to afford a crude extract, which was partitioned with ethyl acetate against water to obtain the ethyl acetate soluble part (1.5 kg). The organic extract was separated by silica gel CC (100–200 mesh) with a stepwise gradient elution of petroleum ether–ethyl acetate–MeOH (10:1:0, 7:1:0, 5:1:0, 3:1:0, 1:1:0, 2:2:1, 1:1:1) to afford seven fractions (A–G).

Fraction C (75 g) was separated by an RP-C_18_ column using MeOH–H_2_O (from 20:80 to 100:0, v/v) to give five main fractions (C1–C5). Fraction C3 (2.3 g) was further purified by Sephadex LH-20 using CH_2_Cl_2_-MeOH (1:1, v/v) to yield two fractions (C3.1–C3.2). Fraction C3.2 was applied to silica gel CC eluted with petroleum ether–ethyl acetate (stepwise 4:1–1:1) to furnish four additional fractions (C3.2.1–C3.2.4). Repeated purification of fraction C3.2.3 (92 mg) by semi-preparative HPLC with CH_3_CN–H_2_O (50:50, v/v, 3.0 mL/min) to yield compounds **2** (4.8 mg; *t*_R_ 12.5 min) and **4** (4.0 mg; *t*_R_ 28.2 min). Afterwards, compound **2** was further purified by chiral HPLC using a Daicel IC column (isopropanol–*n*-hexane, 8:92, v/v, 2.0 mL/min) to give **2a** (2.2 mg; *t*_R_ 60.7 min) and **2b** (2.3 mg; *t*_R_ 55.2 min). The enantiomers **4a** (1.8 mg; *t*_R_ 50.5 min) and **4b** (2.0 mg; *t*_R_ 41.8 min) were obtained by chiral HPLC separation of compound **4** using a Daicel IC column eluted with isopropanol–*n*-hexane (8:92, v/v, 2.0 mL/min).

Fraction D (198 g) was subjected to an RP-C_18_ column eluted with MeOH–H_2_O (from 20:80 to 100:0, v/v) to yield five fractions (D1–D5). Fraction D4 (2.4 g) was applied to Sephadex LH-20 eluted with CH_2_Cl_2_-MeOH (1:1, v/v) and further purified by semi-preparative HPLC using CH_3_CN–H_2_O (60:40, v/v, 3.0 mL/min) to yield a racemic mixture **3** (9.6 mg; *t*_R_ 38.2 min). The enantiomers **3a** (1.1 mg; *t*_R_ 12.5 min) and **3b** (1.8 mg; *t*_R_ 14.8 min) were further purified by chiral HPLC using a Daicel IC column eluted with isopropanol–*n*-hexane (8:92, v/v, 2.0 mL/min).

Fraction E (186 g) was chromatographed on silica gel CC (CH_2_Cl_2_-MeOH, 1:0–50:1, v/v) to yield five main fractions (E1–E5). Repeated purification of fraction E5 (1.3 g) using Sephadex LH-20 with CH_3_OH as eluent and RP-C_18_ column (MeOH–H_2_O, from 30:70 to 100:0, v/v) to give three mixtures (E5.1–E5.3). Fractions E5.1 (210 mg) was subjected to semi-preparative HPLC (MeOH–H_2_O, 60:40, v/v, 3.0 mL/min) to afford compound **1** (6.7 mg; *t*_R_ 43 min). Subsequently, compound **1** was subjected to chiral HPLC using a Daicel IC column (isopropanol–*n*-hexane, 7:93, v/v, 2.0 mL/min), resulting in the separation of **1a** (2.7 mg; *t*_R_ 12.4 min) and **1b** (2.5 mg; *t*_R_ 16.2 min). A racemic mixture **5** (3.2 mg) was isolated by semi-preparative HPLC (MeOH–H_2_O, 70:30, v/v, 3.0 mL/min) from fraction E5.2 (54.5 mg).

1. (±)-Asperteretone A (**1**). White, amorphous powder; [α]${}_{\text{D}}^{25}$ 0 (*c* 0.1, MeOH); UV (MeOH) λ_max_ (log ε) = 202 (4.63), 229 (4.06), 279 (3.43) nm; IR ν_max_ = 3435, 2925, 1718, 1623, 1511, 1446, 1381, 1257, 1179, 1047, 829, 573 cm^−1^; HRESIMS *m/z* 421.1622 [M + Na]^+^ (calcd for C_23_H_26_O_6_Na, 421.1627); For ^1^H NMR and ^13^C NMR data, see Table 1.
2. (−)-Asperteretone A (**1a**). White, amorphous powder; [α]${}_{\text{D}}^{25}$ −34 (*c* 0.1, MeOH); ECD (*c* 0.1, MeOH) = Δε_210_ −0.85, Δε_227_ −1.52.
3. (+)-Asperteretone A (**1b**). White, amorphous powder; [α]${}_{\text{D}}^{25}$ +37 (*c* 0.1, MeOH); ECD (*c* 0.1, MeOH) = Δε_209_ +6.18, Δε_226_ +5.80.
4. (±)-Asperteretone B (**2**). White, amorphous powder; [α]${}_{\text{D}}^{25}$ 0 (*c* 0.1, MeOH); UV (MeOH) λ_max_ (log ε) = 202 (4.21), 215 (3.85), 285 (3.50) nm; IR ν_max_ = 3425, 2927, 2853, 1747, 1611, 1514, 1440, 1372, 1342, 1269, 1204, 1144, 1113, 1020, 987, 946, 839, 789, 557, 534 cm^−1^; HRESIMS *m/z* 403.1526 [M + Na]^+^ (calcd for C_23_H_24_O_5_Na, 403.1521); For ^1^H NMR and ^13^C NMR data, see Table 2.
5. (−)-Asperteretone B (**2a**). White, amorphous powder; [α]${}_{\text{D}}^{25}$ −140 (*c* 0.1, MeOH); ECD (*c* 0.1, MeOH) = Δε_207_−16.99, Δε_232_−2.90, Δε_282_−10.57.
6. (+)-Asperteretone B (**2b**). White, amorphous powder; [α]${}_{\text{D}}^{25}$ +136 (*c* 0.1, MeOH); ECD (*c* 0.1, MeOH) = Δε_207_ +10.92, Δε_232_ +3.56, Δε_282_ +8.98.
7. (±)-Asperteretone C (**3**). White, amorphous powder; [α]${}_{\text{D}}^{25}$ 0 (*c* 0.1, MeOH); UV (MeOH) λ_max_ (log ε) = 202 (4.23), 223 (3.77), 285 (3.67) nm; IR ν_max_ = 3483, 2991, 2952, 2878, 1712, 1637, 1453, 1434, 1396, 1351, 1267, 1170, 1133, 1008, 988, 967, 942, 900, 838, 736, 702, 605, 564 cm^−1^; HRESIMS *m/z* 411.1802 [M + H]^+^ (calcd for C_24_H_27_O_6_, 411.1808) and *m/z* 433.1635 [M + Na]^+^ (calcd for C_24_H_26_O_6_Na, 433.1627); For ^1^H NMR and ^13^C NMR data, see Table 1.
8. (−)-Asperteretone C (**3a**). White, amorphous powder; [α]${}_{\text{D}}^{25}$ −135 (*c* 0.1, MeOH); ECD (*c* 0.1, MeOH) = Δε_207_ −9.13, Δε_232_ −3.29, Δε_282_ −5.67.
9. (+)-Asperteretone C (**3b**). White, amorphous powder; [α]${}_{\text{D}}^{25}$ +137 (*c* 0.1, MeOH); ECD (*c* 0.1, MeOH) = Δε_208_ +9.79, Δε_236_ +1.78, Δε_284_ +8.67.
10. (±)-Asperteretone D (**4**). White, amorphous powder; [α]${}_{\text{D}}^{25}$ 0 (*c* 0.1, MeOH); UV (MeOH) λ_max_ (log ε) = 202 (4.62), 221 (4.32), 283 (4.05) nm; IR ν_max_ = 3432, 2974, 2930, 2853, 1756, 1616, 1503, 1444, 1374, 1263, 1208, 1158, 1116, 1028, 994, 837, 670 cm^−1^; HRESIMS *m/z* 381.1692 [M + H]^+^ (calcd for C_23_H_25_O_5_, 381.1702) and *m/z* 403.1513 [M + Na]^+^ (calcd for C_23_H_24_O_5_Na, 403.1521); For ^1^H NMR and ^13^C NMR data, see Table 1.
11. (−)-Asperteretone D (**4a**). White, amorphous powder; [α]${}_{\text{D}}^{25}$ −120 (*c* 0.1, CH_2_Cl_2_); ECD (*c* 0.1, MeOH) = Δε_207_−13.10, Δε_232_−3.81, Δε_283_−7.07.
12. (+)-Asperteretone D (**4b**). White, amorphous powder; [α]${}_{\text{D}}^{25}$ +116 (*c* 0.1, CH_2_Cl_2_); ECD (*c* 0.1, MeOH) = Δε_207_ +9.95, Δε_232_ +4.60, Δε_282_ +9.14.
13. Asperteretone E (**5**). White, amorphous powder; [α]${}_{\text{D}}^{25}$ 0 (*c* 0.1, MeOH); UV (MeOH) λ_max_ (log ε) = 202 (4.55), 218 (4.29), 285 (4.06) nm; IR ν_max_ = 3428, 2965, 2926, 2853, 1750, 1615, 1512, 1441, 1373, 1266, 1207, 1109, 1032, 946, 837, 565 cm^−1^; HRESIMS *m/z* 421.1626 [M + Na]^+^ (calcd for C_23_H_26_O_6_Na, 421.1627); For ^1^H NMR and ^13^C NMR data, see Table 1.

**Table 1:** ^1^H and ^13^C NMR data for compounds **1** and **3–5** in methanol-*d*_4_ (δ in ppm and *J* in Hz).

**No**.	**1**		**3**		**4**		**5**	
	**δ${}_{{\text{H}}^{a,b}}$**	**δ_C_*^*c*^***	**δ${}_{{\text{H}}^{a,b}}$**	**δ_C_*^*c*^***	**δ${}_{{\text{H}}^{a,b}}$**	**δ_C_*^*c*^***	**δ${}_{{\text{H}}^{a,b}}$**	**δ_C_*^*c*^***
1	–	178.3 C	–	172.7 C	–	172.9 C	–	173.0 C
2	3.61 d (11.5)	55.3 CH	–	130.1 C	–	130.2 C	–	130.0 C
3	3.26 m	52.2 CH	–	158.4 C	–	157.8 C	–	158.1 C
4	–	177.4 C	5.57 s	104.2 CH	5.56 s	104.1 CH	5.55 s	104.2 CH
5	2.38 dd (8.9, 13.7); 2.58 dd (4.0, 13.7)	36.5 CH_2_	3.64 d (15.4); 3.96 d (15.4)	32.9 CH_2_	3.60 d (15.3); 3.93 d (15.3)	32.7 CH_2_	3.60 d (15.3); 3.94 d (15.3)	32.8 CH_2_
4-OMe	3.52 s	51.8 CH_3_	3.52 s	57.4 CH_3_	3.52 s	57.3 CH_3_	3.53 s	57.4 CH_3_
1'	–	130.4 C	–	122.7 C	–	121.5 C	–	121.5 C
2'	7.21 d (8.4)	130.9 CH	7.44 d (8.9)	131.6 CH	7.34 d (8.7)	131.6 CH	7.36 d (8.7)	131.6 CH
3'	6.79 d (8.4)	116.4 CH	6.99 d (8.9)	115.0 CH	6.85 d (8.7)	116.3 CH	6.86 d (8.7)	116.4 CH
4'	–	157.8 C	–	161.7 C	–	159.5 C	–	159.5 C
5'	6.79 d (8.4)	116.4 CH	6.99 d (8.9)	115.0 CH	6.85 d (8.7)	116.3 CH	6.86 d (8.7)	116.4 CH
6'	7.21 d (8.4)	130.9 CH	7.44 d (8.9)	131.6 CH	7.34 d (8.7)	131.6 CH	7.36 d (8.7)	131.6 CH
4'-OMe	–	–	3.82 s	55.8 CH_3_	–	–	–	–
1”	–	130.3 C	–	129.3 C	–	128.6 C	–	128.3 C
2”	6.60 d (2.0)	131.0 CH	6.95 s	126.4 CH	6.84 s	130.9 CH	6.87 d (2.3)	131.4 CH
3”	–	128.8 C	–	129.4 C	–	122.6 C	–	131.0 C
4”	–	154.5 C	–	160.4 C	–	154.3 C	–	155.3 C
5”	6.59 d (8.2)	115.5 CH	6.65 d (8.1)	110.0 CH	6.63 d (9.0)	118.5 CH	6.69 d (8.2)	116.2 CH
6”	6.55 dd (2.0, 8.2)	128.0 CH	6.87 d (8.1)	129.4 CH	6.85 d (9.0)	128.7 CH	6.81 dd (2.3, 8.2)	128.1 CH
7”	3.21 m	29.0 CH_2_	3.12 dd (4.2, 9.0)	31.5 CH_2_	2.72 t (6.8)	23.3 CH_2_	2.61 m	26.2 CH_2_
8”	5.27 m	124.1 CH	4.56 t (9.0)	90.6 CH	1.77 t (6.8)	33.7 CH_2_	1.68 m	44.9 CH_2_
9”	-	132.8 C	–	72.5 C	–	75.3 C	–	71.5 C
10”	1.74 s	26.0 CH_3_	1.23 s	25.2 CH_3_	1.28 s	27.0 CH_3_	1.24 s	29.1 CH_3_
11”	1.70 s	17.8 CH_3_	1.20 s	25.4 CH_3_	1.28 s	27.1 CH_3_	1.24 s	29.1 CH_3_

*^a^ Recorded at 400 MHz*.

*^b^ “m” means overlapped or multiplet with other signals*.

*^c^ Recorded at 100 MHz*.

**Table 2:** Comparison of the ^1^H and ^13^C NMR data for reported (±)-asperteretal D and **2** in CDCl_3_ (δ in ppm and *J* in Hz).

**No**.	**(**±**)-asperteretal D**		**2**	
	**δ${}_{{\text{H}}^{a,c}}$**	**δ_C_*^*d*^***	**δ_H_*^*b, c*^***	**δ_C_*^*e*^***
1	–	171.4 C	–	171.2 C
2	–	129.3 C	–	129.2 C
3	–	156.3 C	–	156.2 C
4	5.45 s	102.6 CH	5.44 s	102.5 CH
5	3.57 d (15.2); 3.96 d (15.2)	32.0 CH_2_	3.57 d (15.2); 3.96 d (15.2)	32.0 CH_2_
4-OMe	3.53 s	57.4 CH_3_	3.53 s	57.4 CH_3_
1'	–	121.6 C	–	121.8 C
2'	7.38 d (8.7)	130.8 CH	7.40 d (8.6)	130.8 CH
3'	6.87 d (8.7)	115.9 CH	6.88 d (8.6)	115.8 CH
4'	–	156.7 C	–	156.6 C
5'	6.87 d (8.7)	115.9 CH	6.88 d (8.6)	115.8 CH
6'	7.38 d (8.7)	130.8 CH	7.40 d (8.6)	130.8 CH
1”	–	128.3 C	–	128.4 C
2”	6.85 m	130.5 CH	6.86 s	130.5 CH
3”	–	127.8 C	–	127.7 C
4”	–	153.6 C	–	153.6 C
5”	6.72 d (8.6)	116.4 CH	6.73 d (7.9)	116.4 CH
6”	6.86 m	127.9 CH	6.87 d (7.9)	127.9 CH
7”	3.30 d (7.1)	29.9 CH_2_	3.30 d (7.2)	29.9 CH_2_
8”	5.26 t (7.1)	121.6 CH	5.26 t (7.2)	121.6 CH
9”	–	135.3 C	–	135.4 C
10”	1.75 s	26.0 CH_3_	1.75 s	26.0 CH_3_
11”	1.74 s	18.1 CH_3_	1.75 s	18.1 CH_3_

*^a^ Recorded at 600 MHz*.

*^b^ Recorded at 400 MHz*.

*^c^ “m” means overlapped or multiplet with other signals*.

*^d^ Recorded at 150 MHz*.

*^e^ Recorded at 100 MHz*.

### ECD calculations

The theoretical calculations of compounds **1a**/**1b** and **2a**/**2b** were performed using Gaussian 09 and figured using GaussView 5.0 (He et al., 2017a,b,c; Hu et al., 2017). Conformation search using molecular mechanics calculations was performed in the Discovery Studio 3.5 Client with MMFF force field with 20 kcal mol^−1^ upper energy limit (Smith and Goodman, 2010). The optimized conformation geometries and thermodynamic parameters of all selected conformations were provided. The predominant conformers were optimized at B3LYP/6-31G(d,p) level. The theoretical calculation of ECD was performed using time dependent Density Functional Theory (TDDFT) at the B3LYP/6-31G(d,p) level in MeOH with PCM model (Miertus et al., 1981). The ECD spectra of compounds **1a**/**1b** and **2a**/**2b** were obtained by weighing the Boltzmann distribution rate of each geometric conformation (Tähtinen et al., 2003).

The ECD spectra were simulated by overlapping Gaussian functions for each transition according to:

$$\begin{array}{l} \Delta \varepsilon \left(E\right)=\frac{1}{2.297\times 10^{-39}}\times \frac{1}{\sqrt{2\pi \sigma }}\overset{A}{\underset{i}{\sum }}\Delta E_{i}R_{i}e^{-{\left[\left(E-E_{i}\right)/\left(2\sigma \right)\right]}^{2}} \end{array}$$

The σ represented the width of the band at 1/*e* height, and Δ*E*_*i*_ and *R*_*i*_ were the excitation energies and rotational strengths for transition *i*, respectively. *R*_vel_ had been used in this work.

### α-glucosidase inhibitory assay

The α-glucosidase enzyme from *Saccharomyces cerevisiae* (Sigma Aldrich, USA) solution (1.5 U/mL) was prepared by dissolving the α-glucosidase in 200 M phosphate buffer (pH 6.8). The α-glucosidase enzyme solution (20 μL), test compounds (10 μL) and buffer (40 μL) were pipetted and mixed in a 96 well microtiter plate. The mixture was incubated at 37°C for 10 min. After incubation, *p*-nitrophenyl-α-D-glucopyranoside (PNP-G) substrate solution (10 μL, in 20 mM phosphate buffer) was added. The increment of absorbance due to the hydrolysis of PNP-G by α-glucosidase was measured at the wavelength of 410 nm with a microplate reader (Thermo Scientific, Waltham, MA). Acarbose was used as a positive control and averages of three replicates were calculated. The α-glucosidase inhibitory activity was expressed as percentage inhibition and was calculated using the following formula: inhibition (%) = [1**–**(OD_sample_/OD_blank_)] × 100. The half-maximal inhibitory concentration (IC_50_) was calculated as the compound concentration that is required for 50% inhibition, and the IC_50_ value of the acarbose was 154.7 ± 8.1 μM.

### Molecular docking simulation

The virtual docking was implemented in the Surflex-Dock module of the FlexX/Sybyl software, which is a fast docking method that allows sufficient flexibility of ligands and keeps the target protein rigid. Molecules were built with Chemdraw and optimized at molecular mechanical and semiempirical level by using Open Babel GUI. The crystallographic ligands were extracted from the active site and the designed ligands were modeled. All the hydrogen atoms were added to define the correct ionization and tautomeric states, and the carboxylate, phosphonate and sulphonate groups were considered in their charged form. In the docking calculation, the default FlexX scoring function was used for exhaustive searching, solid body optimizing and interaction scoring. Finally, the ligands with the lowest-energy and the most favorable orientation were selected.

## Results and discussion

(±)-Asperteretone A (**1a**/**1b**) were obtained as white, amorphous powders. The molecular formula C_23_H_26_O_6_ was deduced from the HRESIMS data at *m/z* 421.1622 [M + Na]^+^ (calcd for C_23_H_26_O_6_Na, 421.1627), requiring 11 degrees of unsaturation. Its IR spectrum displayed intense absorption bands for hydroxyl (3,435 cm^−1^), carbonyl (1,718 cm^−1^), and aromatic ring (1,623 and 1,511 cm^−1^). The ^1^H NMR spectrum (Table 1) of **1** showed characterized signals for a *para*-disubstituted phenyl group at δ_H_ 7.21 (d, *J* = 8.4 Hz, H-2', 6') and 6.79 (d, *J* = 8.4 Hz, H-3', 5'), a 1,3,4-trisubstituted phenyl group at δ_H_ 6.60 (d, *J* = 2.0 Hz, H-2”), 6.59 (d, *J* = 8.2 Hz, H-5”), and 6.55 (dd, *J* = 2.0, 8.2 Hz, H-6”), a prenyl group at δ_H_ 3.21 (m, H_2_-7”), 5.27 (m, H-8”), 1.74 (s, H_3_-10”), and 1.70 (s, H_3_-11”), a methoxy group at δ_H_ 3.52 (s, OMe-4), two methines at δ_H_ 3.61 (d, *J* = 11.5 Hz, H-2) and 3.26 (m, H-3), and a methylene at δ_H_ 2.38 (dd, *J* = 8.9, 13.7 Hz, H-5) and 2.58 (dd, *J* = 4.0, 13.7 Hz, H-5). The ^13^C NMR and DEPT spectra (Table 1) of **1** showed 23 carbon resonances categorized as two methyls, two methylenes, ten methines (including eight olefinic), eight nonprotonated carbons (including six olefinic, one carboxyl, and one ester carbonyl), and one methoxyl. The diagnostic data above indicated that compound **1** was a butenolide derivative.

**Figure 2:**
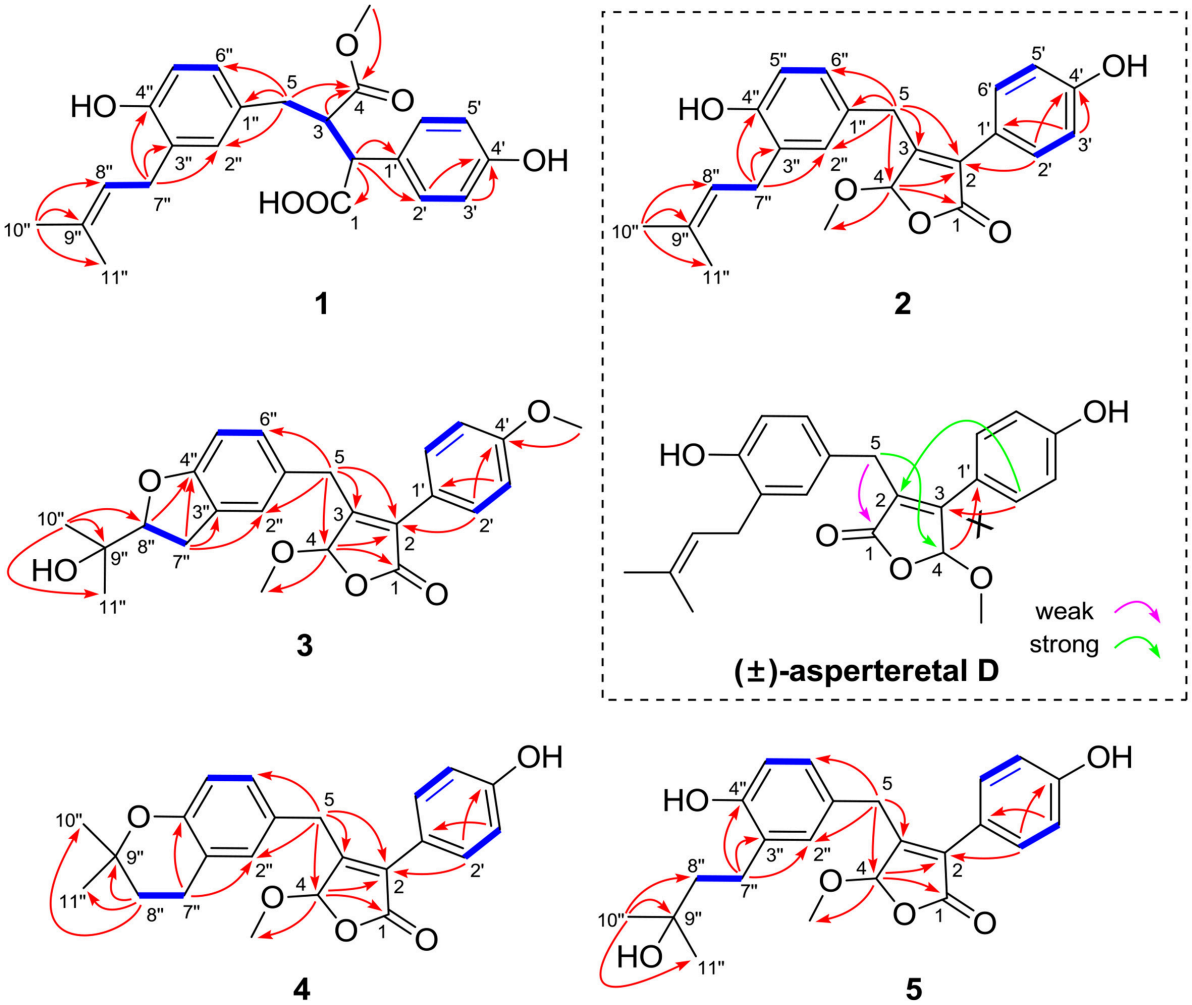
Selected ^1^H–^1^H COZY (blue bold lines) and HMBC (single arrows) correlations of compounds **1**–**5** and key HMBC analysis in original structure (±)-asperteretal D.

The protons and protonated carbon resonances in the NMR spectra were unambiguously assigned through the HSQC experiment. In the HMBC experiment (Figure 2), the correlations from H_3_-10” to C-8”, C-9”, and C-11” and from H_2_-7” to C-2”, C-3”, and C-4”, as well as the ^1^H–^1^H COZY correlations (Figure 2) of H_2_-7”/H-8” and H-5”/H-6”, confirmed the presence of the 1,3,4-trisubstituted phenyl group (subunit A) with a hydroxyl and a prenyl motif attached at C-4” and C-3”, respectively. Besides, the ^1^H–^1^H COZY correlations of H-2'/H-3' and H-5'/H-6' and HMBC correlations from both H-2' and H-3' to C-4' (δ_C_ 157.8) confirmed the presence of the *para*-disubstituted phenyl group (subunit B) with a hydroxyl motif attached at C-4'. Subunits A and B were connected through a “-CH_2_(5)-CH(3)-CH(2)-” group, as confirmed by the ^1^H–^1^H COZY correlations of H-2/H-3/H_2_-5 and HMBC correlations from H_2_-5 to C-1”, C-2”, and C-6” and from H-2 to C-1', C-2', and C-6'. In addition, the key HMBC correlations from H-3, H_2_-5, and OMe-4 to C-4 (δ_C_ 177.4) and H-2 to C-1 (δ_C_ 178.3) indicated that a methyl ester and a carboxyl group were attached at C-3 and C-2, respectively. Thus, the planar structure of **1** was determined.

**Figure 3:**
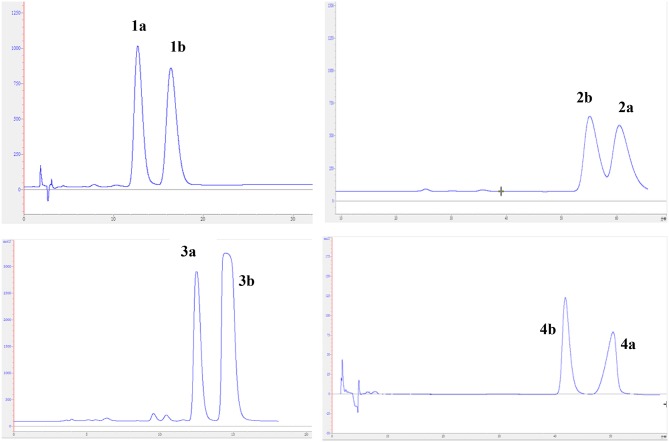
Chiral HPLC separation profiles of compounds **1a**/**1b**–**4a**/**4b**.

The experimental electronic circular dichroic (ECD) spectrum was measured in MeOH. Surprisingly, no apparent ECD Cotton effects were observed, suggesting that compound **1** was racemic, which adhered well to its lack of optical rotation. Subsequently, compound **1** was separated into two optically pure enantiomers, (−)-asperteretone A (**1a**, [α]${}_{\text{D}}^{25}$ −34) and (+)-asperteretone A (**1b**, [α]${}_{\text{D}}^{25}$ +37) using high performance liquid chromatography (HPLC) on a Daicel IC column (Figure 3).

**Figure 4:**
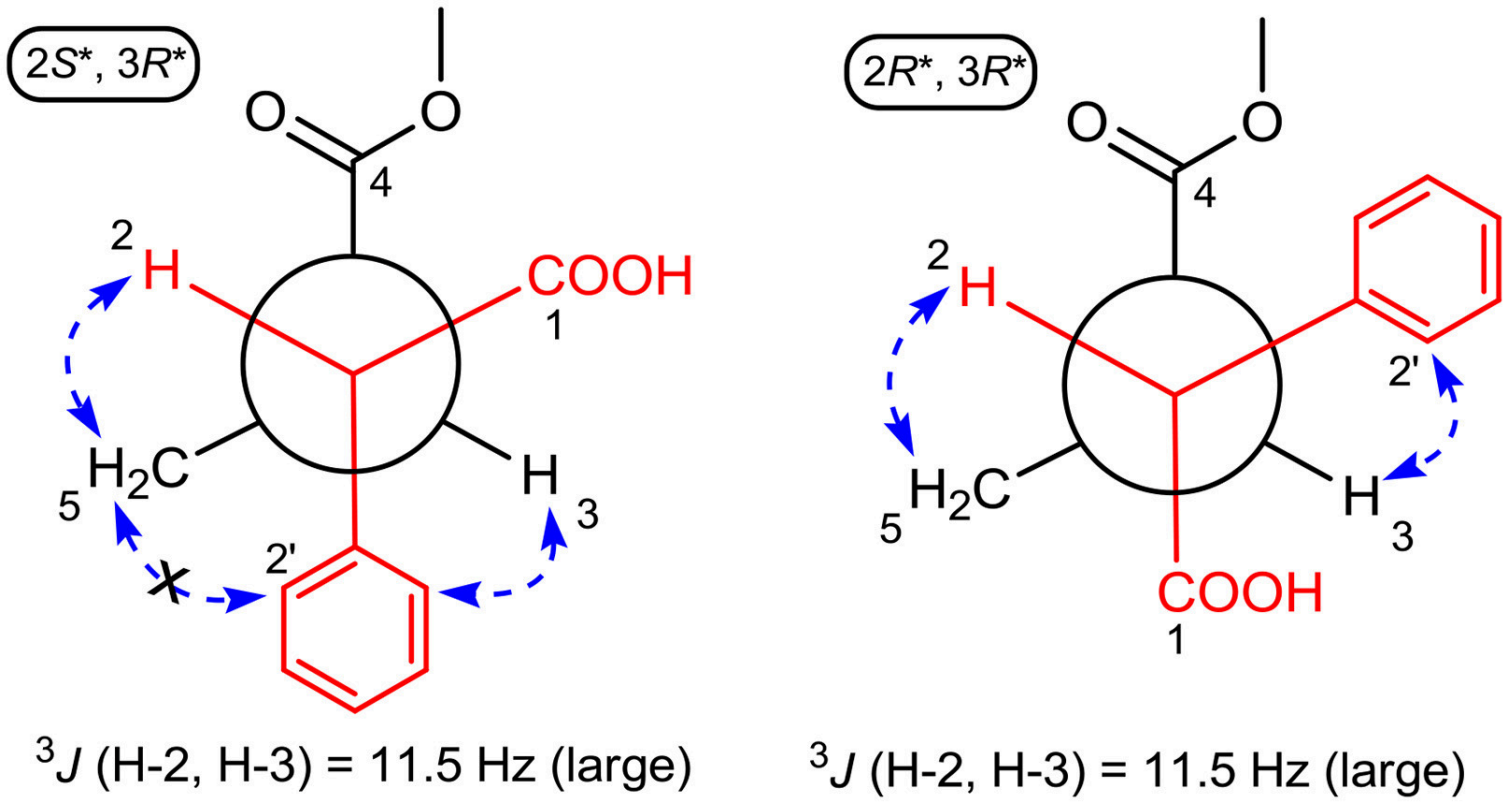
ROESY correlations (dashed blue arrows) and coupling constant were used to determine the relative configuration of **1** by optimized conformation analysis for C-2 to C-3.

The large coupling constant (*J* = 11.5 Hz) suggested the *trans*-relationship of H-2 and H-3, thus two possible optimal conformations existed for its conformation analysis (Figure 4). When the relative configuration of **1** was 2S^*^,3R^*^, despite there were obvious NOE correlations of H-2/H_2_-5 and H-3/H-6' (or H-2'), the diagnostic NOE correlation of H_2_-5/H-2' (or H-6') was not observed in the NOESY spectrum, indicating that the assumption for 2S^*^,3R^*^-configuration should be wrong. Thus, the relative configuration of **1** was deduced to be 2R^*^,3R^*^, which was completely supported by the NOE correlations of H-2/H_2_-5 and H-3/H-2' (or H-6'). Accordingly, the relative configuration of **1** was determined to be 2R^*^,3R^*^.

**Figure 5:**
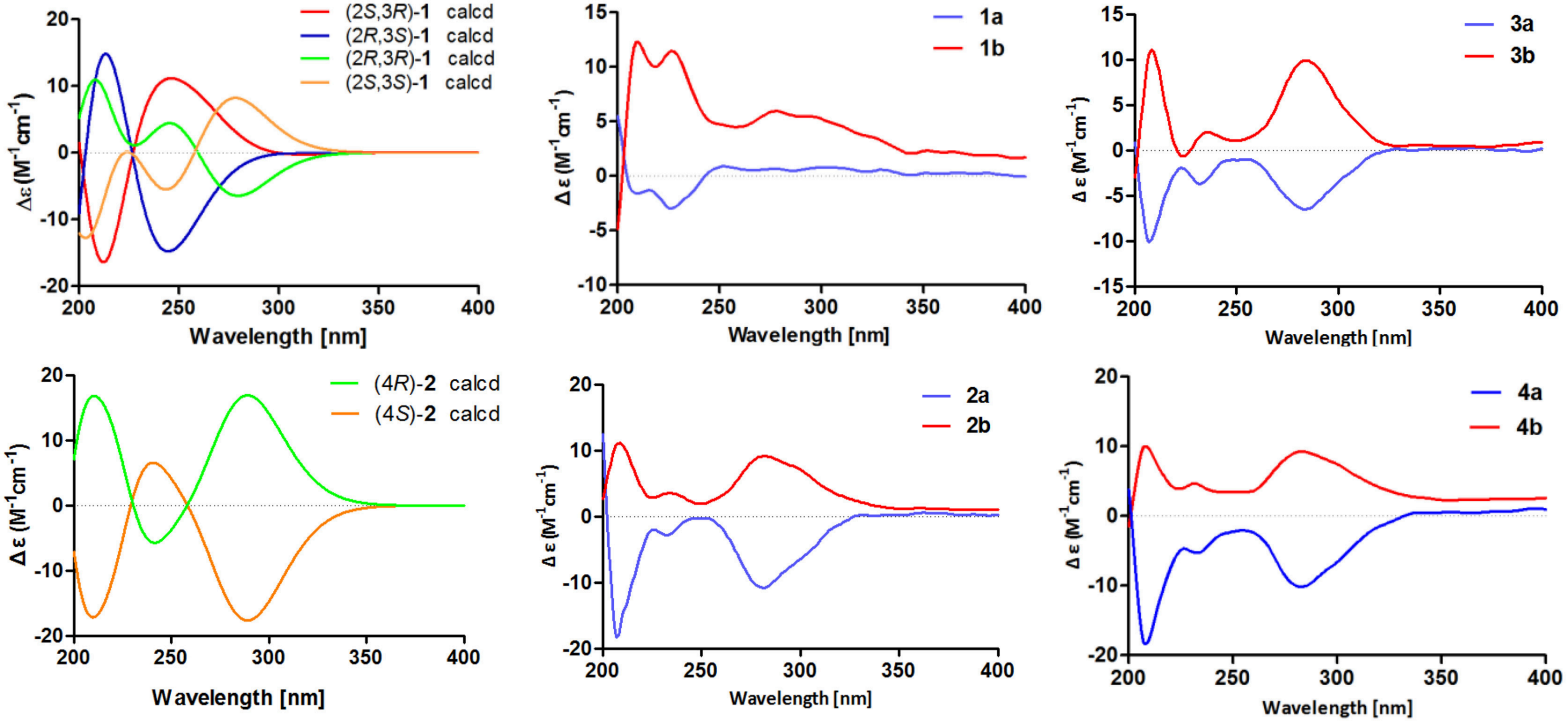
Calculated ECD spectra of (2*S*,3*R*)-**1**, (2*R*,3*S*)-**1**, (2*R*,3*R*)-**1**, (2*S*,3*S*)-**1**, (4*R*)-**2**, and (4*S*)-**2** and experimental ECD spectra of **1a**/**1b**–**4a**/**4b**.

To further confirm the above conclusion and determine the absolute configurations of **1a** and **1b**, a time-dependent density functional theory (TDDFT) method at the at B3LYP/6-31G(d,p) level in MeOH with PCM model was performed for (2*S*,3*R*)**-1**, (2*R*,3*S*)**-1**, (2*S*,3*S*)**-1**, and (2*R*,3*R*)**-1** (Figure 5), of which the DFT-calculated ECD spectra of (2*S*,3*S*)**-1** and (2*R*,3*R*)**-1** showed close similarities to the experimental ECD spectra of **1a** and **1b**, suggesting that the absolute structures of **1a** and **1b** should be assigned as (2*S*,3*S*)- and (2*R*,3*R*)-configuration, respectively.

(±)-Asperteretone B (**2a/2b**) were also obtained as white, amorphous powders and assigned the molecular formula C_23_H_24_O_5_, as determined from the HRESIMS analysis at *m/z* 403.1526 [M + Na]^+^ (calcd for C_23_H_24_O_5_Na, 403.1521) and ^13^C NMR data. The 1D (Table 2) and 2D NMR spectra of **2** were completely identical to that of the reported (±)-asperteretal D (Sun Y. et al., 2018), which drove us to believe that they shared the same structures. The 1,3,4-trisubstituted phenyl group with a C-4” hydroxyl and a C-3” prenyl motif and *para*-disubstituted phenyl group with a C-4' hydroxyl motif were explicitly confirmed by detailed analysis of the 2D NMR data (Figure 2) of **2**. However, a strong four-bond HMBC correlation from H_2_-5 to C-4 made us confused about the correctness of (±)-asperteretal D. After careful examination of the HMBC spectrum (Figure 2) of (±)-asperteretal D, key correlations from H-2' to C-3 and from H-4 to C-1' were not observed in the HMBC spectrum; on the contrary, two four-bond HMBC correlations from H-2' to C-2 and from H_2_-5 to C-4 were observed, which were also found in the HMBC spectrum of **2**. These data above suggested that (±)-asperteretal D should be structurally revised from 2-benzyl-3-phenyl-type to 2-phenyl-3-benzyl-type.

**Figure 6:**
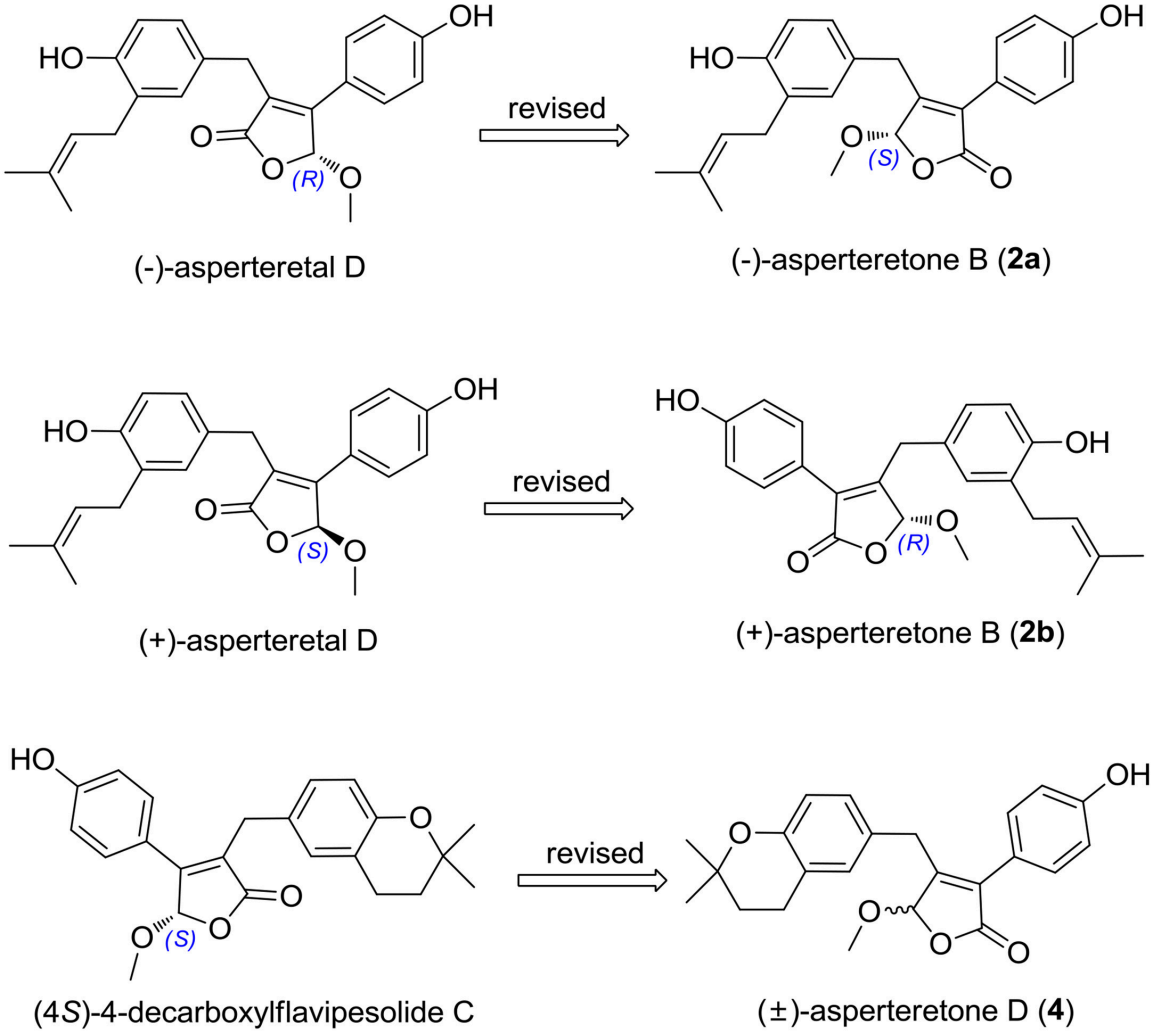
Structure reassignments of 2-benzyl-3-phenyl-type (±)-asperteretal D and (4*S*)-4-decarboxylflavipesolide C to 2-phenyl-3-benzyl-type (±)-asperteretones B (**2a**/**2b**) and D (**4**), respectively.

The chiral resolution (Figure 3) of **2** afforded a pair of enantiomers, (±)-asperteretone B (**2a/2b**). To determine their absolute configurations, the ECD spectra of (4*S*)**-2** and (4*R*)**-2** were calculated by the TDDFT methodology, which matched well with those of (−)-asperteretone B (**2a**) and (+)-asperteretone B (**2b**), respectively, indicating that the absolute stereochemistry of **2a** and **2b** should be 4*S*- and 4*R*-configuration, respectively. Furthermore, by comparison of their experimental ECD spectra (Figure 5) and similar specific rotation values, the structures of (−)-asperteretal D and (+)-asperteretal D were revised to **2a** and **2b** (Figure 6), respectively.

(±)-Asperteretone C (**3a/3b**), obtained as white, amorphous powders, were determined to have the molecular formula C_24_H_26_O_6_, as deduced from the HRESIMS data at *m/z* 411.1802 [M + H]^+^ (calcd for C_24_H_27_O_6_, 411.1808) and *m/z* 433.1635 [M + Na]^+^ (calcd for C_24_H_26_O_6_Na, 433.1627), which was indicative of twelve degrees of unsaturation. Its high similarities of NMR data (Table 1) with those of **2** suggested that **3** was also a butenolide derivative, with the differences that the *para*-disubstituted phenyl group was attached with a C-4' methoxy motif in **3** rather than a C-4' hydroxyl motif in **2**, and the 1,3,4-trisubstituted phenyl group attached with a C-4” hydroxyl and a C-3” prenyl motif in **2** was replaced by a 2-(2,3-dihydrobenzofuran-2-yl)propan-2-ol motif in **3**, as supported by the 2D NMR spectra (Figure 2), including HMBC and ^1^H–^1^H COZY correlations. Thus, the structure of **3** was determined.

Considering the similar structural features of **2** and **3**, we deduced that compound **3** was likely a racemic mixture. As expected, by chiral HPLC resolution (Figure 3), two isolates were obtained. Since no apparent Cotton effects were decisive for the absolute stereochemistry of C-8”, the experimental ECD spectra (Figure 5) of **3a** and **3b** were closely similar to those of (−)-asperteretone B (**2a**) and (+)-asperteretone B (**2b**), respectively, indicating that compounds **3a** and **3b** possessed the 4*S*- and 4*R*-configuration, respectively. Regrettably, the configuration of C-8” was difficult to be determined (Liu et al., 2018b).

(±)-Asperteretone D (**4a/4b**) gave the molecular formula C_23_H_24_O_5_, as determined by the HRESIMS analysis at *m/z* 381.1692 [M + H]^+^ (calcd for C_23_H_25_O_5_, 381.1702) and *m/z* 403.1513 [M + Na]^+^ (calcd for C_23_H_24_O_5_Na, 403.1521), corresponding to twelve degrees of unsaturation. Detailed analysis of the ^1^H and ^13^C NMR data (Tables 1, 2) of **4** and **2** suggested that they shared the similar structural features, differing in that the 1,3,4-trisubstituted phenyl group attached with a C-4” hydroxyl and a C-3” prenyl motif in **2** was replaced by the 1,3,4-trisubstituted phenyl with the fusion of gem-dimethyl substituted tetrahydropyrane ring, as supported by the HMBC correlations from H_2_-8” to C-9”, C-10”, and C-11” and from H_2_-7” to C-2” and C-4”, as well as the ^1^H–^1^H COZY correlation of H_2_-7”/H_2_-8”. Thus, the planar structure of **4** was determined.

The lack of optical rotation and no obviously observed Cotton effects in the experimental ECD spectrum suggested that compound **4** was also a racemic mixture, which was then subjected to the Daicel IC column by chiral HPLC resolution (Figure 3), thus affording a pair of enantiomers, (±)-asperteretone D (**4a/4b**). The ECD spectra of (−)-asperteretone D (**4a**) and (+)-asperteretone D (**4b**) matched well with those of (−)-asperteretone B (**2a**) and (+)-asperteretone B (**2b**) (Figure 5), respectively, indicating that the absolute structures of **4a** and **4b** should be 4*S*- and 4*R*-configuration, respectively.

Most importantly, the ^1^H and ^13^C NMR data in CDCl_3_ (Supplementary Materials, Figures S39, S40) of **4** were identical to those of (4*S*)-4-decarboxylflavipesolide C (Sun K. et al., 2018), indicating that (4*S*)-4-decarboxylflavipesolide C should be revised to **4** (Figure 6), as supported by the strong correlations from H_2_-5 (δ_H_ 3.58 and 3.99) to C-4 (δ_C_ 102.2) and from H-2' (δ_H_ 7.43) to C-2 (δ_C_ 128.9) in the HMBC spectrum of (4*S*)-4-decarboxylflavipesolide C, whose situation was just the same as that of (±)-asperteretal D (Sun Y. et al., 2018). The minor specific rotation value {[α]${}_{\text{D}}^{25}$ −18 (*c* 0.1, CH_2_Cl_2_)} of (4*S*)-4-decarboxylflavipesolide C was obviously different from those of **4a** {[α]${}_{\text{D}}^{25}$ −120 (*c* 0.1, CH_2_Cl_2_)} and **4b** {[α]${}_{\text{D}}^{25}$ +116 (*c* 0.1, CH_2_Cl_2_)}, indicating that (4*S*)-4-decarboxylflavipesolide C should be a racemic mixture with unsymmetrical amounts rather than a pure substance.

Asperteretone E (**5**), obtained as a white, amorphous powder, was found to have a molecular formula of C_23_H_26_O_6_ with 11 degrees of unsaturation, as deduced from the HRESIMS analysis at *m/z* 421.1626 [M + Na]^+^ (calcd for C_23_H_26_O_6_Na, 421.1627). Side-by-side comparison of the ^1^H and ^13^C NMR data (Tables 1, 2) of **5** with those of **2**, suggesting that they shared the same core skeleton, with the only difference being that the Δ^8^″, 9″ double bond in **2** was replaced by a methylene (δ_C_ 44.9, C-8”) and an oxygenated tertiary carbon (δ_C_ 71.5, C-9”) in **5**, as supported by the HMBC correlations (Figure 2) from H_3_-10” to C-8”, C-9”, and C-11”. Thus, the structure of compound **5** was determined.

The optical rotation of zero in MeOH and inapparent Cotton effects in the ECD curve highlighted that **5** was racemic. Unluckily, despite for many attempts for several chiral columns using various mobile phase systems, we still failed to obtain the enantiomers of **5**, which might own to that the rapid interconversion of these two enantiomers in the solvents prevented the separation on chiral columns.

**Scheme 1:**
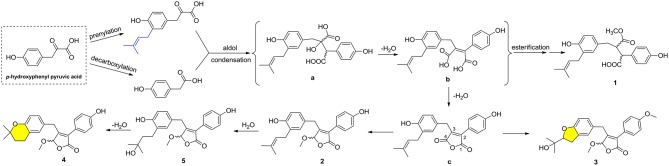
Plausible biogenetic pathways for compounds **1**–**5**.

Compounds **1–5** represented two special classes of 7,8-dimeric phenylpropanoids with unexpected architectures, and their plausible biogenetic pathways were proposed as follows (Scheme 1): two molecules, *p*-hydroxyphenyl pyruvic acid, underwent prenylation and decarboxylation reactions, respectively, followed by aldol condensation and dehydration reactions to create intermediate **b**. Alternatively, a further dehydration reaction of **b** could generate an acid anhydride-containing intermediate **c**, which could furnish **2–5** through a series of reduction, cyclization, esterification, and so on. Meanwhile, the esterification at C-4 and reduction of Δ^2, 3^ double bond could from **1**, which was identified as a crucial biogenetically related metabolite, was the first report of 2,3-disubstituted butenolide derivatives with an unexpected cleavage of oxygen bridge between C-1 and C-4. This finding would greatly expand the chemical space and biosynthesis study for butenolide derivatives.

### Biological evaluation of compounds 1a/1b–4a/4b and 5

Compounds **1a/1b–4a/4b** and **5** were evaluated for the α-glucosidase inhibitory activity. As shown in Table 3, all the compounds exhibited potent inhibitory potency against α-glucosidase, with IC_50_ values ranging from 15.7 ± 1.1 to 53.1 ± 1.4 μM, which was much lower than that of the positive control acarbose (IC_50_ = 154.7 ± 8.1 μM). All enantiomers displayed nearly horizontal IC_50_ values against α-glucosidase inhibitory activity, indicating that the difference of chirality might have a negligible impact on the activity. Most importantly, compounds **1a/1b–4a/4b** and **5** may provide novel chemical scaffolds for the discovery of new α-glucosidase inhibitors.

**Table 3:** α-Glucosidase inhibitory activity of **1a/1b**−**4a/4b** and **5**.

**Compound**	**α-glucosidase inhibitory activity (IC_50_, μM)**
**1a**	45.4 ± 3.8
**1b**	53.1 ± 1.4
**2a**	17.3 ± 2.4
**2b**	19.2 ± 1.9
**3a**	52.2 ± 4.6
**3b**	49.8 ± 5.7
**4a**	15.7 ± 1.1
**4b**	18.9 ± 2.3
**5**	48.9 ± 7.3
acarbose	154.7 ± 8.1

**Figure 7:**
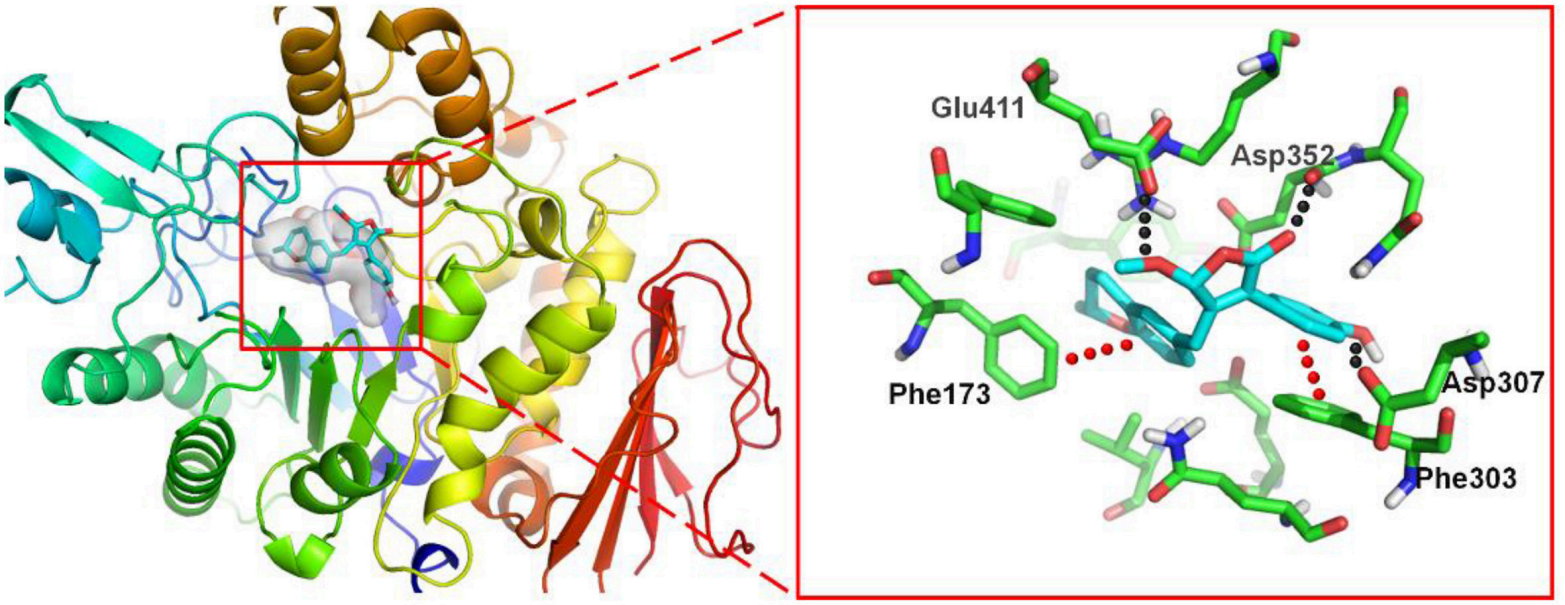
3D docking pose shows the interaction of **4a** in the binding site of the enzyme from *Saccharomyces cerevisiae* (PDB ID: 3A4A): the left side shows the global view of the enzyme; the right side shows an expanded view of **4a** in the binding site.

To investigate the binding mode of these compounds with α-glucosidase, molecular docking study was carried out by using the SYBYL 2.0 software. Due to the unavailable of crystal structure of α-glucosidase from *Saccharomyces cerevisiae*, the crystal structure of isomaltase (PDB ID: 3A4A) from *S. cerevisiae*, which is 84% similar to that of *S. cerevisiae* α-glucosidase, was conducted as docking model (Shen et al., 2015). The theoretical binding mode between **4a** and the enzyme was shown in Figure 7. Compound **4a** adopted a “V-shaped” conformation in the pocket. Detailed analysis showed that the phenolic group and benzopyran group of **4a** formed π-π stacking interaction with the residue Phe303 and Phe173, respectively. It was also shown that the residue Asp307, Asp352, and Glu411 formed key hydrogen bonds with **4a**, which were the main interactions between **4a** and the enzyme. All these interactions helped **4a** to anchor in the binding site of the enzyme.

## Conclusions

In conclusion, nine novel butenolide derivatives belonging to two undescribed structural types, including four pairs of enantiomers (**1a/1b**–**4a/4b**) and a racemate (**5**), were isolated from the coral**-**associated fungus *Aspergillus terreus*. More importantly, (±)-asperteretal D and (4*S*)-4-decarboxylflavipesolide C were structurally revised to **2a**/**2b** and **4**, respectively. This study further enriched secondary metabolites in the *Aspergillus* species and was also a strong structural supplement to the new class of γ-butenolides. In addition, bioactivity evaluation results showed that all the isolates exhibited potent α-glucosidase inhibitory activity with IC_50_ values ranging from 15.7 ± 1.1 to 53.1 ± 1.4 μM. On the background that DM is becoming a global public health problem and more new effective therapeutic agents are in the urgent need, our findings provide a basis for further development and utilization of butenolide derivatives as source of potential α-glucosidase inhibitors as therapeutic agents for type-2 diabetes mellitus.

## Author contributions

ML and CQ contributed to the extraction, isolation, identification, and manuscript preparation. WS contributed to the α-glucosidase inhibitory activity test. LS contributed to the fungal isolation and fermentation. JW advised and assisted Liu's experiments. JL contributed to the ECD calculations. YL contributed to the molecular docking experiment. YX contributed to the structure identification of isolated compounds. ZH guided Liu's experiments and wrote the manuscript. YZ designed the experiments and revised the manuscript.

### Conflict of interest statement

The authors declare that the research was conducted in the absence of any commercial or financial relationships that could be construed as a potential conflict of interest.

## References

[B1] Baron A. D. (1998). Postprandial hyperglycaemia and α-glucosidase inhibitors. Diabetes Res. Clin. Pr. 40, 51–55. 10.1016/S0168-8227(98)00043-6 9740503

[B2] He Y. Hu Z. Li Q. Huang J. Li X. N. Zhu H.. (2017a). Bioassay-guided isolation of antibacterial metabolites from *Emericella* sp. TJ29. J. Nat. Prod. 80, 2399–2405. 10.1021/acs.jnatprod.7b00077 28901763

[B3] He Y. Hu Z. Sun W. Li Q. Li X. N. Zhu H.. (2017b). Spiroaspertrione A, a Bridged Spirocyclic meroterpenoid, as a potent potentiator of oxacillin against methicillin-resistant *Staphylococcus aureus* from *Aspergillus* sp. TJ23. J. Org. Chem. 82, 3125–3131. 10.1021/acs.joc.7b00056 28219242

[B4] He Y. Zheng M. Li Q. Hu Z. Zhu H. Liu J. (2017c). Asperspiropene A, a novel fungal metabolite as an inhibitor of cancer-associated mutant isocitrate dehydrogenase 1. Org. Chem. Front. 4, 1137–1144. 10.1039/c6qo00847j

[B5] Hu Z. Wu Y. Xie S. Sun W. Guo Y. Li X. N.. (2017). Phomopsterones A and B, two functionalized ergostane-type steroids from the endophytic fungus *Phomopsis* sp. TJ507A. Org. Lett. 19, 258–261. 10.1021/acs.orglett.6b03557 28004944

[B6] Hu Z. X. Xue Y. B. Bi X. B. Zhang J. W. Luo Z. W. Li X. N.. (2014). Five new secondary metabolites produced by a marine-associated fungus, *Daldinia eschscholzii*. Mar. Drugs 12, 5563–5575. 10.3390/md12115563 25419997 PMC4245545

[B7] Hung H. Y. Qian K. Morris-Natschke S. L. Hsu C. S. Lee K. H. (2012). Recent discovery of plant-derived anti-diabetic natural products. Nat. Prod. Rep. 29, 580–606. 10.1039/C2NP00074A 22491825

[B8] Kao C. C. Wu P. C. Wu C. H. Chen L. K. Chen H. H. Wu M. S.. (2016). Risk of liver injury after α-glucosidase inhibitor therapy in advanced chronic kidney disease patients. Sci. Rep. 6:18996. 10.1038/srep18996 26751038 PMC4707434

[B9] Kim K. Y. Nam K. A. Kurihara H. Kim S. M. (2008). Potent α-glucosidase inhibitors purified from the red alga *Grateloupia elliptica*. Phytochemistry 69, 2820–2825. 10.1016/j.phytochem.2008.09.007 18951591

[B10] Kopelman P. G. (2000). Obesity as a medical problem. Nature 404, 635–643. 10.1038/35007508 10766250

[B11] Lauritano C. Ianora A. (2016). Marine organisms with anti-diabetes properties. Mar. Drugs 14:220 10.3390/md14120220 PMC5192457 27916864

[B12] Liu M. Sun W. Wang J. He Y. Zhang J. Li F.. (2018a). Bioactive secondary metabolites from the marine-associated fungus *Aspergillus terreus*. Bioorg. Chem. 80, 525–530. 10.1016/j.bioorg.2018.06.029 30014920

[B13] Liu M. Zhou Q. Wang J. Liu J. Qi C. Lai Y. (2018b). Anti-inflammatory butenolide derivatives from the coral-derived fungus *Aspergillus terreu*s and structure revisions of aspernolides D and G, butyrolactone VI and 4',8”-diacetoxy butyrolactone VI. RSC Adv. 8, 13040–13047. 10.1039/c8ra01840e PMC9079733 35541261

[B14] Miertus S. Scrocc E. Tomasi J. (1981). Electrostatic interaction of a solute with a continuum. A direct utilizaion of AB initio molecular potentials for the prevision of solvent effects. J. Chem. Phys. 55, 117–129. 10.1016/0301-0104(81)85090-2

[B15] Shen X. Saburi W. Gai Z. Kato K. Ojima-Kato T. Yu J.. (2015). Structural analysis of the α-glucosidase HaG provides new insights into substrate specificity and catalytic mechanism. Acta Cryst. 71, 1382–1391. 10.1107/S139900471500721X 26057678

[B16] Smith S. G. Goodman J. M. (2010). Assigning stereochemistry to single diastereoisomers by GIAO NMR calculation: the DP4 probability. J. Am. Chem. Soc. 132, 12946–12959. 10.1021/ja105035r 20795713

[B17] Sun K. Zhu G. Hao J. Wang Y. Zhu W. (2018). Chemical-epigenetic method to enhance the chemodiversity of the marine algicolous fungus, *Aspergillus terreus* OUCMDZ-2739. Tetrahedron 74, 83–87. 10.1016/j.tet.2017.11.039

[B18] Sun Y. Liu J. Li L. Gong C. Wang S. Yang F. (2018). New butenolide derivatives from the marine sponge-derived fungus *Aspergillus terreus*. Bioorg. Med. Chem. Lett. 28, 315–318. 10.1016/j.bmcl.2017.12.049 29295795

[B19] Tähtinen P. Bagno A. Klika K. D. Pihlaja K. (2003). Modeling NMR parameters by DFT methods as an aid to the conformational analysis of cis-fused 7a(8a)-methyl octa(hexa)hydrocyclopenta[*d*][1,3]oxazines and [3,1]benzoxazines. J. Am. Chem. Soc. 125, 4609–4618. 10.1021/ja021237t 12683833

[B20] Yang B. Sun W. Wang J. Lin S. Li X. N. Zhu H. (2018). A new breviane spiroditerpenoid from the marine-derived fungus *Penicillium* sp. TJ403-1. Mar. Drugs 16:110 10.3390/md16040110 PMC5923397 29596354

[B21] Yin Z. Zhang W. Feng F. Zhang Y. Kang W. (2014). α-glucosidase inhibitors isolated from medicinal plants. Food Sci. Hum. Wellness 3, 136–174. 10.1016/j.fshw.2014.11.003

