# Supplementary material for: α-Glucosidase Inhibitors From the Coral-Associated Fungus Aspergillus terreus
Source: Front Chem. 2018 Sep 13;6:422. doi: 10.3389/fchem.2018.00422 (PMC6146087; doi:10.3389/fchem.2018.00422)
Supplement: Supplementary file 1 [file Table_1.DOC]

**Supplementary Material**

***α*-Glucosidase Inhibitors from the Coral-Associated Fungus *Aspergillus terreus***

**Mengting Liu^1‡^, Changxing Qi^1‡^, Weiguang Sun^1‡^,** **Ling Shen^1^, Jianping Wang^1^, Junjun Liu^1^, Yongji Lai^2^, Yongbo Xue^1^, Zhengxi Hu^1^* and Yonghui Zhang^1^***

^1^Hubei Key Laboratory of Natural Medicinal Chemistry and Resource Evaluation, School of Pharmacy, Tongji Medical College, Huazhong University of Science and Technology, Wuhan, China

^2^Department of Pharmacy, the Central Hospital of Wuhan, Wuhan, China

*** Correspondence:** Zhengxi Hu: [hzx616@126.com](mailto:hzx616@126.com); Yonghui Zhang: zhangyh@mails.tjmu.edu.cn

**CONTENTS**

[**Figure S1.** ^1^H NMR spectrum of compound **1** (Recorded in methanol-*d*_4_) 1](#__RefHeading___Toc514324848)

[**Figure S2.** ^13^C NMR spectrum of compound **1** (Recorded in methanol-*d*_4_) 2](#__RefHeading___Toc514324849)

[**Figure S3.** DEPT spectrum of compound **1** (Recorded in methanol-*d*_4_) 3](#__RefHeading___Toc514324850)

[**Figure S4.** HSQC spectrum of compound **1** (Recorded in methanol-*d*_4_) 4](#__RefHeading___Toc514324851)

[**Figure S5.** HMBC spectrum of compound **1** (Recorded in methanol-*d*_4_) 5](#__RefHeading___Toc514324852)

[**Figure S6.** ^1^H–^1^H COSY spectrum of compound **1** (Recorded in methanol-*d*_4_) 6](#__RefHeading___Toc514324853)

[**Figure S7.** NOESY spectrum of compound **1** (Recorded in methanol-*d*_4_) 7](#__RefHeading___Toc514324854)

[**Figure S8.** HRESIMS spectrum of compound **1** 8](#__RefHeading___Toc514324855)

[**Figure S9.** UV spectrum of compound **1** 9](#__RefHeading___Toc514324856)

[**Figure S10.** IR spectrum of compound **1** 10](#__RefHeading___Toc514324857)

[**Figure S11.** ^1^H NMR spectrum of compound **2** (Recorded in CDCl_3_) 11](#__RefHeading___Toc514324858)

[**Figure S12.** ^13^C NMR spectrum of compound **2** (Recorded in CDCl_3_) 12](#__RefHeading___Toc514324859)

[**Figure S13.** DEPT spectrum of compound **2** (Recorded in CDCl_3_) 13](#__RefHeading___Toc514324860)

[**Figure S14.** HSQC spectrum of compound **2** (Recorded in CDCl_3_) 14](#__RefHeading___Toc514324861)

[**Figure S15.** HMBC spectrum of compound **2** (Recorded in CDCl_3_) 15](#__RefHeading___Toc514324862)

[**Figure S16.** Enlarged HMBC analysis of compound **2** (Recorded in CDCl_3_) 16](#__RefHeading___Toc514324863)

[**Figure S17.** Enlarged HMBC analysis of compound **2** (Recorded in CDCl_3_) 17](#__RefHeading___Toc514324864)

[**Figure S18.** Enlarged HMBC analysis of compound **2** (Recorded in CDCl_3_) 18](#__RefHeading___Toc514324865)

[**Figure S19.** Enlarged HMBC analysis of compound **2** (Recorded in CDCl_3_) 19](#__RefHeading___Toc514324866)

[**Figure S20.** ^1^H–^1^H COSY spectrum of compound **2** (Recorded in CDCl_3_) 20](#__RefHeading___Toc514324867)

[**Figure S21.** HRESIMS spectrum of compound **2** 21](#__RefHeading___Toc514324868)

[**Figure S22.** UV spectrum of compound **2** 22](#__RefHeading___Toc514324869)

[**Figure S23.** IR spectrum of compound **2** 23](#__RefHeading___Toc514324870)

[**Figure S24.** ^1^H NMR spectrum of compound **3** (Recorded in methanol-*d*_4_) 24](#__RefHeading___Toc514324871)

[**Figure S25.** ^13^C NMR spectrum of compound **3** (Recorded in methanol-*d*_4_) 25](#__RefHeading___Toc514324872)

[**Figure S26.** DEPT spectrum of compound **3** (Recorded in methanol-*d*_4_) 26](#__RefHeading___Toc514324873)

[**Figure S27.** HSQC spectrum of compound **3** (Recorded in methanol-*d*_4_) 27](#__RefHeading___Toc514324874)

[**Figure S28.** HMBC spectrum of compound **3** (Recorded in methanol-*d*_4_) 28](#__RefHeading___Toc514324875)

[**Figure S29.** ^1^H–^1^H COSY spectrum of compound **3** (Recorded in methanol-*d*_4_) 29](#__RefHeading___Toc514324876)

[**Figure S30.** HRESIMS spectrum of compound **3** 30](#__RefHeading___Toc514324877)

[**Figure S31.** UV spectrum of compound **3** 31](#__RefHeading___Toc514324878)

[**Figure S32.** IR spectrum of compound **3** 32](#__RefHeading___Toc514324879)

[**Figure S33.** ^1^H NMR spectrum of compound **4** (Recorded in methanol-*d*_4_) 33](#__RefHeading___Toc514324880)

[**Figure S34.** ^13^C NMR spectrum of compound **4** (Recorded in methanol-*d*_4_) 34](#__RefHeading___Toc514324881)

[**Figure S35.** DEPT spectrum of compound **4** (Recorded in methanol-*d*_4_) 35](#__RefHeading___Toc514324882)

[**Figure S36.** HSQC spectrum of compound **4** (Recorded in methanol-*d*_4_) 36](#__RefHeading___Toc514324883)

[**Figure S37.** HMBC spectrum of compound **4** (Recorded in methanol-*d*_4_) 37](#__RefHeading___Toc514324884)

[**Figure S38.** ^1^H–^1^H COSY spectrum of compound **4** (Recorded in methanol-*d*_4_) 38](#__RefHeading___Toc514324885)

[**Figure S39.** ^1^H NMR spectrum of compound **4** (Recorded in CDCl_3_) 39](#__RefHeading___Toc514324886)

[**Figure S40.** ^13^C NMR spectrum of compound **4** (Recorded in CDCl_3_) 40](#__RefHeading___Toc514324887)

[**Figure S41.** HRESIMS spectrum of compound **4** 41](#__RefHeading___Toc514324888)

[**Figure S42.** UV spectrum of compound **4** 42](#__RefHeading___Toc514324889)

[**Figure S43.** IR spectrum of compound **4** 43](#__RefHeading___Toc514324890)

[**Figure S44.** ^1^H NMR spectrum of compound **5** (Recorded in methanol-*d*_4_) 44](#__RefHeading___Toc514324891)

[**Figure S45.** ^13^C NMR spectrum of compound **5** (Recorded in methanol-*d*_4_) 45](#__RefHeading___Toc514324892)

[**Figure S46.** DEPT spectrum of compound **5** (Recorded in methanol-*d*_4_) 46](#__RefHeading___Toc514324893)

[**Figure S47.** HSQC spectrum of compound **5** (Recorded in methanol-*d*_4_) 47](#__RefHeading___Toc514324894)

[**Figure S48.** HMBC spectrum of compound **5** (Recorded in methanol-*d*_4_) 48](#__RefHeading___Toc514324895)

[**Figure S49.** ^1^H–^1^H COSY spectrum of compound **5** (Recorded in methanol-*d*_4_) 49](#__RefHeading___Toc514324896)

[**Figure S50.** HRESIMS spectrum of compound **5** 50](#__RefHeading___Toc514324897)

[**Figure S51.** UV spectrum of compound **5** 51](#__RefHeading___Toc514324898)

[**Figure S52.** IR spectrum of compound **5** 52](#__RefHeading___Toc514324899)

[**Calculated ECD data of compounds 1 and 2** 53](#__RefHeading___Toc514324900)


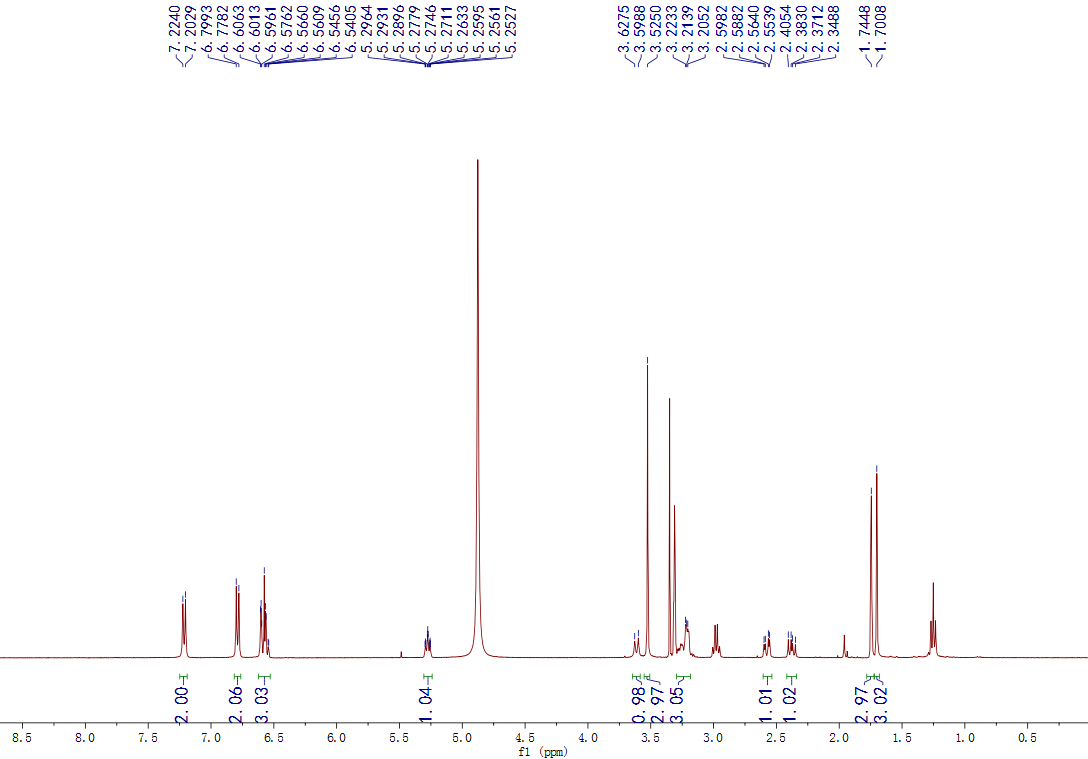


**Figure S1.** ^1^H NMR spectrum of compound **1** (Recorded in methanol-*d*_4_)


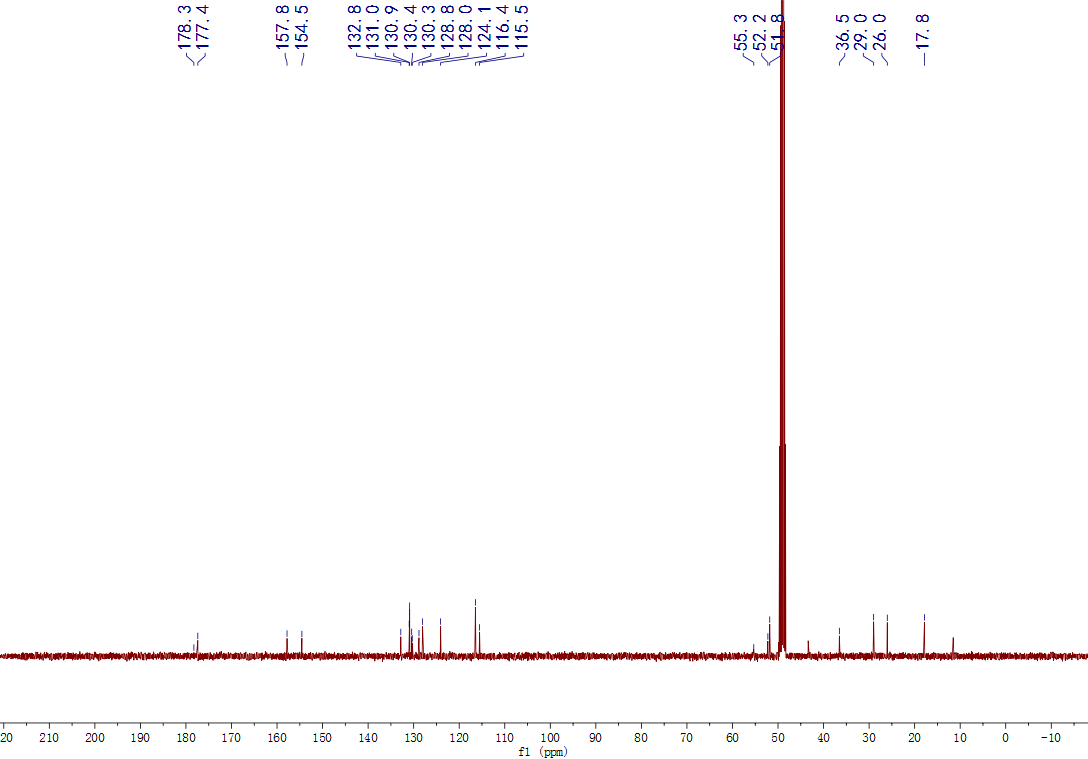


**Figure S2.** ^13^C NMR spectrum of compound **1** (Recorded in methanol-*d*_4_)

**
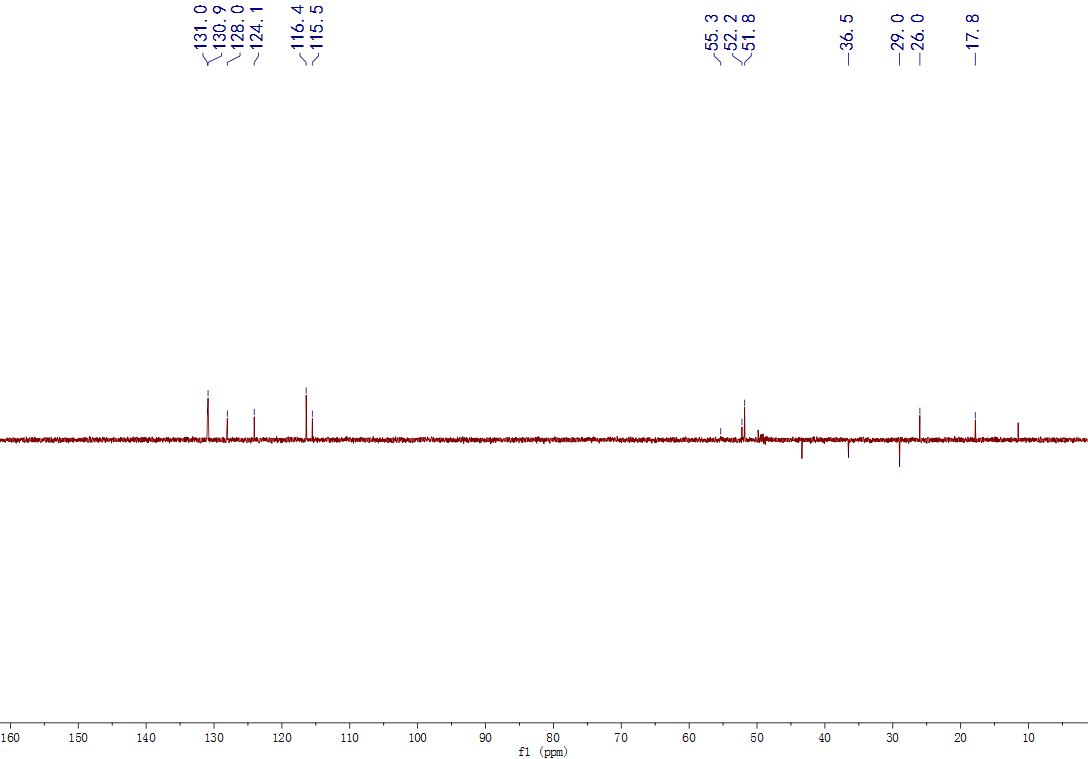
**

**Figure S3.** DEPT spectrum of compound **1** (Recorded in methanol-*d*_4_)

**
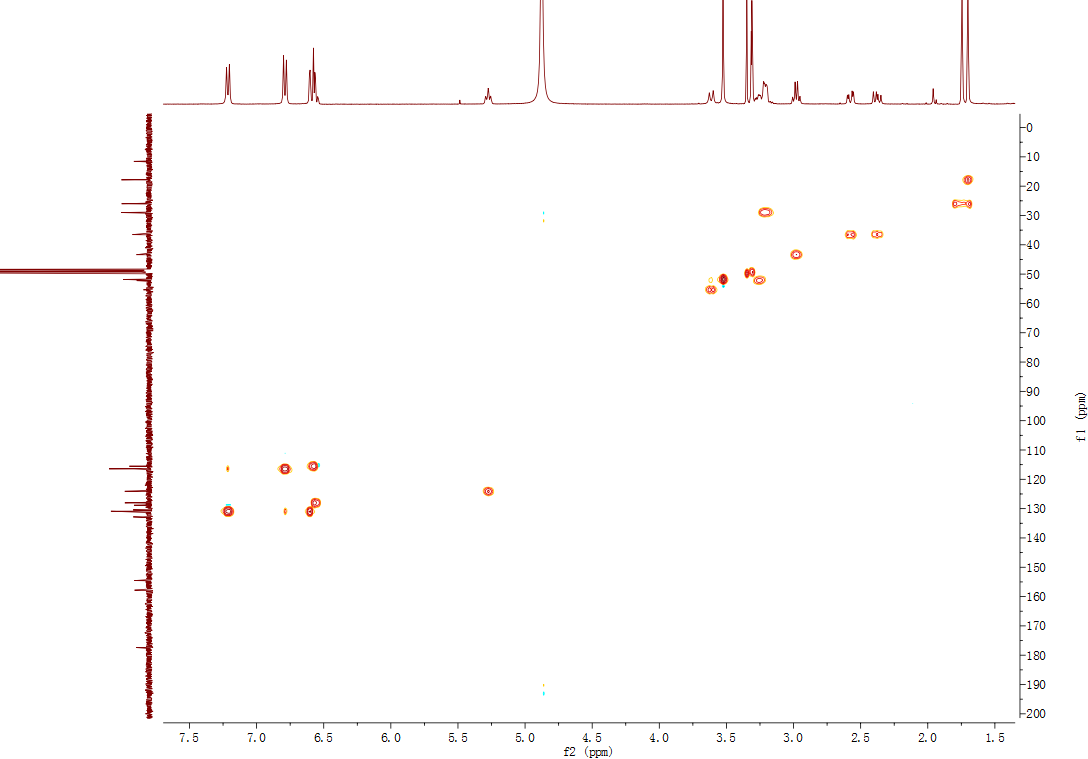
**

**Figure S4.** HSQC spectrum of compound **1** (Recorded in methanol-*d*_4_)

**
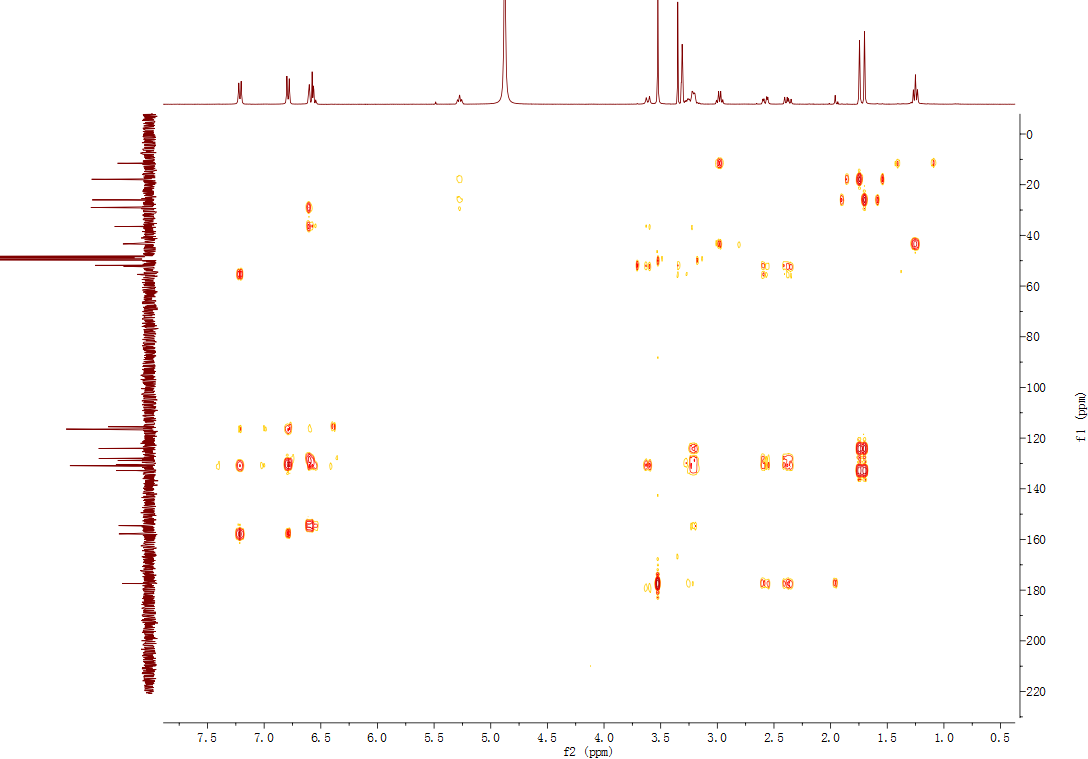
**

**Figure S5.** HMBC spectrum of compound **1** (Recorded in methanol-*d*_4_)

**
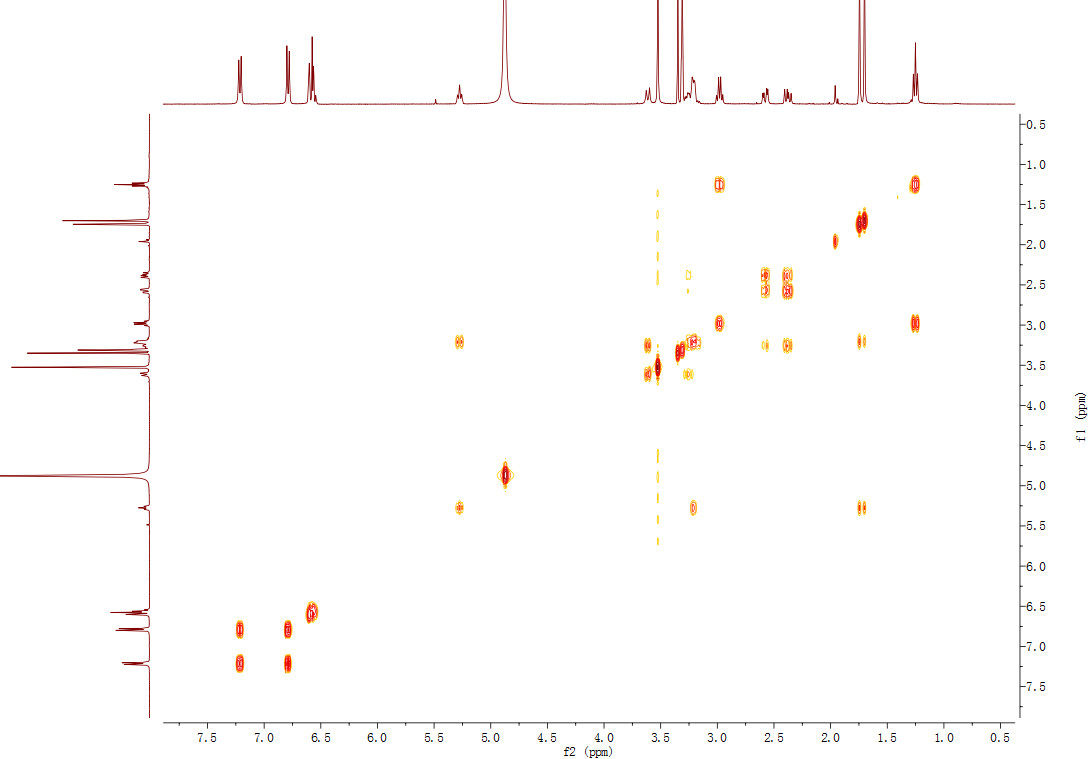
**

**Figure S6.** ^1^H–^1^H COSY spectrum of compound **1** (Recorded in methanol-*d*_4_)

**
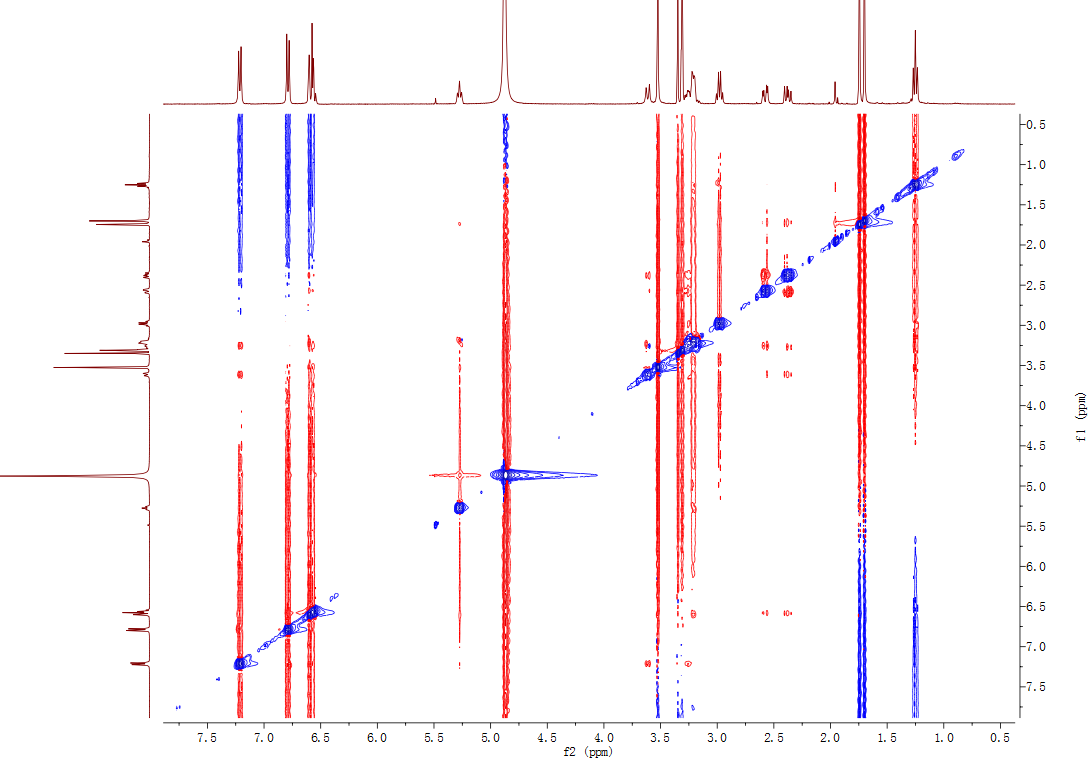
**

**Figure S7.** NOESY spectrum of compound **1** (Recorded in methanol-*d*_4_)

**Figure S8.** HRESIMS spectrum of compound **1**

**Figure S9.** UV spectrum of compound **1**

**Figure S10.** IR spectrum of compound **1**


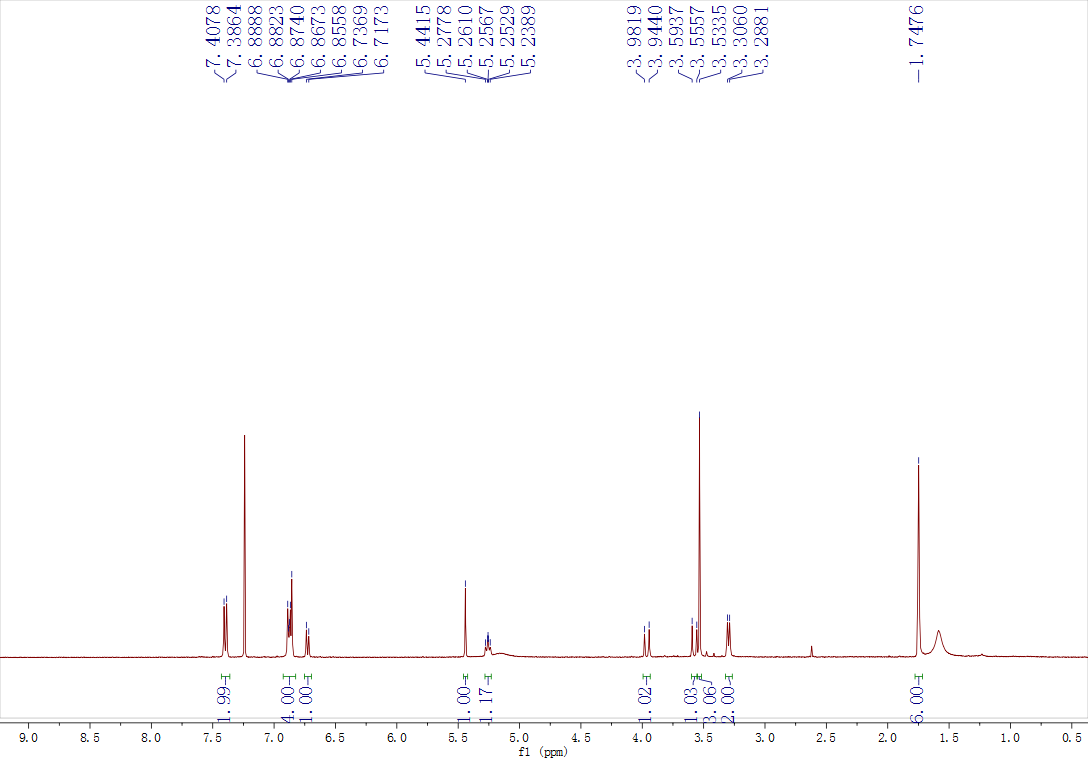


**Figure S11.** ^1^H NMR spectrum of compound **2** (Recorded in CDCl_3_)


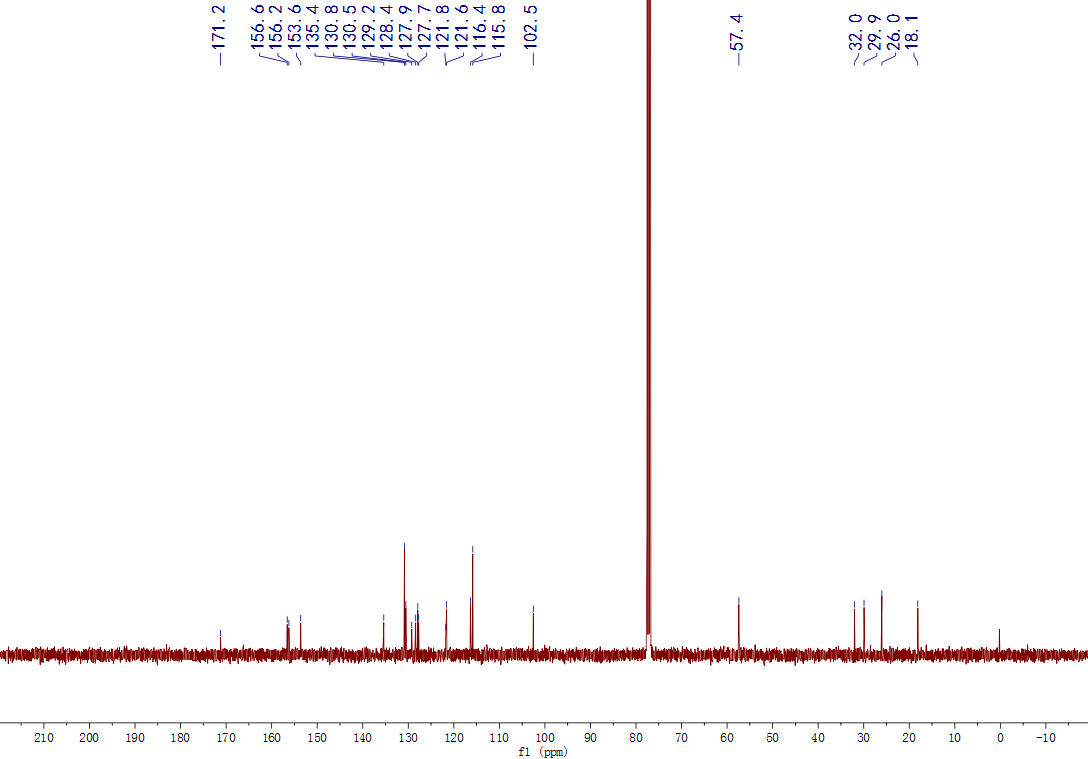


**Figure S12.** ^13^C NMR spectrum of compound **2** (Recorded in CDCl_3_)

**
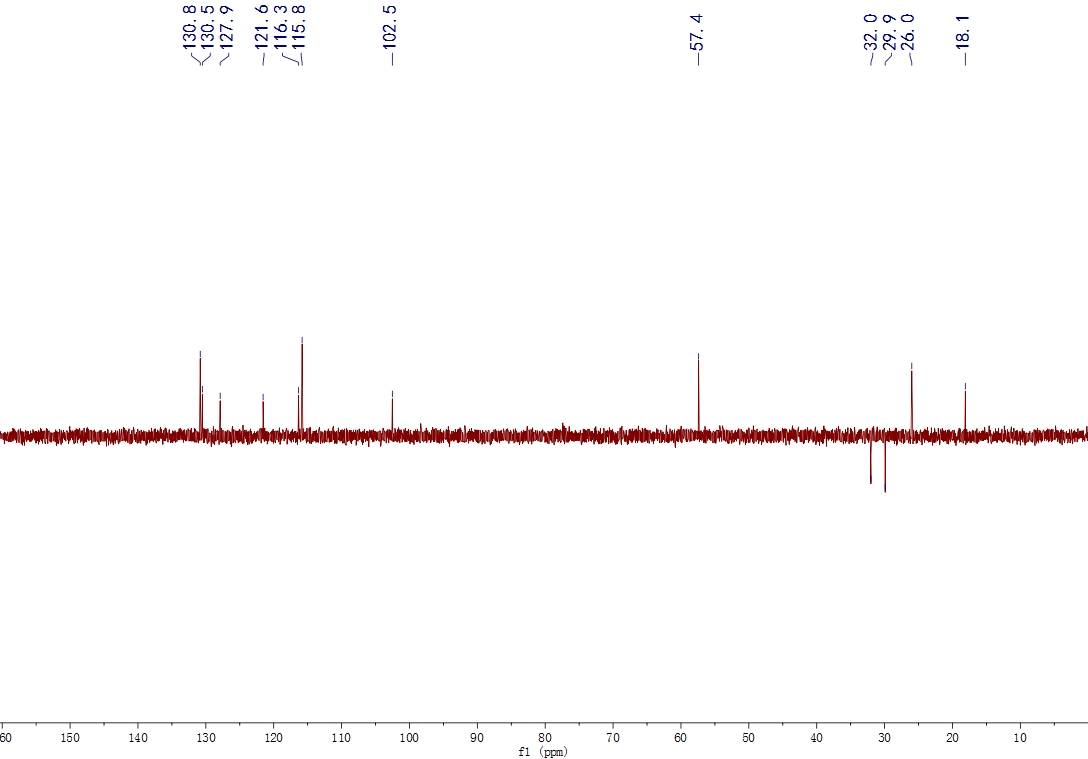
**

**Figure S13.** DEPT spectrum of compound **2** (Recorded in CDCl_3_)

**
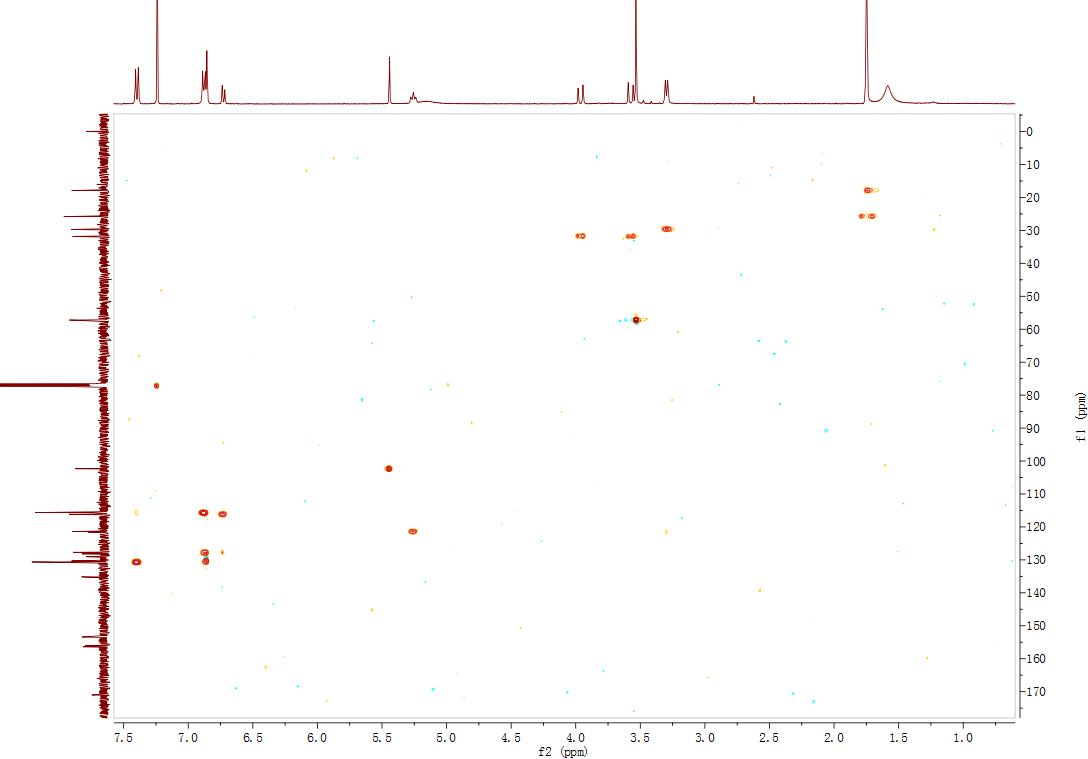
**

**Figure S14.** HSQC spectrum of compound **2** (Recorded in CDCl_3_)

**
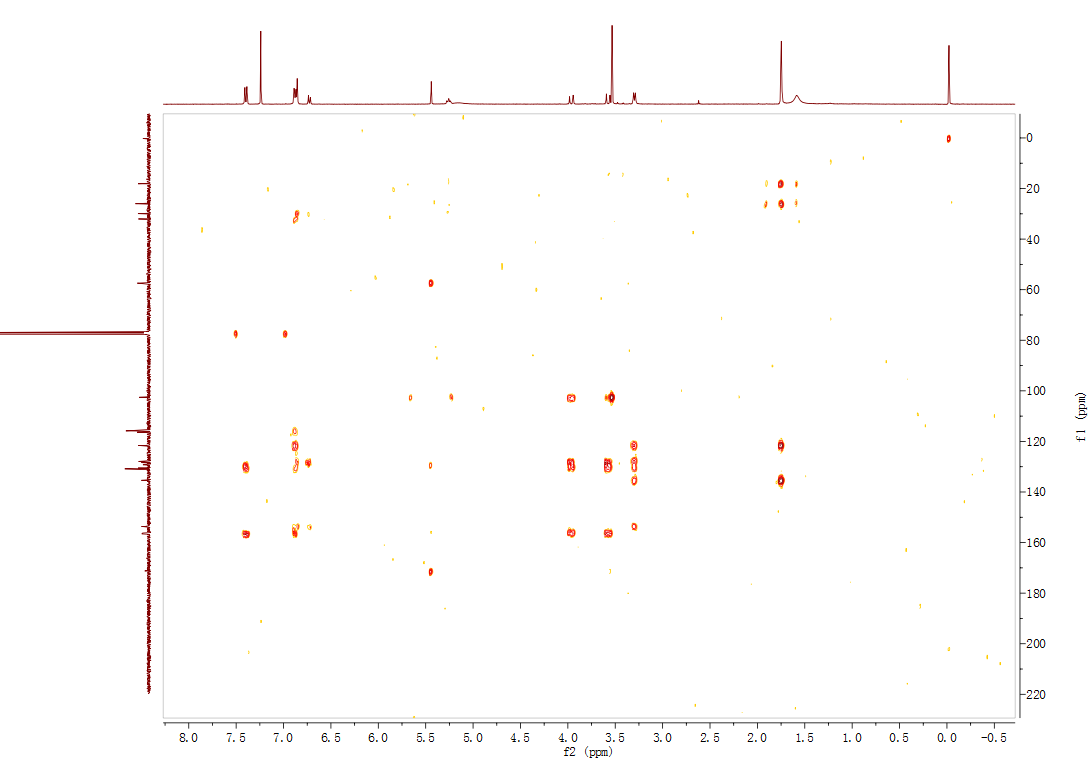
**

**Figure S15.** HMBC spectrum of compound **2** (Recorded in CDCl_3_)


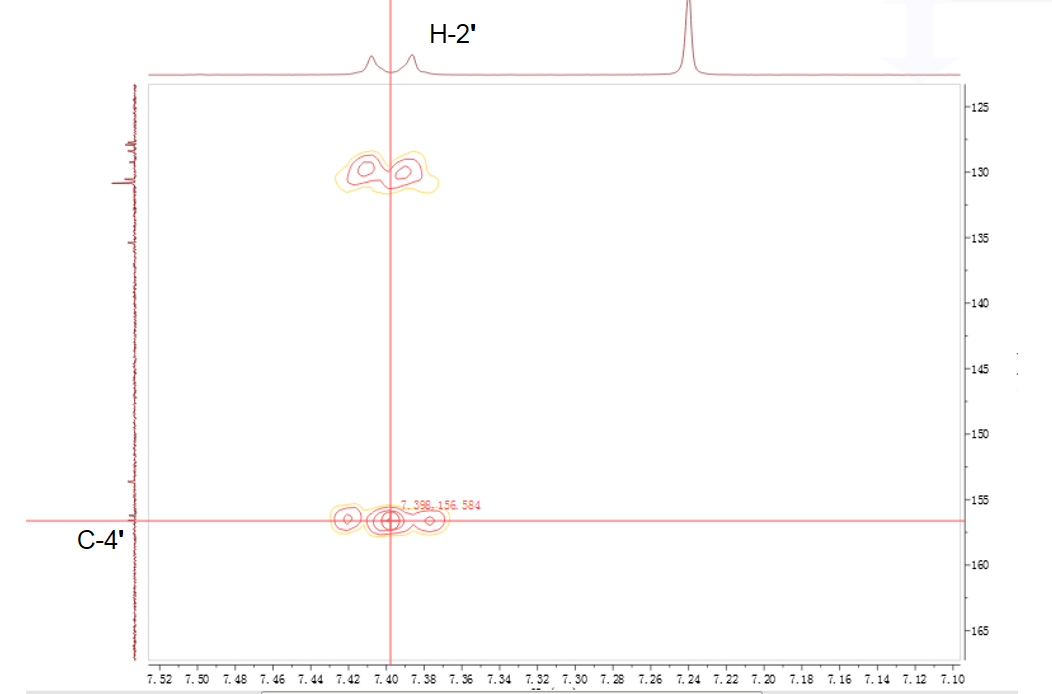


**Figure S16.** Enlarged HMBC analysis of compound **2** (Recorded in CDCl_3_)


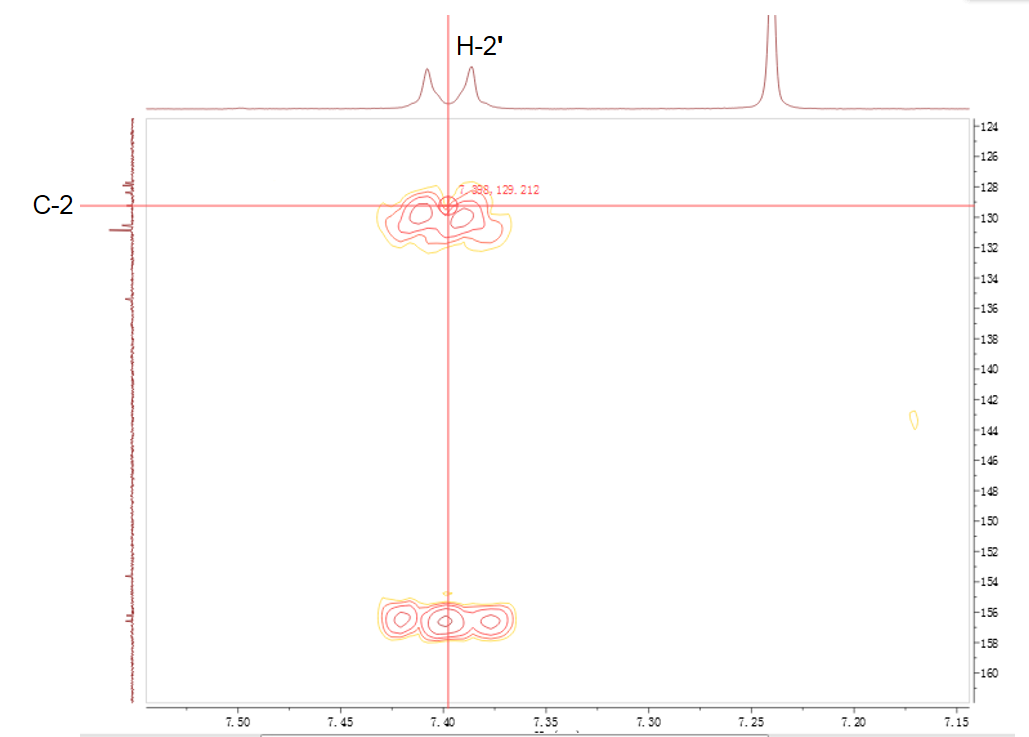


**Figure S17.** Enlarged HMBC analysis of compound **2** (Recorded in CDCl_3_)


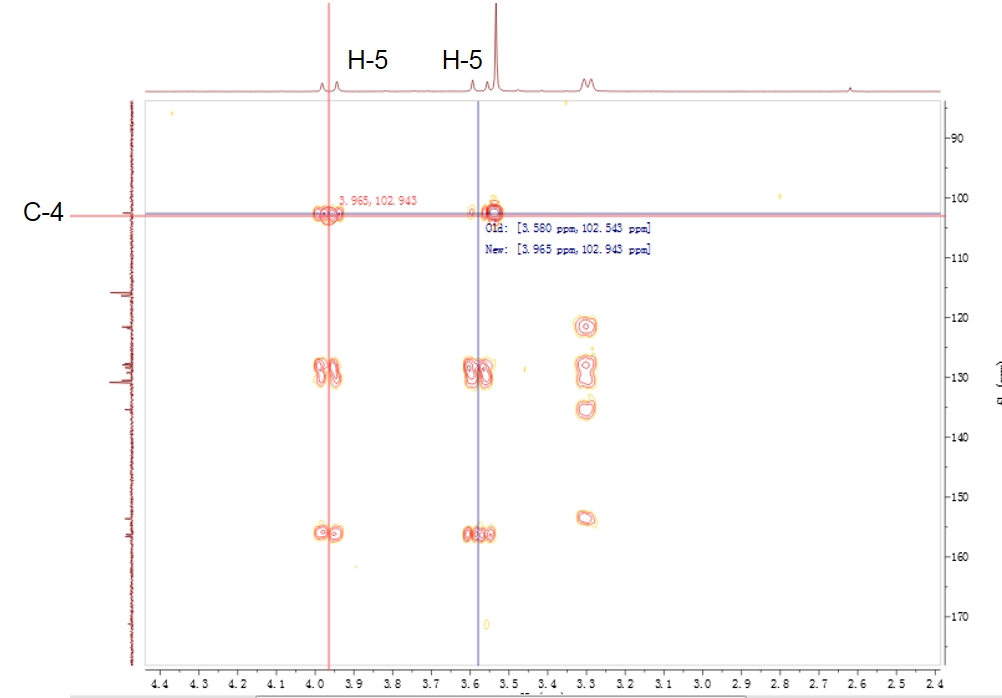


**Figure S18.** Enlarged HMBC analysis of compound **2** (Recorded in CDCl_3_)


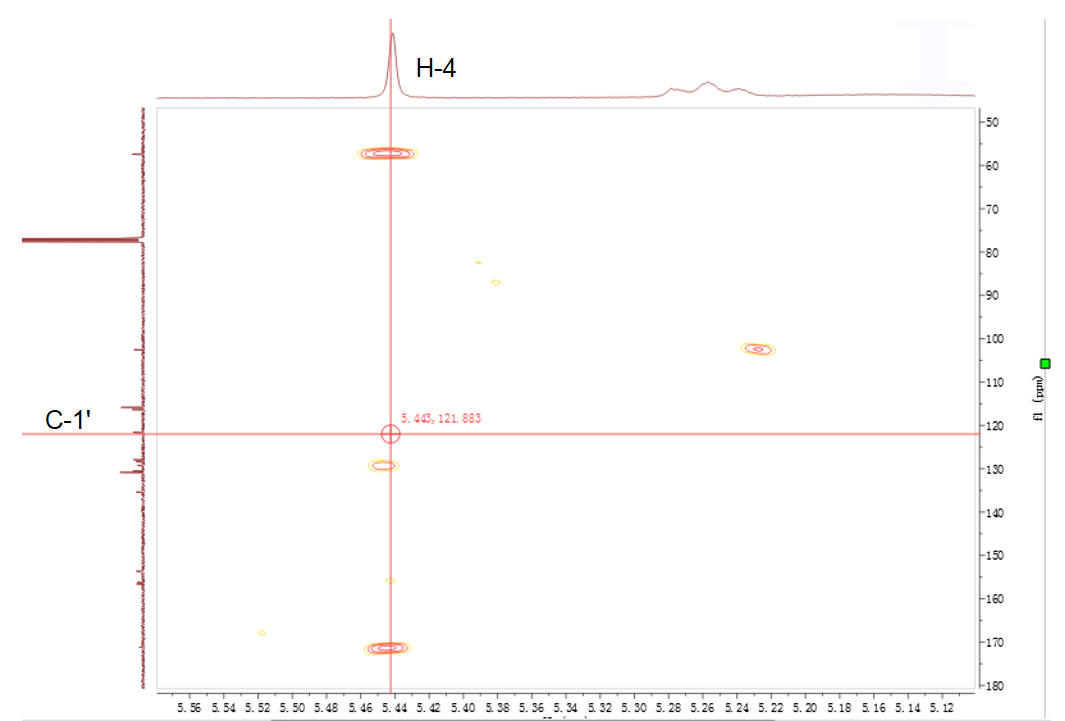


no correlation

**Figure S19.** Enlarged HMBC analysis of compound **2** (Recorded in CDCl_3_)

**
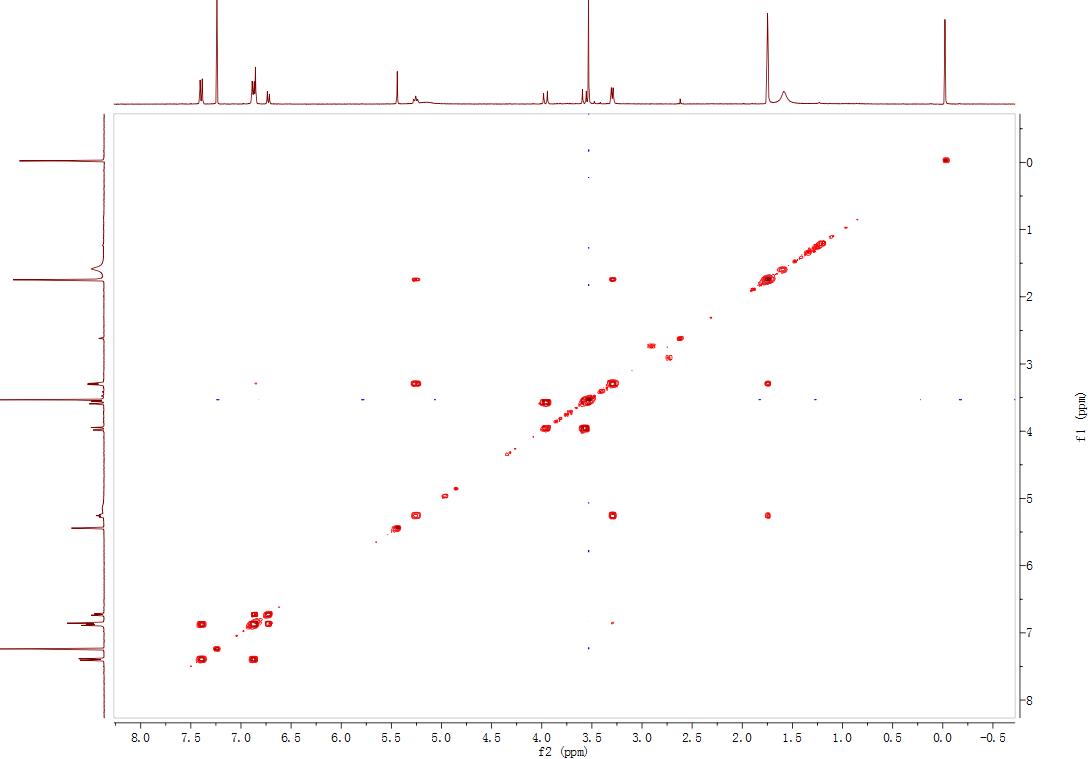
**

**Figure S20.** ^1^H–^1^H COSY spectrum of compound **2** (Recorded in CDCl_3_)

**Figure S21.** HRESIMS spectrum of compound **2**

**Figure S22.** UV spectrum of compound **2**

**Figure S23.** IR spectrum of compound **2**


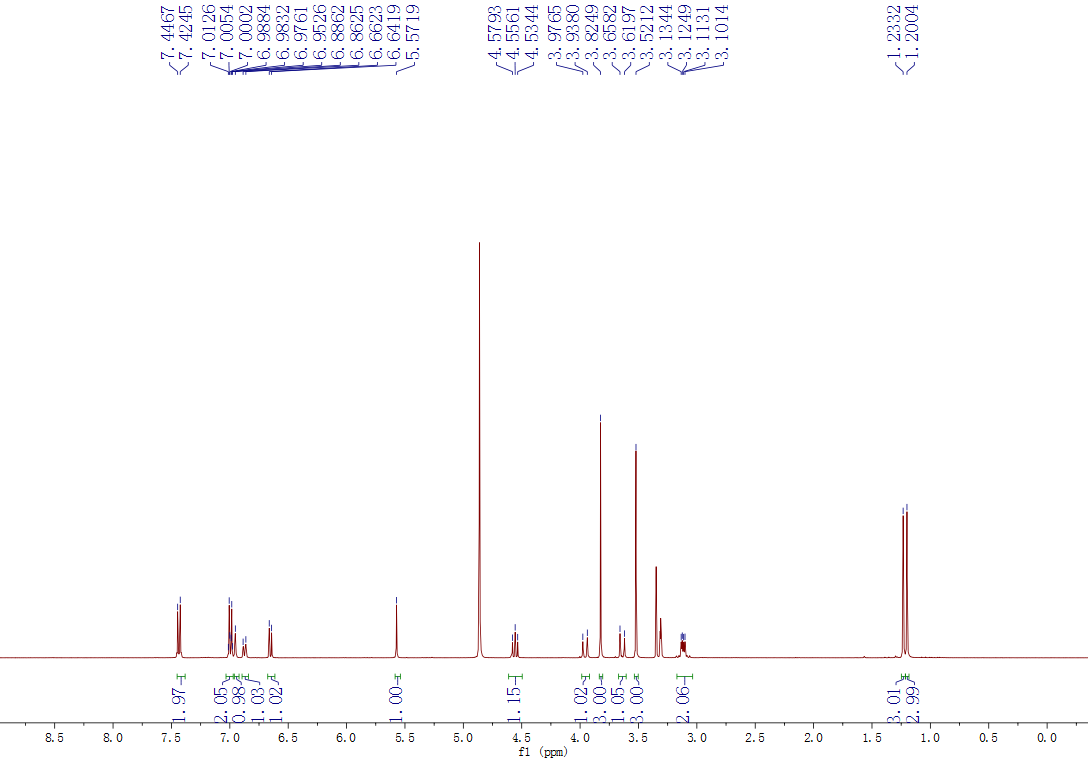


**Figure S24.** ^1^H NMR spectrum of compound **3** (Recorded in methanol-*d*_4_)


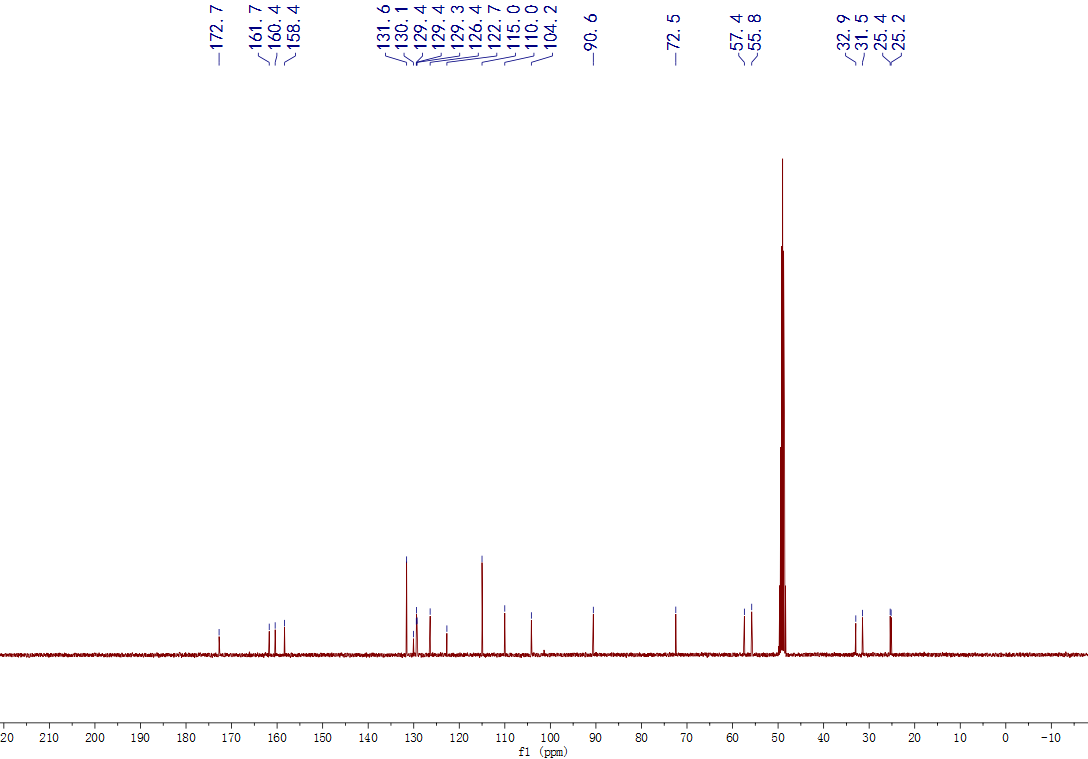


**Figure S25.** ^13^C NMR spectrum of compound **3** (Recorded in methanol-*d*_4_)

**
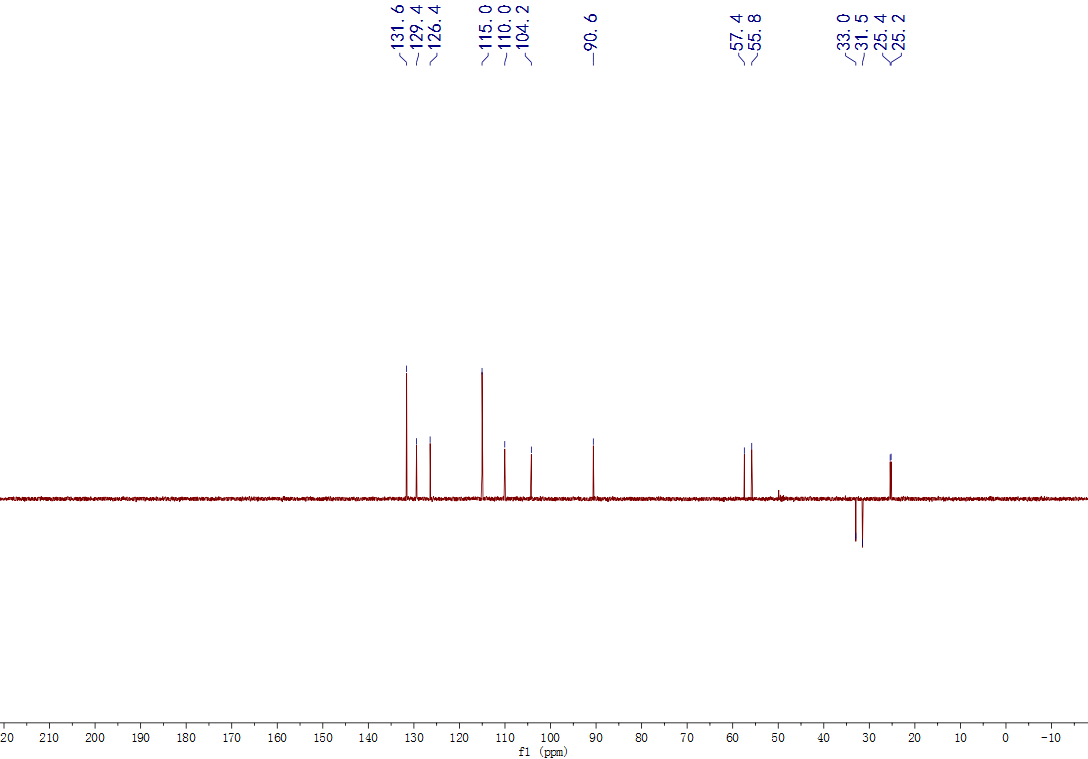
**

**Figure S26.** DEPT spectrum of compound **3** (Recorded in methanol-*d*_4_)

**
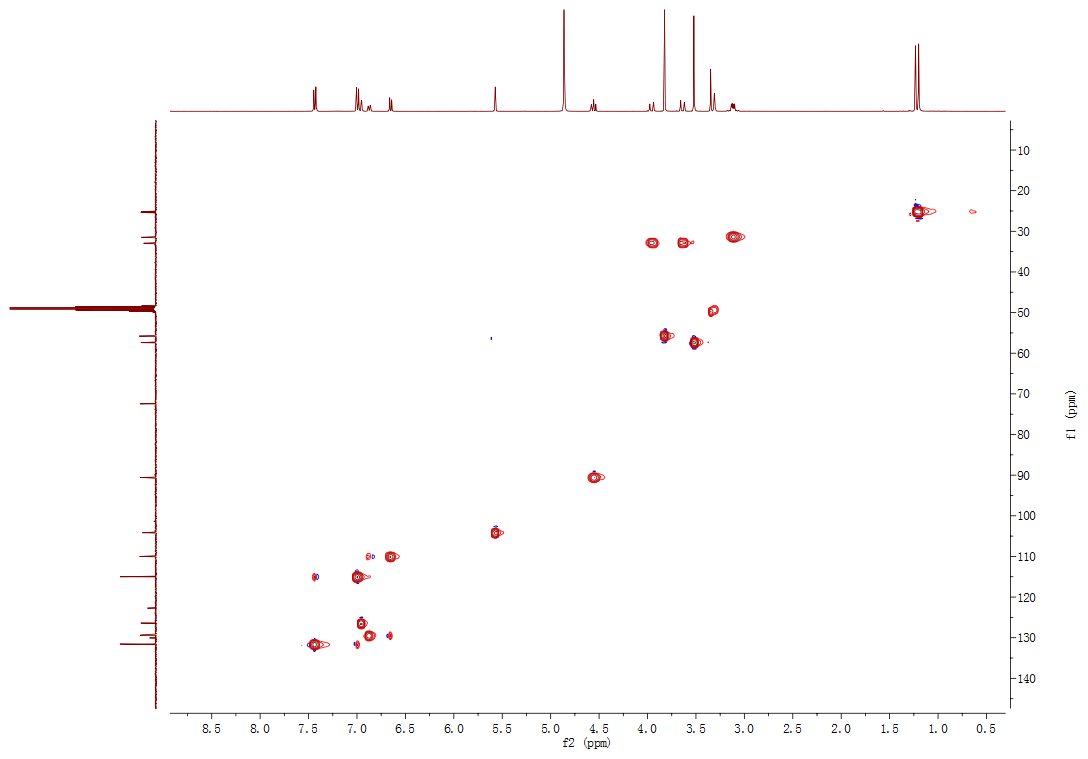
**

**Figure S27.** HSQC spectrum of compound **3** (Recorded in methanol-*d*_4_)

**
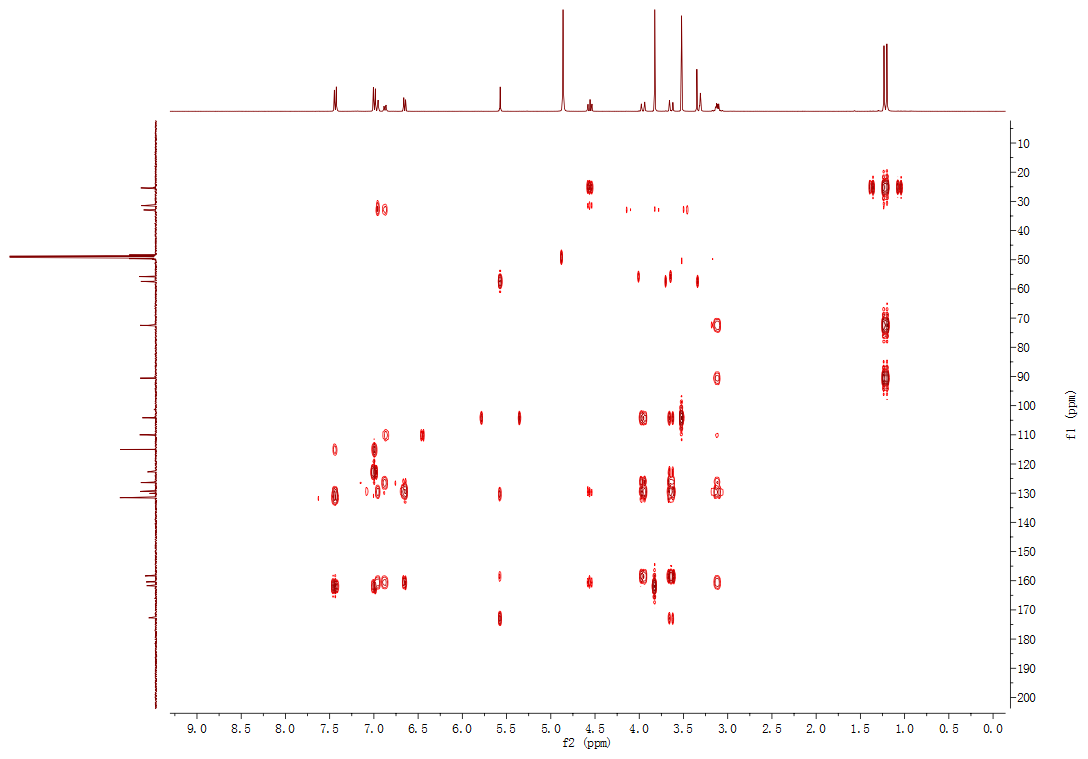
**

**Figure S28.** HMBC spectrum of compound **3** (Recorded in methanol-*d*_4_)

**
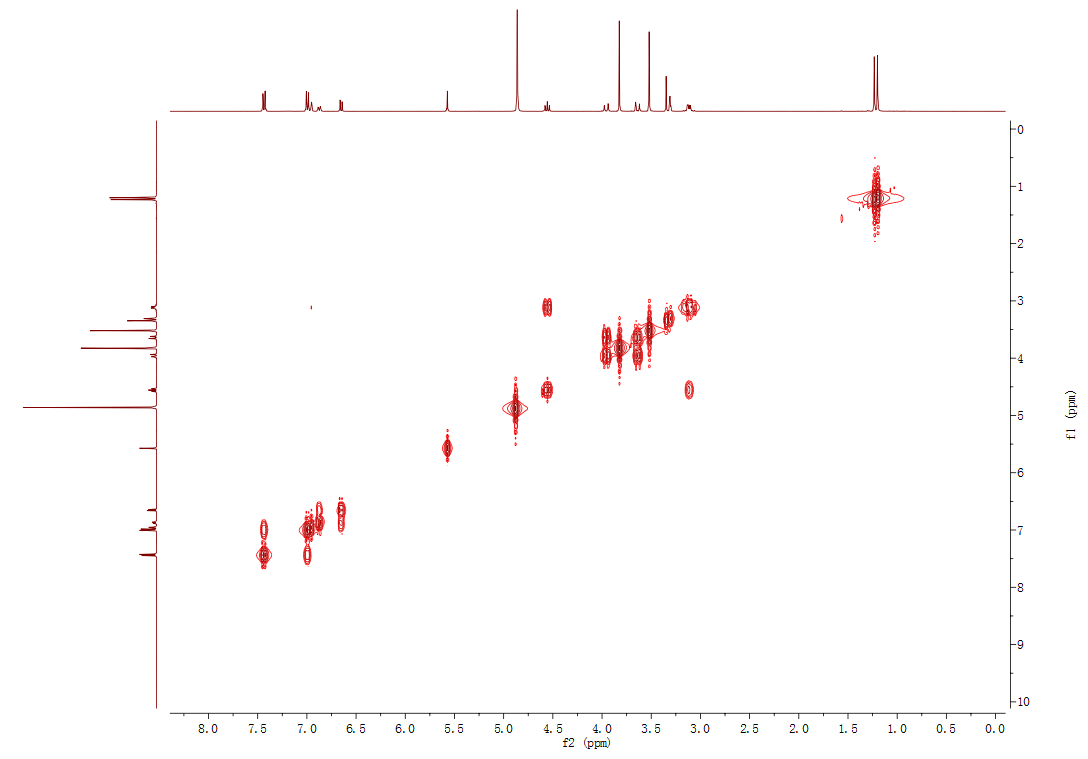
**

**Figure S29.** ^1^H–^1^H COSY spectrum of compound **3** (Recorded in methanol-*d*_4_)

**Figure S30.** HRESIMS spectrum of compound **3**

**Figure S31.** UV spectrum of compound **3**

**Figure S32.** IR spectrum of compound **3**


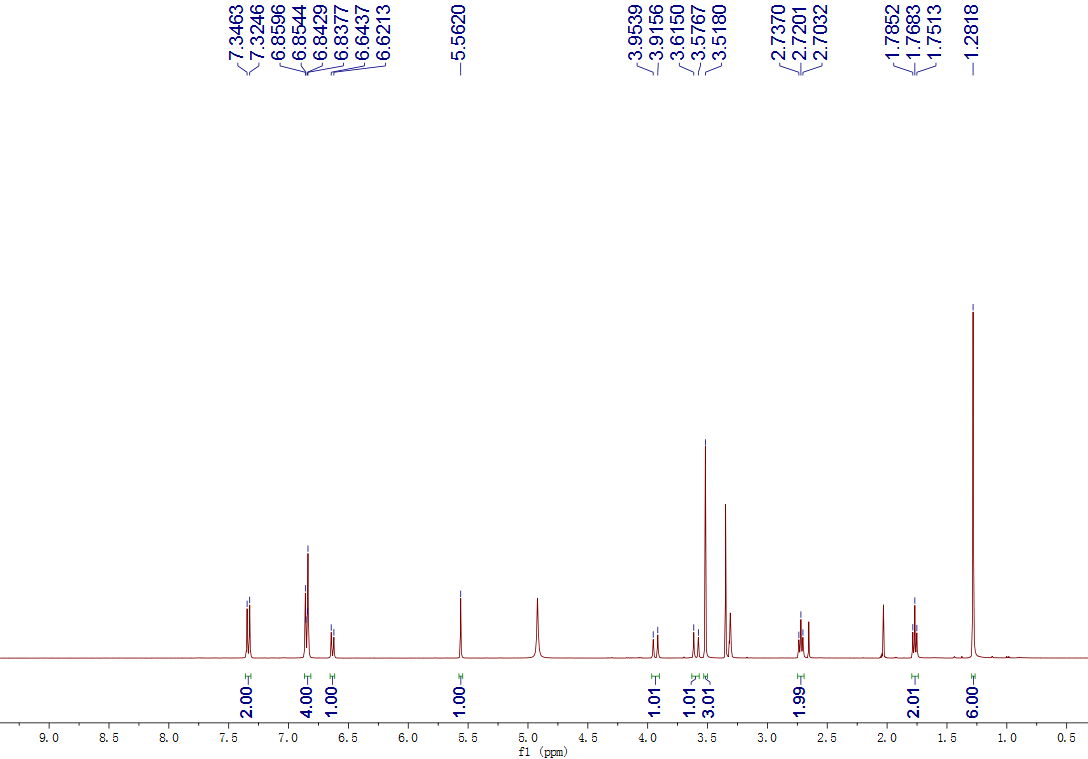


**Figure S33.** ^1^H NMR spectrum of compound **4** (Recorded in methanol-*d*_4_)


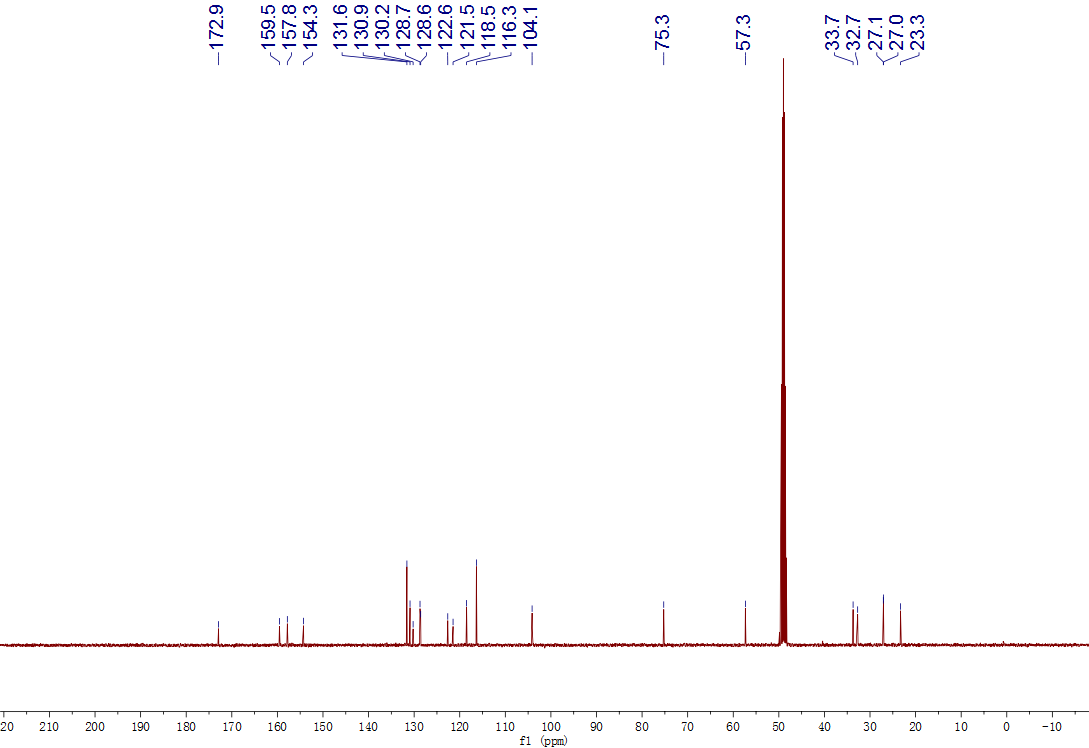


**Figure S34.** ^13^C NMR spectrum of compound **4** (Recorded in methanol-*d*_4_)

**
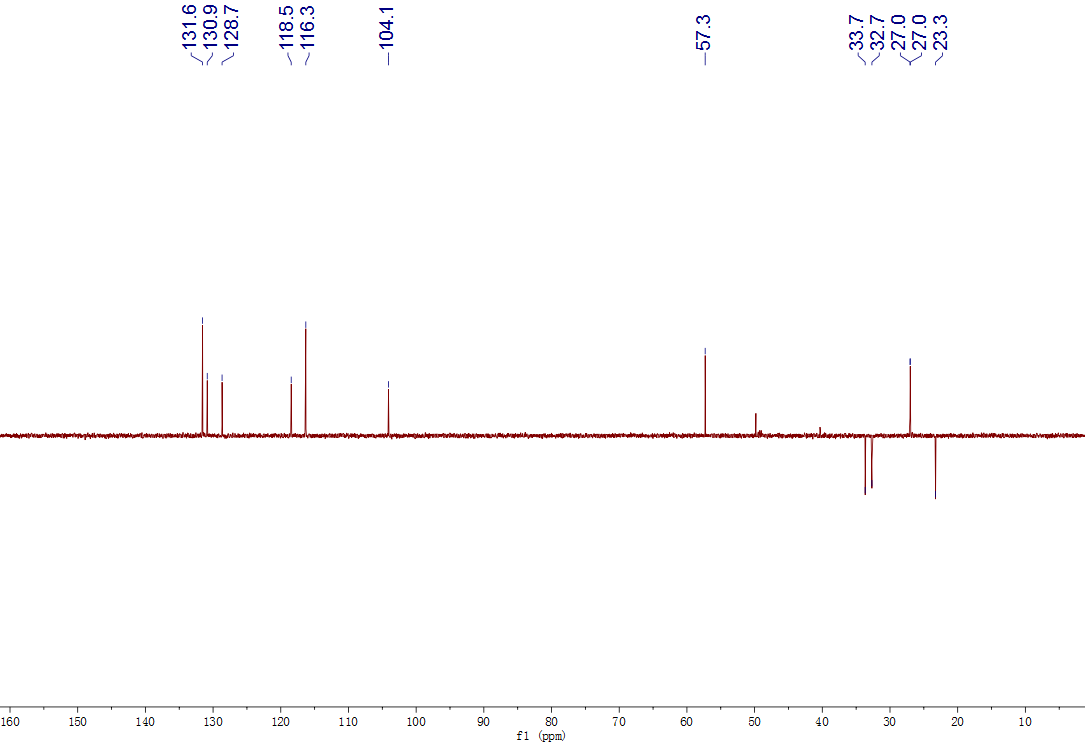
**

**Figure S35.** DEPT spectrum of compound **4** (Recorded in methanol-*d*_4_)

**
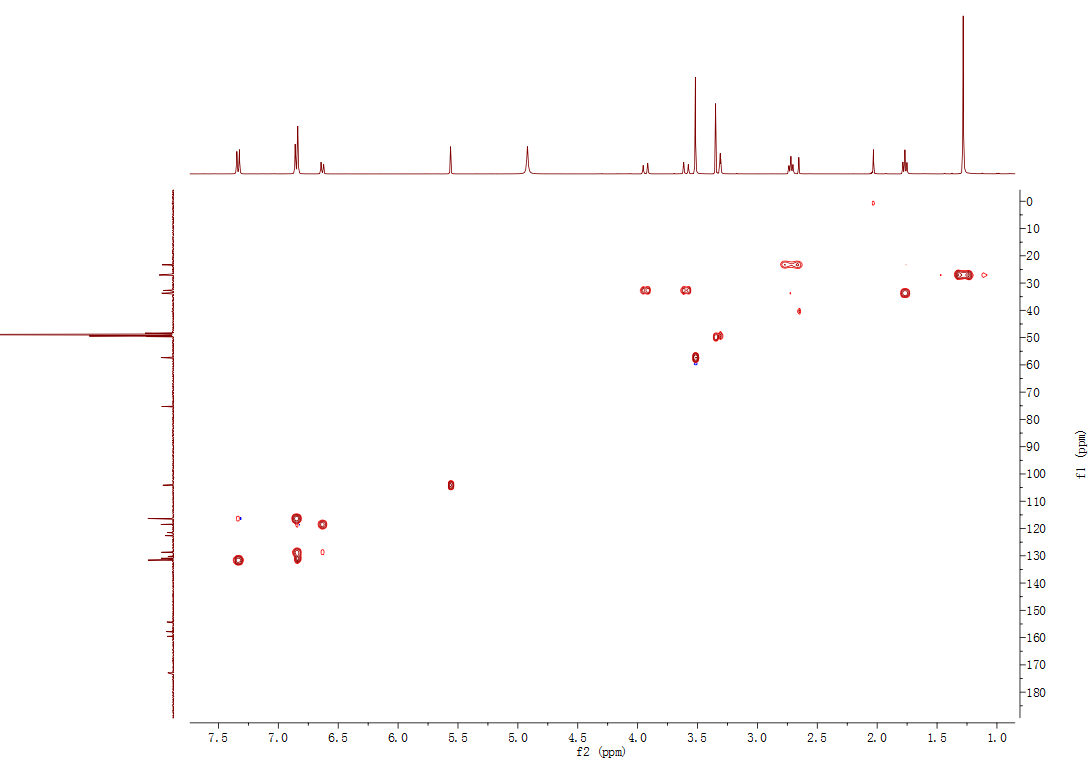
**

**Figure S36.** HSQC spectrum of compound **4** (Recorded in methanol-*d*_4_)

**
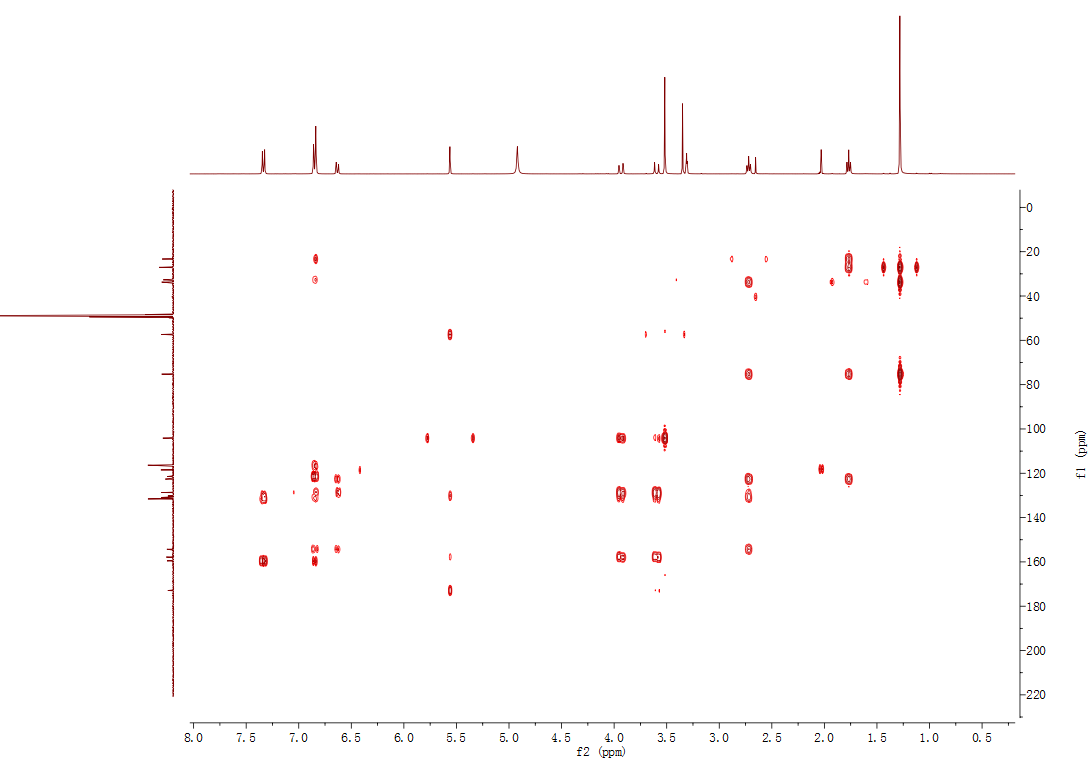
**

**Figure S37.** HMBC spectrum of compound **4** (Recorded in methanol-*d*_4_)

**
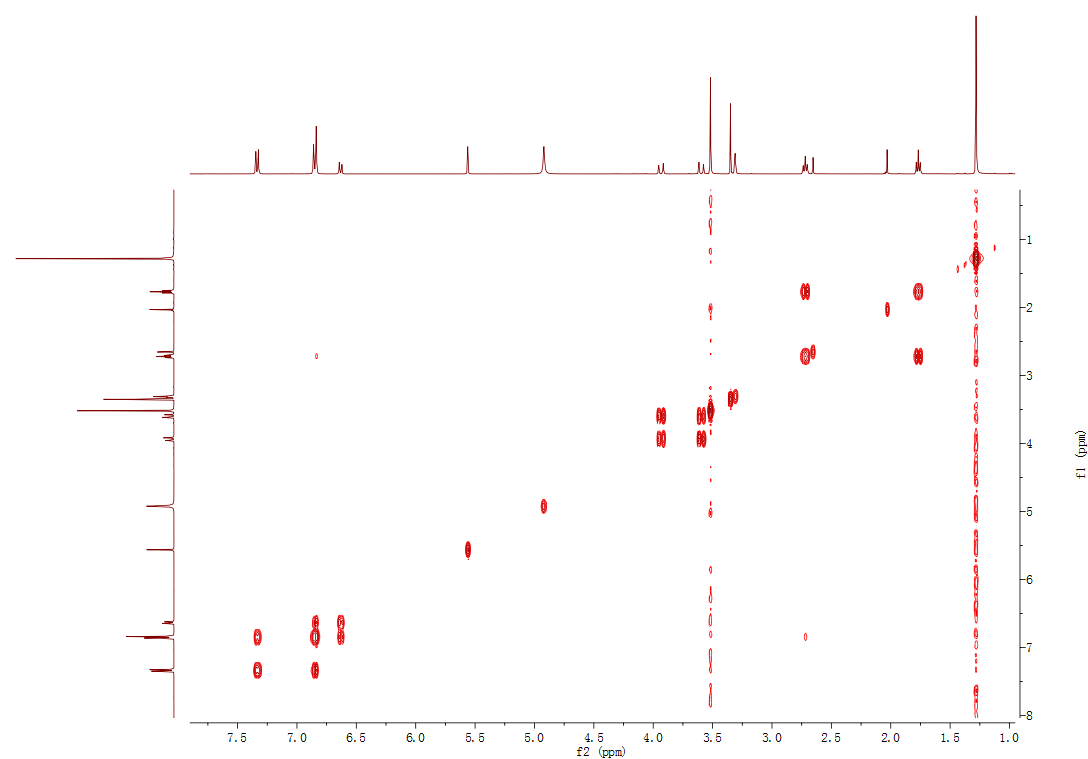
**

**Figure S38.** ^1^H–^1^H COSY spectrum of compound **4** (Recorded in methanol-*d*_4_)


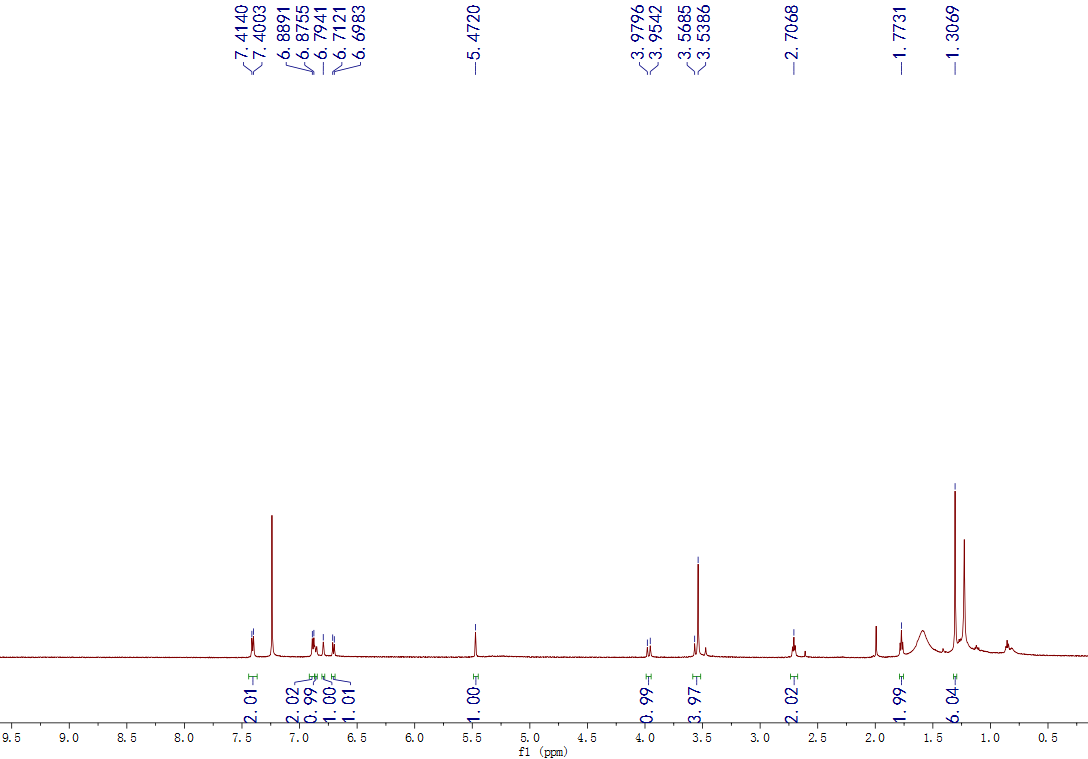


**Figure S39.** ^1^H NMR spectrum of compound **4** (Recorded in CDCl_3_)


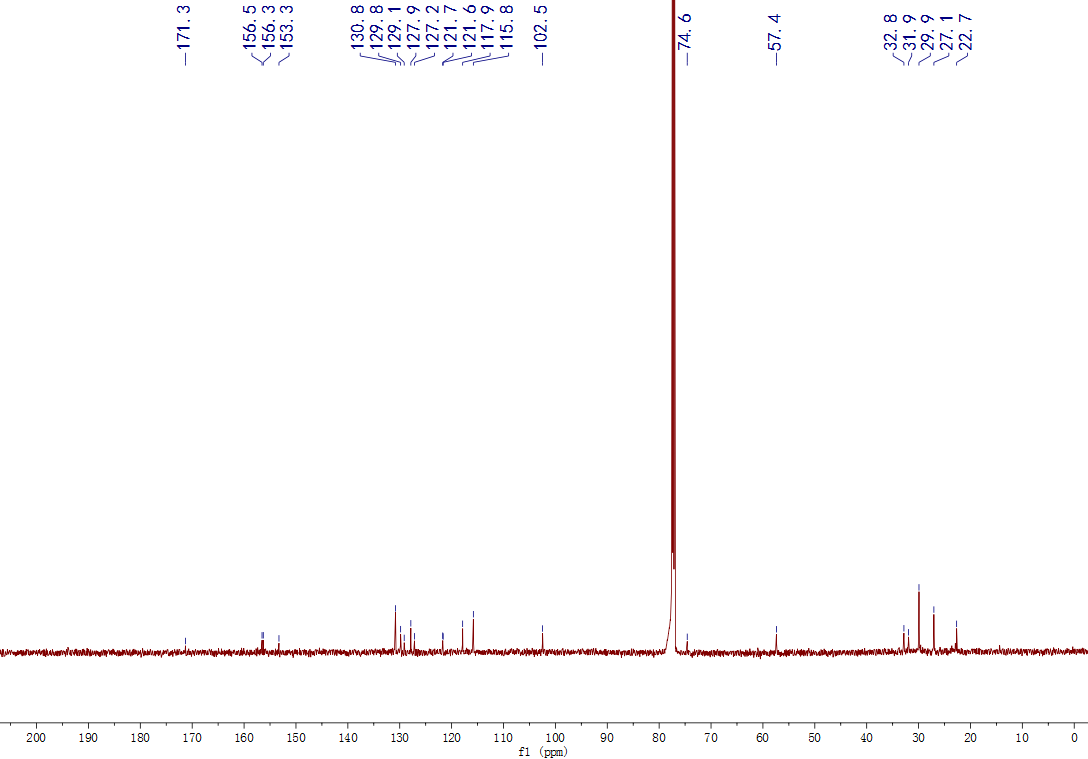


**Figure S40.** ^13^C NMR spectrum of compound **4** (Recorded in CDCl_3_)

**Figure S41.** HRESIMS spectrum of compound **4**

**Figure S42.** UV spectrum of compound **4**

**Figure S43.** IR spectrum of compound **4**


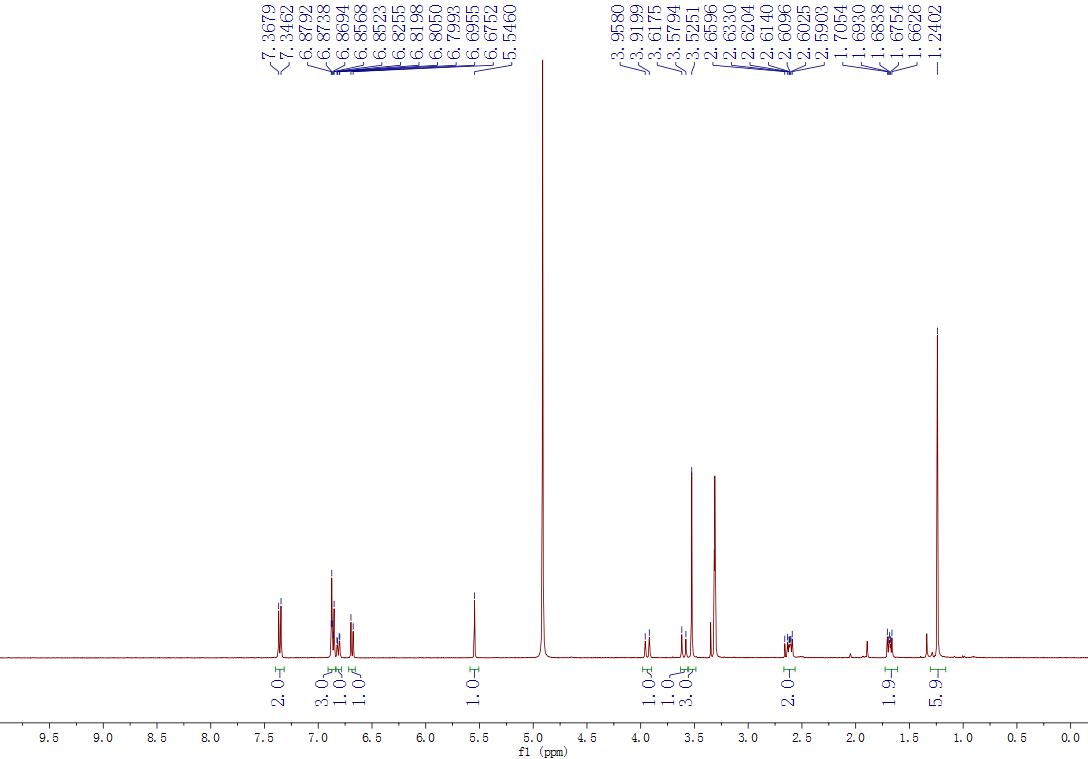


**Figure S44.** ^1^H NMR spectrum of compound **5** (Recorded in methanol-*d*_4_)


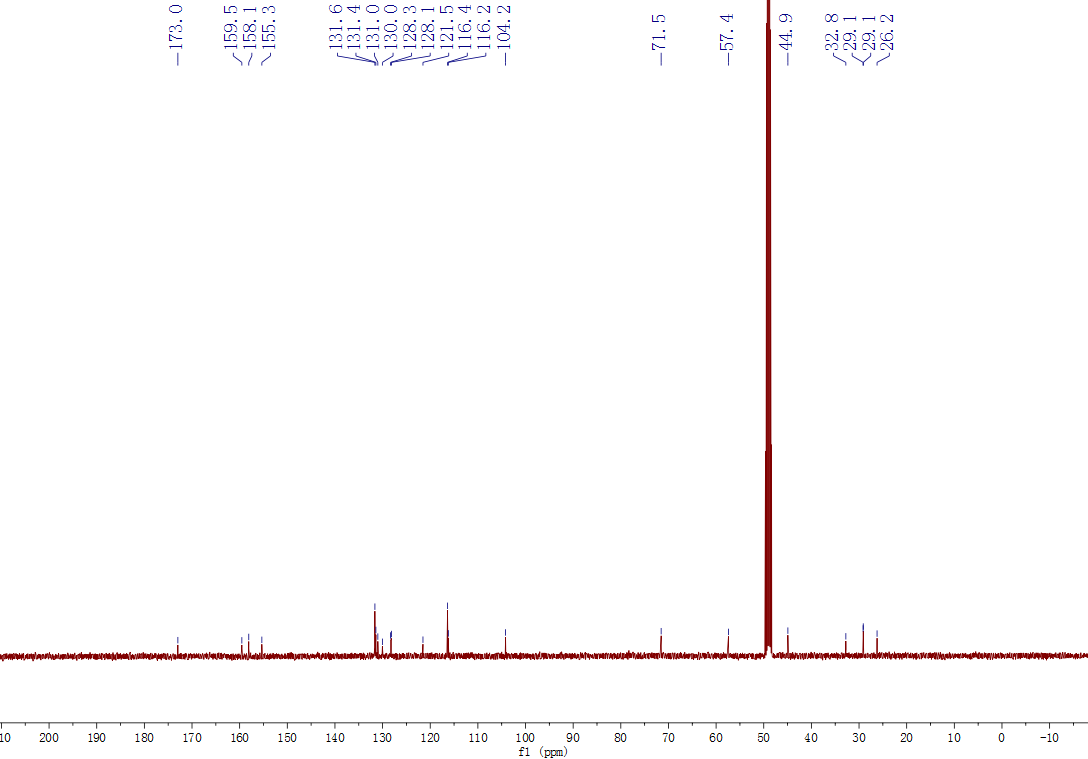


**Figure S45.** ^13^C NMR spectrum of compound **5** (Recorded in methanol-*d*_4_)

**
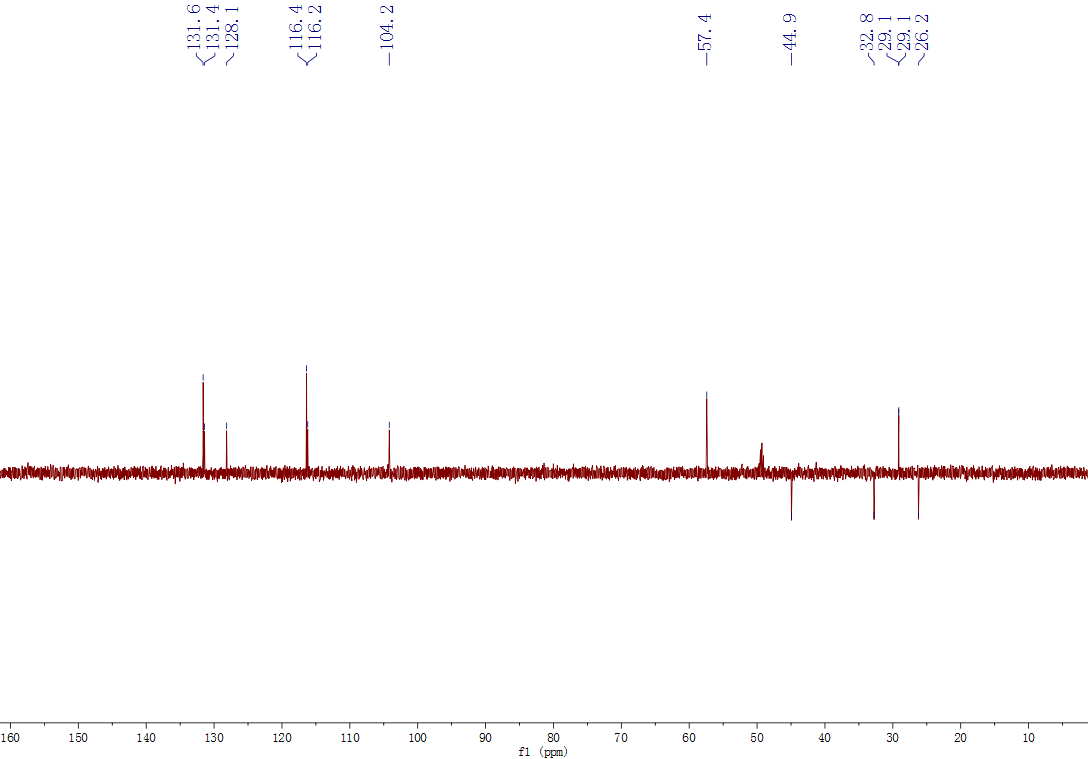
**

**Figure S46.** DEPT spectrum of compound **5** (Recorded in methanol-*d*_4_)

**
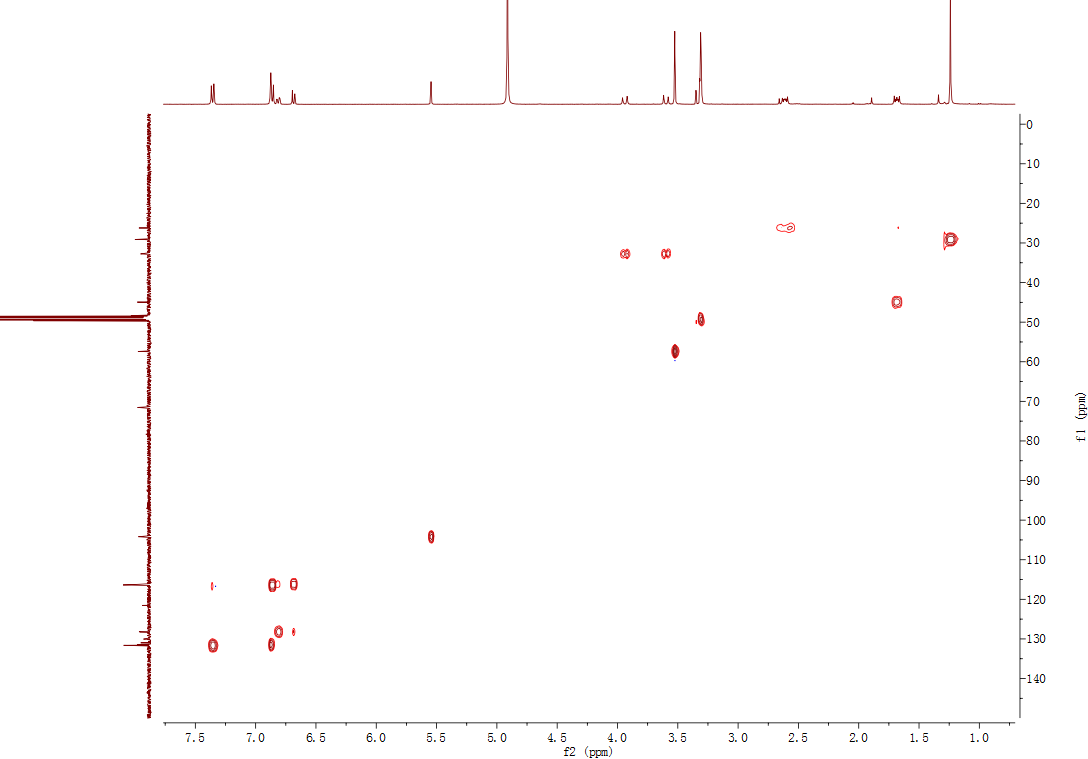
**

**Figure S47.** HSQC spectrum of compound **5** (Recorded in methanol-*d*_4_)

**
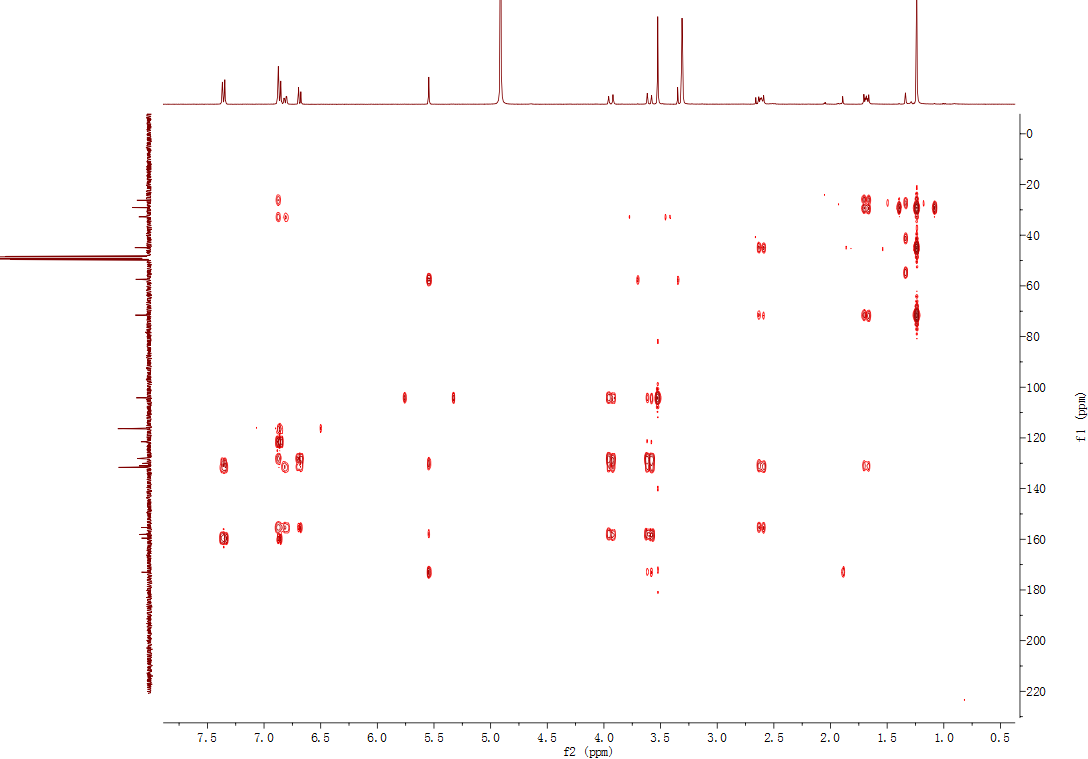
**

**Figure S48.** HMBC spectrum of compound **5** (Recorded in methanol-*d*_4_)

**
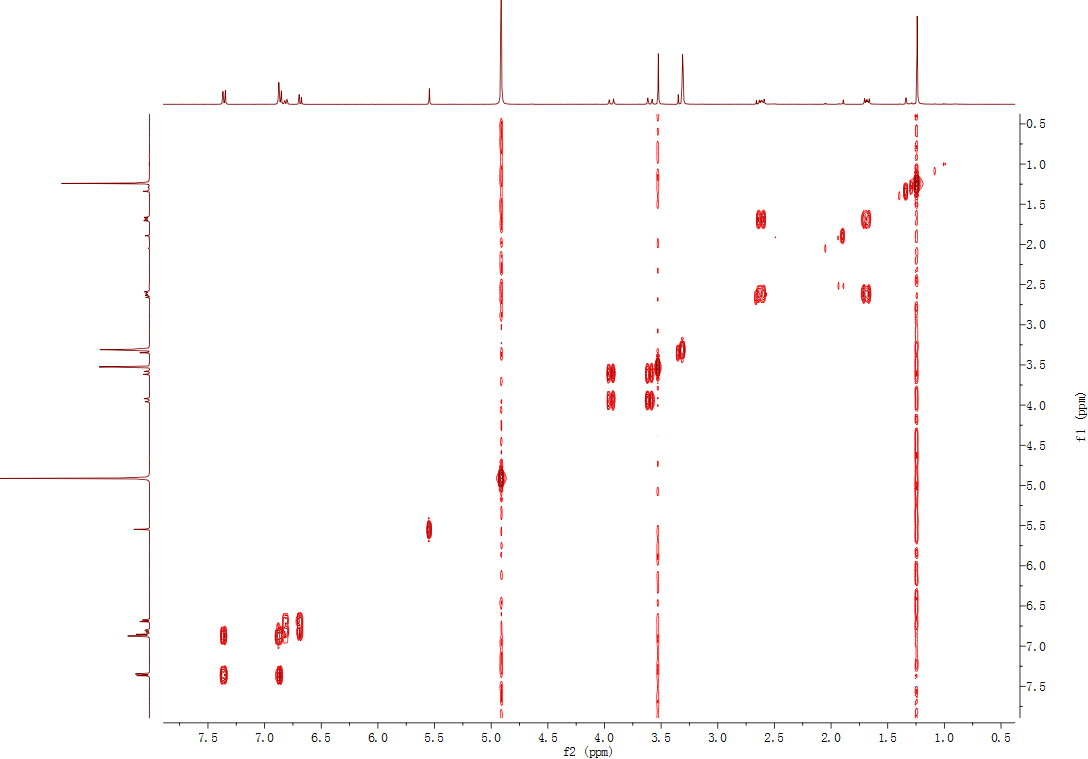
**

**Figure S49.** ^1^H–^1^H COSY spectrum of compound **5** (Recorded in methanol-*d*_4_)

**Figure S50.** HRESIMS spectrum of compound **5**

**Figure S51.** UV spectrum of compound **5**

**Figure S52.** IR spectrum of compound **5**

**Calculated ECD data of compounds 1 and 2**

**Table S1.** Important thermodynamic parameters (a.u.) and Boltzmann distributions of the optimized (2*S*,3*S*)-**1** at B3LYP/6-31G(d,p) level in the gas phase.

| Conformations | E+ZPE | Gibbs | Ratio |
| --- | --- | --- | --- |
| OME69 | -1343.872955 | -1344.650937 | 22.45% |
| OME3 | -1343.873032 | -1344.644978 | 10.14% |
| OME68 | -1343.873082 | -1344.645186 | 9.22% |
| OME60 | -1343.872851 | -1344.645299 | 9.03% |
| OME9 | -1343.873060 | -1344.645438 | 8.93% |
| OME21 | -1343.872799 | -1344.643987 | 8.84% |
| OME75 | -1343.87288 | -1344.646528 | 7.95% |
| OME48 | -1343.872691 | -1344.644975 | 7.87% |
| OME54 | -1343.872942 | -1344.648332 | 7.38% |

E+ZPE, G: total energy with zero point energy (ZPE) and Gibbs free energy in the gas phase at B3LYP/6-31G(d,p) level., %: Boltzmann distributions, using the relative Gibbs free energies as weighting factors.

**Table S2.** Optimized coordinate of (2*S*,3*S*)-**1** in the gas phase (Å) at B3LYP/6-31G(d,p) level.

| **OME69** | | | | **OME3** | | | |
| --- | --- | --- | --- | --- | --- | --- | --- |
| C1 | 4.529 | -0.251 | -0.496 | C1 | -4.148 | 1.287 | 0.794 |
| C2 | 3.375 | 1.508 | 0.672 | C2 | -4.286 | -0.704 | -0.549 |
| C3 | -1.773 | -2.434 | -0.751 | C3 | 1.544 | -2.349 | -0.652 |
| C4 | 5.419 | 0.677 | -1.034 | C4 | -5.275 | 1.805 | 0.161 |
| C5 | 4.259 | 2.447 | 0.142 | C5 | -5.415 | -0.199 | -1.191 |
| C6 | -3.09 | -2.277 | -1.186 | C6 | 2.652 | -2.488 | -1.487 |
| C7 | -2.177 | -0.849 | 1.005 | C7 | 2.307 | -0.162 | -0.022 |
| C8 | 3.493 | 0.147 | 0.36 | C8 | -3.633 | 0.029 | 0.451 |
| C9 | -1.293 | -1.722 | 0.353 | C9 | 1.351 | -1.181 | 0.095 |
| C10 | -3.498 | -0.662 | 0.591 | C10 | 3.429 | -0.27 | -0.847 |
| C11 | 5.285 | 2.033 | -0.715 | C11 | -5.914 | 1.06 | -0.837 |
| C12 | -3.947 | -1.393 | -0.525 | C12 | 3.59 | -1.456 | -1.585 |
| C13 | -4.436 | 1.676 | 0.637 | C13 | 5.7 | 0.55 | -0.119 |
| C14 | -5.481 | 2.341 | 0.119 | C14 | 6.194 | 1.234 | 0.926 |
| C15 | 2.515 | -0.783 | 2.439 | C15 | -2.716 | -1.873 | 1.748 |
| C16 | 1.088 | -0.603 | -1.15 | C16 | -0.951 | 0.679 | -0.56 |
| C17 | -5.291 | 3.707 | -0.498 | C17 | 7.457 | 0.774 | 1.616 |
| C18 | -6.903 | 1.837 | 0.094 | C18 | 5.587 | 2.485 | 1.514 |
| C19 | 1.658 | -1.708 | -3.164 | C19 | -0.503 | 2.994 | -0.353 |
| C20 | -4.416 | 0.317 | 1.303 | C20 | 4.459 | 0.846 | -0.933 |
| C21 | 0.138 | -1.872 | 0.828 | C21 | 0.133 | -1.017 | 0.982 |
| C22 | 2.512 | -0.885 | 0.919 | C22 | -2.384 | -0.512 | 1.147 |
| C23 | 1.068 | -0.716 | 0.369 | C23 | -1.153 | -0.622 | 0.206 |
| O1 | 1.778 | -0.076 | 3.1 | O1 | -2.575 | -2.943 | 1.188 |
| O2 | 0.696 | 0.358 | -1.782 | O2 | -0.996 | 0.791 | -1.768 |
| O3 | 6.183 | 2.902 | -1.267 | O3 | -7.017 | 1.61 | -1.425 |
| O4 | -5.248 | -1.213 | -0.915 | O4 | 4.695 | -1.551 | -2.392 |
| O5 | 3.482 | -1.546 | 2.987 | O5 | -3.241 | -1.757 | 2.984 |
| O6 | 1.591 | -1.714 | -1.723 | O6 | -0.703 | 1.71 | 0.273 |
| H1 | 4.643 | -1.301 | -0.75 | H1 | -3.66 | 1.875 | 1.568 |
| H2 | 2.591 | 1.843 | 1.345 | H2 | -3.918 | -1.688 | -0.826 |
| H3 | -1.12 | -3.127 | -1.275 | H3 | 0.826 | -3.163 | -0.581 |
| H4 | 6.219 | 0.364 | -1.697 | H4 | -5.67 | 2.779 | 0.43 |
| H5 | 4.152 | 3.499 | 0.397 | H5 | -5.91 | -0.783 | -1.964 |
| H6 | -3.455 | -2.844 | -2.04 | H6 | 2.792 | -3.402 | -2.061 |
| H7 | -1.826 | -0.296 | 1.875 | H7 | 2.174 | 0.754 | 0.552 |
| H8 | -3.456 | 2.155 | 0.582 | H8 | 6.238 | -0.342 | -0.439 |
| H9 | -5.605 | 3.711 | -1.552 | H9 | 8.232 | 1.553 | 1.585 |
| H10 | -5.91 | 4.46 | 0.011 | H10 | 7.272 | 0.566 | 2.68 |
| H11 | -4.248 | 4.037 | -0.453 | H11 | 7.867 | -0.133 | 1.159 |
| H12 | -7.558 | 2.5 | 0.678 | H12 | 5.355 | 2.336 | 2.578 |
| H13 | -7.294 | 1.844 | -0.933 | H13 | 6.3 | 3.32 | 1.469 |
| H14 | -7.011 | 0.823 | 0.484 | H14 | 4.669 | 2.802 | 1.014 |
| H15 | 2.083 | -2.672 | -3.44 | H15 | -0.319 | 3.691 | 0.465 |
| H16 | 2.3 | -0.893 | -3.509 | H16 | -1.394 | 3.286 | -0.913 |
| H17 | 0.659 | -1.592 | -3.59 | H17 | 0.357 | 2.957 | -1.026 |
| H18 | -5.424 | -0.102 | 1.369 | H18 | 4.75 | 0.977 | -1.984 |
| H19 | -4.053 | 0.435 | 2.334 | H19 | 3.992 | 1.782 | -0.615 |
| H20 | 0.16 | -1.897 | 1.923 | H20 | -0.07 | -1.96 | 1.5 |
| H21 | 0.553 | -2.821 | 0.473 | H21 | 0.327 | -0.26 | 1.749 |
| H22 | 2.878 | -1.88 | 0.652 | H22 | -2.137 | 0.162 | 1.972 |
| H23 | 0.664 | 0.231 | 0.735 | H23 | -1.359 | -1.385 | -0.55 |
| H24 | 5.983 | 3.805 | -0.969 | H24 | -7.368 | 0.996 | -2.091 |
| H25 | -5.433 | -1.788 | -1.676 | H25 | 4.681 | -2.405 | -2.854 |
| H26 | 3.462 | -1.392 | 3.952 | H26 | -3.478 | -2.656 | 3.285 |
| **OME68** | | | | **OME60** | | | |
| C1 | -4.603 | 0.083 | 0.597 | C1 | 4.129 | 1.068 | -0.969 |
| C2 | -3.5 | -1.372 | -0.97 | C2 | 4.017 | 0.031 | 1.2 |
| C3 | 1.694 | 2.197 | 1.082 | C3 | -1.505 | -2.42 | 0.949 |
| C4 | -5.736 | 0.111 | -0.212 | C4 | 4.992 | 2.04 | -0.47 |
| C5 | -4.627 | -1.354 | -1.789 | C5 | 4.882 | 0.995 | 1.712 |
| C6 | 2.815 | 2.796 | 0.505 | C6 | -2.791 | -2.341 | 1.482 |
| C7 | 2.283 | 0.117 | 0.039 | C7 | -2.19 | -1.024 | -0.879 |
| C8 | -3.467 | -0.654 | 0.233 | C8 | 3.624 | 0.052 | -0.146 |
| C9 | 1.408 | 0.846 | 0.859 | C9 | -1.181 | -1.76 | -0.243 |
| C10 | 3.411 | 0.686 | -0.556 | C10 | -3.488 | -0.921 | -0.37 |
| C11 | -5.751 | -0.609 | -1.412 | C11 | 5.372 | 2.007 | 0.877 |
| C12 | 3.669 | 2.047 | -0.308 | C12 | -3.777 | -1.592 | 0.833 |
| C13 | 5.59 | -0.577 | -0.679 | C13 | -4.676 | 1.298 | -0.498 |
| C14 | 6.025 | -1.825 | -0.447 | C14 | -5.761 | 1.896 | 0.02 |
| C15 | -1.872 | -2.093 | 1.468 | C15 | 3.219 | -2.377 | -0.43 |
| C16 | -1.419 | 1.431 | -0.06 | C16 | 0.736 | 0.581 | -0.213 |
| C17 | 7.302 | -2.06 | 0.328 | C17 | -5.682 | 3.314 | 0.537 |
| C18 | 5.34 | -3.089 | -0.91 | C18 | -7.126 | 1.263 | 0.141 |
| C19 | -2.293 | 3.551 | 0.526 | C19 | 0.228 | 2.358 | -1.693 |
| C20 | 4.348 | -0.138 | -1.425 | C20 | -4.55 | -0.095 | -1.075 |
| C21 | 0.184 | 0.188 | 1.461 | C21 | 0.221 | -1.827 | -0.814 |
| C22 | -2.224 | -0.651 | 1.124 | C22 | 2.653 | -0.989 | -0.705 |
| C23 | -1.008 | 0.065 | 0.473 | C23 | 1.22 | -0.86 | -0.12 |
| O1 | -1.119 | -2.805 | 0.832 | O1 | 2.99 | -3.048 | 0.558 |
| O2 | -1.35 | 1.775 | -1.222 | O2 | 0.434 | 1.278 | 0.735 |
| O3 | -6.886 | -0.549 | -2.17 | O3 | 6.221 | 2.983 | 1.317 |
| O4 | 4.783 | 2.591 | -0.894 | O4 | -5.053 | -1.492 | 1.322 |
| O5 | -2.538 | -2.521 | 2.558 | O5 | 4.06 | -2.774 | -1.405 |
| O6 | -1.868 | 2.233 | 0.926 | O6 | 0.671 | 1 | -1.493 |
| H1 | -4.601 | 0.647 | 1.526 | H1 | 3.84 | 1.103 | -2.016 |
| H2 | -2.64 | -1.963 | -1.272 | H2 | 3.653 | -0.754 | 1.857 |
| H3 | 1.041 | 2.791 | 1.716 | H3 | -0.751 | -3.011 | 1.464 |
| H4 | -6.613 | 0.684 | 0.074 | H4 | 5.38 | 2.826 | -1.11 |
| H5 | -4.636 | -1.918 | -2.719 | H5 | 5.178 | 0.962 | 2.758 |
| H6 | 3.028 | 3.847 | 0.688 | H6 | -3.033 | -2.865 | 2.404 |
| H7 | 2.08 | -0.937 | -0.145 | H7 | -1.962 | -0.515 | -1.814 |
| H8 | 6.186 | 0.247 | -0.287 | H8 | -3.748 | 1.872 | -0.52 |
| H9 | 7.112 | -2.665 | 1.226 | H9 | -6.388 | 3.968 | 0.006 |
| H10 | 8.032 | -2.62 | -0.273 | H10 | -5.957 | 3.363 | 1.6 |
| H11 | 7.769 | -1.121 | 0.642 | H11 | -4.678 | 3.736 | 0.425 |
| H12 | 6.004 | -3.669 | -1.566 | H12 | -7.478 | 1.307 | 1.181 |
| H13 | 5.11 | -3.738 | -0.053 | H13 | -7.863 | 1.817 | -0.458 |
| H14 | 4.409 | -2.908 | -1.452 | H14 | -7.148 | 0.219 | -0.175 |
| H15 | -2.612 | 4.045 | 1.443 | H15 | 0.245 | 2.514 | -2.771 |
| H16 | -3.122 | 3.483 | -0.182 | H16 | 0.905 | 3.055 | -1.193 |
| H17 | -1.464 | 4.094 | 0.067 | H17 | -0.784 | 2.488 | -1.303 |
| H18 | 3.799 | -0.997 | -1.819 | H18 | -4.268 | -0.014 | -2.134 |
| H19 | 4.651 | 0.469 | -2.289 | H19 | -5.508 | -0.62 | -1.043 |
| H20 | 0.439 | -0.823 | 1.798 | H20 | 0.614 | -2.843 | -0.703 |
| H21 | -0.146 | 0.749 | 2.343 | H21 | 0.2 | -1.602 | -1.886 |
| H22 | -2.477 | -0.139 | 2.056 | H22 | 2.606 | -0.86 | -1.79 |
| H23 | -0.688 | -0.513 | -0.398 | H23 | 1.257 | -1.095 | 0.946 |
| H24 | -6.768 | -1.088 | -2.969 | H24 | 6.401 | 2.852 | 2.262 |
| H25 | 4.843 | 3.53 | -0.653 | H25 | -5.12 | -2.014 | 2.138 |
| H26 | -2.308 | -3.462 | 2.689 | H26 | 4.425 | -3.641 | -1.141 |
| **OME9** | | | | **OME21** | | | |
| C1 | 4.36 | -0.486 | -0.204 | C1 | -4.38 | -0.894 | -1.087 |
| C2 | 3.177 | 1.583 | 0.124 | C2 | -4.351 | 0.429 | 0.923 |
| C3 | -1.903 | -2.685 | -0.017 | C3 | 1.536 | 1.858 | 1.174 |
| C4 | 5.297 | 0.164 | -1.003 | C4 | -5.469 | -1.617 | -0.606 |
| C5 | 4.108 | 2.248 | -0.672 | C5 | -5.441 | -0.286 | 1.416 |
| C6 | -3.184 | -2.739 | -0.568 | C6 | 2.712 | 1.645 | 1.892 |
| C7 | -2.441 | -0.578 | 1.001 | C7 | 2.158 | 0.106 | -0.344 |
| C8 | 3.286 | 0.208 | 0.37 | C8 | -3.801 | 0.137 | -0.332 |
| C9 | -1.509 | -1.602 | 0.776 | C9 | 1.237 | 1.09 | 0.043 |
| C10 | -3.73 | -0.601 | 0.465 | C10 | 3.349 | -0.129 | 0.35 |
| C11 | 5.173 | 1.538 | -1.241 | C11 | -6.004 | -1.315 | 0.651 |
| C12 | -4.091 | -1.703 | -0.333 | C12 | 3.617 | 0.66 | 1.483 |
| C13 | -4.823 | 1.479 | -0.451 | C13 | 5.455 | -0.596 | -0.958 |
| C14 | -4.563 | 2.796 | -0.483 | C14 | 6.779 | -0.636 | -0.738 |
| C15 | 2.155 | 0.156 | 2.576 | C15 | -2.919 | 2.389 | -0.871 |
| C16 | 0.983 | -1.114 | -0.883 | C16 | -1.075 | -0.863 | 0.115 |
| C17 | -4.727 | 3.579 | -1.765 | C17 | 7.738 | 0.005 | -1.716 |
| C18 | -4.103 | 3.622 | 0.694 | C18 | 7.443 | -1.294 | 0.446 |
| C19 | 1.686 | -2.91 | -2.255 | C19 | -0.726 | -2.913 | -1.016 |
| C20 | -4.715 | 0.528 | 0.721 | C20 | 4.34 | -1.182 | -0.118 |
| C21 | -0.116 | -1.522 | 1.366 | C21 | -0.053 | 1.3 | -0.724 |
| C22 | 2.255 | -0.535 | 1.222 | C22 | -2.59 | 0.901 | -0.869 |
| C23 | 0.855 | -0.622 | 0.553 | C23 | -1.29 | 0.634 | -0.061 |
| O1 | 1.379 | 1.05 | 2.855 | O1 | -2.704 | 3.159 | 0.045 |
| O2 | 0.65 | -0.486 | -1.867 | O2 | -1.031 | -1.44 | 1.183 |
| O3 | 6.118 | 2.129 | -2.03 | O3 | -7.071 | -2.053 | 1.076 |
| O4 | -5.361 | -1.712 | -0.852 | O4 | 4.783 | 0.419 | 2.161 |
| O5 | 3.073 | -0.313 | 3.444 | O5 | -3.531 | 2.762 | -2.012 |
| O6 | 1.514 | -2.352 | -0.936 | O6 | -0.927 | -1.486 | -1.071 |
| H1 | 4.465 | -1.554 | -0.027 | H1 | -3.972 | -1.138 | -2.064 |
| H2 | 2.362 | 2.149 | 0.567 | H2 | -3.932 | 1.231 | 1.524 |
| H3 | -1.209 | -3.5 | -0.204 | H3 | 0.847 | 2.633 | 1.502 |
| H4 | 6.127 | -0.378 | -1.446 | H4 | -5.913 | -2.415 | -1.194 |
| H5 | 4.009 | 3.316 | -0.851 | H5 | -5.856 | -0.044 | 2.392 |
| H6 | -3.482 | -3.59 | -1.177 | H6 | 2.933 | 2.247 | 2.77 |
| H7 | -2.157 | 0.268 | 1.625 | H7 | 1.943 | -0.503 | -1.22 |
| H8 | -5.149 | 1.005 | -1.377 | H8 | 5.108 | -0.079 | -1.854 |
| H9 | -5.455 | 4.393 | -1.641 | H9 | 8.341 | 0.781 | -1.224 |
| H10 | -3.781 | 4.053 | -2.061 | H10 | 8.449 | -0.733 | -2.113 |
| H11 | -5.064 | 2.945 | -2.592 | H11 | 7.215 | 0.464 | -2.561 |
| H12 | -3.144 | 4.11 | 0.47 | H12 | 8.091 | -2.12 | 0.117 |
| H13 | -4.819 | 4.43 | 0.901 | H13 | 8.096 | -0.579 | 0.965 |
| H14 | -3.978 | 3.044 | 1.612 | H14 | 6.734 | -1.688 | 1.176 |
| H15 | 2.117 | -3.898 | -2.1 | H15 | -0.628 | -3.234 | -2.053 |
| H16 | 2.361 | -2.285 | -2.844 | H16 | -1.584 | -3.398 | -0.544 |
| H17 | 0.722 | -2.987 | -2.763 | H17 | 0.181 | -3.147 | -0.454 |
| H18 | -5.706 | 0.095 | 0.916 | H18 | 4.741 | -1.72 | 0.745 |
| H19 | -4.417 | 1.057 | 1.631 | H19 | 3.792 | -1.917 | -0.724 |
| H20 | -0.171 | -1.113 | 2.381 | H20 | -0.26 | 2.373 | -0.803 |
| H21 | 0.317 | -2.525 | 1.443 | H21 | 0.05 | 0.914 | -1.744 |
| H22 | 2.627 | -1.549 | 1.393 | H22 | -2.43 | 0.595 | -1.907 |
| H23 | 0.436 | 0.386 | 0.489 | H23 | -1.415 | 1.04 | 0.946 |
| H24 | 5.92 | 3.076 | -2.115 | H24 | -7.342 | -1.749 | 1.958 |
| H25 | -5.486 | -2.522 | -1.373 | H25 | 4.833 | 1.015 | 2.926 |
| H26 | 2.988 | 0.207 | 4.267 | H26 | -3.759 | 3.709 | -1.924 |
| **OME75** | | | | **OME48** | | | |
| C1 | 3.838 | 1.194 | -0.894 | C1 | -3.772 | 1.358 | 0.659 |
| C2 | 3.923 | -0.186 | 1.076 | C2 | -4.017 | -0.568 | -0.764 |
| C3 | -1.592 | -2.72 | 0.802 | C3 | 1.613 | -2.825 | -0.43 |
| C4 | 4.728 | 2.094 | -0.313 | C4 | -4.77 | 2.021 | -0.052 |
| C5 | 4.814 | 0.706 | 1.669 | C5 | -5.018 | 0.083 | -1.482 |
| C6 | -2.838 | -2.753 | 1.424 | C6 | 2.769 | -3.059 | -1.175 |
| C7 | -2.406 | -1.048 | -0.717 | C7 | 2.525 | -0.729 | 0.307 |
| C8 | 3.419 | 0.043 | -0.212 | C8 | -3.376 | 0.058 | 0.315 |
| C9 | -1.352 | -1.862 | -0.28 | C9 | 1.471 | -1.653 | 0.321 |
| C10 | -3.668 | -1.058 | -0.116 | C10 | 3.698 | -0.933 | -0.425 |
| C11 | 5.22 | 1.852 | 0.974 | C11 | -5.396 | 1.384 | -1.129 |
| C12 | -3.87 | -1.924 | 0.973 | C12 | 3.803 | -2.118 | -1.177 |
| C13 | -4.91 | 1.107 | 0.225 | C13 | 4.848 | 1.039 | 0.712 |
| C14 | -4.831 | 2.386 | -0.176 | C14 | 4.743 | 2.378 | 0.687 |
| C15 | 3.039 | -2.316 | -0.874 | C15 | -2.773 | -1.984 | 1.586 |
| C16 | 0.525 | 0.47 | 0.058 | C16 | -0.57 | 0.471 | -0.431 |
| C17 | -4.987 | 3.513 | 0.82 | C17 | 4.811 | 3.179 | 1.966 |
| C18 | -4.599 | 2.841 | -1.597 | C18 | 4.559 | 3.211 | -0.559 |
| C19 | -0.164 | 2.462 | -1.019 | C19 | 0.062 | 2.723 | -0.072 |
| C20 | -4.783 | -0.15 | -0.608 | C20 | 4.839 | 0.076 | -0.447 |
| C21 | 0.01 | -1.802 | -0.94 | C21 | 0.206 | -1.386 | 1.111 |
| C22 | 2.425 | -0.921 | -0.862 | C22 | -2.256 | -0.64 | 1.088 |
| C23 | 1.042 | -0.946 | -0.155 | C23 | -0.963 | -0.834 | 0.249 |
| O1 | 2.91 | -3.15 | 0.002 | O1 | -2.687 | -3.038 | 0.986 |
| O2 | 0.292 | 0.971 | 1.14 | O2 | -0.488 | 0.634 | -1.632 |
| O3 | 6.092 | 2.764 | 1.498 | O3 | -6.367 | 2.077 | -1.794 |
| O4 | -5.112 | -1.921 | 1.557 | O4 | 4.953 | -2.303 | -1.902 |
| O5 | 3.801 | -2.516 | -1.967 | O5 | -3.393 | -1.869 | 2.777 |
| O6 | 0.34 | 1.113 | -1.112 | O6 | -0.31 | 1.44 | 0.469 |
| H1 | 3.462 | 1.392 | -1.894 | H1 | -3.291 | 1.862 | 1.492 |
| H2 | 3.625 | -1.076 | 1.622 | H2 | -3.74 | -1.581 | -1.044 |
| H3 | -0.8 | -3.373 | 1.161 | H3 | 0.819 | -3.568 | -0.433 |
| H4 | 5.05 | 2.984 | -0.845 | H4 | -5.072 | 3.028 | 0.217 |
| H5 | 5.196 | 0.511 | 2.669 | H5 | -5.506 | -0.419 | -2.314 |
| H6 | -3.014 | -3.427 | 2.26 | H6 | 2.872 | -3.974 | -1.754 |
| H7 | -2.242 | -0.382 | -1.562 | H7 | 2.438 | 0.185 | 0.89 |
| H8 | -5.086 | 0.92 | 1.285 | H8 | 4.97 | 0.565 | 1.687 |
| H9 | -4.096 | 4.156 | 0.832 | H9 | 5.643 | 3.897 | 1.938 |
| H10 | -5.833 | 4.162 | 0.552 | H10 | 3.897 | 3.772 | 2.11 |
| H11 | -5.152 | 3.141 | 1.836 | H11 | 4.945 | 2.539 | 2.844 |
| H12 | -4.48 | 2.017 | -2.305 | H12 | 3.65 | 3.823 | -0.48 |
| H13 | -5.436 | 3.463 | -1.943 | H13 | 5.394 | 3.914 | -0.681 |
| H14 | -3.701 | 3.47 | -1.659 | H14 | 4.485 | 2.618 | -1.473 |
| H15 | -0.242 | 2.815 | -2.046 | H15 | 0.233 | 3.366 | 0.791 |
| H16 | 0.528 | 3.087 | -0.449 | H16 | -0.744 | 3.12 | -0.693 |
| H17 | -1.144 | 2.466 | -0.536 | H17 | 0.973 | 2.634 | -0.67 |
| H18 | -4.606 | 0.091 | -1.66 | H18 | 5.784 | -0.485 | -0.448 |
| H19 | -5.732 | -0.702 | -0.564 | H19 | 4.825 | 0.609 | -1.406 |
| H20 | 0.421 | -2.813 | -1.031 | H20 | -0.139 | -2.317 | 1.574 |
| H21 | -0.084 | -1.397 | -1.954 | H21 | 0.409 | -0.677 | 1.921 |
| H22 | 2.284 | -0.612 | -1.901 | H22 | -2.02 | -0.031 | 1.965 |
| H23 | 1.168 | -1.372 | 0.844 | H23 | -1.173 | -1.541 | -0.558 |
| H24 | 6.357 | 2.482 | 2.389 | H24 | -6.712 | 1.531 | -2.52 |
| H25 | -5.124 | -2.567 | 2.282 | H25 | 4.907 | -3.159 | -2.358 |
| H26 | 4.203 | -3.402 | -1.884 | H26 | -3.743 | -2.751 | 3.01 |
| **OME54** | | | | | | | |
| C1 | -4.746 | 0.27 | 0.477 | O6 | -1.833 | 2.111 | 1.048 |
| C2 | -3.727 | -1.223 | -1.111 | H1 | -4.73 | 0.789 | 1.432 |
| C3 | 1.693 | 1.718 | 1.386 | H2 | -2.919 | -1.888 | -1.404 |
| C4 | -5.829 | 0.455 | -0.378 | H3 | 1.081 | 2.32 | 2.052 |
| C5 | -4.806 | -1.049 | -1.976 | H4 | -6.654 | 1.104 | -0.102 |
| C6 | 2.886 | 2.248 | 0.889 | H5 | -4.828 | -1.569 | -2.931 |
| C7 | 2.107 | -0.313 | 0.176 | H6 | 3.195 | 3.253 | 1.168 |
| C8 | -3.677 | -0.566 | 0.126 | H7 | 1.806 | -1.321 | -0.106 |
| C9 | 1.282 | 0.428 | 1.037 | H8 | 4.983 | -1.999 | 0.306 |
| C10 | 3.307 | 0.185 | -0.336 | H9 | 8.264 | -2.613 | -0.428 |
| C11 | -5.862 | -0.205 | -1.612 | H10 | 8.284 | -1.293 | 0.735 |
| C12 | 3.689 | 1.489 | 0.035 | H11 | 7.105 | -2.609 | 0.917 |
| C13 | 5.319 | -1.328 | -0.487 | H12 | 8 | 0.376 | -1.23 |
| C14 | 6.642 | -1.2 | -0.672 | H13 | 7.879 | -0.918 | -2.416 |
| C15 | -2.286 | -2.217 | 1.345 | H14 | 6.578 | 0.281 | -2.278 |
| C16 | -1.425 | 1.32 | 0.036 | H15 | -2.419 | 3.959 | 1.637 |
| C17 | 7.616 | -1.973 | 0.188 | H16 | -2.89 | 3.545 | -0.043 |
| C18 | 7.292 | -0.316 | -1.708 | H17 | -1.194 | 3.964 | 0.326 |
| C19 | -2.099 | 3.486 | 0.709 | H18 | 3.563 | -1.435 | -1.699 |
| C20 | 4.19 | -0.658 | -1.24 | H19 | 4.577 | -0.042 | -2.057 |
| C21 | -0.022 | -0.151 | 1.548 | H20 | 0.128 | -1.199 | 1.83 |
| C22 | -2.482 | -0.731 | 1.066 | H21 | -0.34 | 0.385 | 2.448 |
| C23 | -1.174 | -0.107 | 0.507 | H22 | -2.727 | -0.245 | 2.015 |
| O1 | -1.577 | -2.967 | 0.702 | H23 | -0.872 | -0.667 | -0.382 |
| O2 | -1.273 | 1.715 | -1.102 | H24 | -6.845 | -0.495 | -3.236 |
| O3 | -6.947 | 0.01 | -2.413 | H25 | 4.997 | 2.881 | -0.165 |
| O4 | 4.864 | 1.971 | -0.479 | H26 | -2.908 | -3.597 | 2.47 |
| O5 | -3.04 | -2.632 | 2.382 |  |  |  |  |

**Table S3.** Important thermodynamic parameters (a.u.) and Boltzmann distributions of the optimized (2*R*,3*R*)-**1** at B3LYP/6-31G(d,p) level in the gas phase.

| Conformations | E+ZPE | Gibbs | Ratio |
| --- | --- | --- | --- |
| OME48 | -1343.872955 | -1343.483964 | 6.52% |
| BAL57 | -1343.87372 | -1344.644384 | 6.25% |
| BAL39 | -1343.873719 | -1344.644119 | 5.80% |
| BAL37 | -1343.87365 | -1344.645314 | 5.62% |
| BAL19 | -1343.873519 | -1344.643587 | 5.27% |
| BAL2 | -1343.873509 | -1344.645769 | 5.22% |
| BAL26 | -1343.873526 | -1344.643652 | 5.22% |
| BAL15 | -1343.873453 | -1344.643225 | 5.05% |
| BAL92 | -1343.873433 | -1344.647647 | 4.09% |
| OME44 | -1343.873032 | -1344.644978 | 2.94% |
| OME68 | -1343.872819 | -1344.643209 | 2.88% |
| OME12 | -1343.873082 | -1344.645186 | 2.68% |
| BAL100 | -1343.872975 | -1344.644319 | 2.62% |
| OME196 | -1343.872851 | -1344.645299 | 2.62% |
| OME137 | -1343.87306 | -1344.645438 | 2.59% |
| OME47 | -1343.872799 | -1344.643987 | 2.57% |
| BAL16 | -1343.872882 | -1344.6438 | 2.54% |
| BAL121 | -1343.872748 | -1344.641194 | 2.41% |
| OME143 | -1343.872878 | -1344.646528 | 2.31% |
| OME98 | -1343.872691 | -1344.644975 | 2.28% |
| OME69 | -1343.872685 | -1344.642247 | 2.21% |
| BAL68 | -1343.872672 | -1344.644206 | 2.17% |
| OME56 | -1343.872942 | -1344.648332 | 2.14% |
| BAL102 | -1343.872743 | -1344.644199 | 2.12% |
| BAL132 | -1343.869153 | -1344.641683 | 1.97% |
| BAL103 | -1343.872586 | -1344.64701 | 1.61% |
| BAL130 | -1343.872136 | -1344.64416 | 1.18% |
| BAL165 | -1343.872163 | -1344.643671 | 1.09% |
| BAL131 | -1343.871936 | -1344.643176 | 1.04% |
| BAL144 | -1343.871938 | -1344.644036 | 1.02% |

E+ZPE, G: total energy with zero point energy (ZPE) and Gibbs free energy in the gas phase at B3LYP/6-31G(d,p) level., %: Boltzmann distributions, using the relative Gibbs free energies as weighting factors.

**Table S4.** Optimized coordinate of (2*R*,3*R*)-**1** in the gas phase (Å) at B3LYP/6-31G(d,p) level.

| **OME48** | | | | **BAL57** | | | |
| --- | --- | --- | --- | --- | --- | --- | --- |
| C1 | -4.529 | -0.251 | -0.496 | C1 | -4.417 | -0.609 | -0.18 |
| C2 | -3.375 | 1.508 | 0.672 | C2 | -3.217 | 1.431 | 0.253 |
| C3 | 1.773 | -2.434 | -0.751 | C3 | 1.89 | -2.711 | -0.264 |
| C4 | -5.419 | 0.677 | -1.034 | C4 | -5.367 | 0.095 | -0.916 |
| C5 | -4.259 | 2.447 | 0.142 | C5 | -4.16 | 2.149 | -0.48 |
| C6 | 3.09 | -2.277 | -1.186 | C6 | 3.169 | -2.692 | -0.822 |
| C7 | 2.177 | -0.849 | 1.005 | C7 | 2.415 | -0.724 | 0.978 |
| C8 | -3.493 | 0.147 | 0.36 | C8 | -3.328 | 0.043 | 0.414 |
| C9 | 1.293 | -1.722 | 0.353 | C9 | 1.49 | -1.725 | 0.646 |
| C10 | 3.498 | -0.662 | 0.591 | C10 | 3.702 | -0.676 | 0.438 |
| C11 | -5.285 | 2.033 | -0.715 | C11 | -5.24 | 1.481 | -1.07 |
| C12 | 3.947 | -1.393 | -0.525 | C12 | 4.068 | -1.68 | -0.478 |
| C13 | 4.436 | 1.676 | 0.637 | C13 | 4.779 | 1.501 | -0.244 |
| C14 | 5.481 | 2.341 | 0.119 | C14 | 4.524 | 2.814 | -0.128 |
| C15 | -2.515 | -0.783 | 2.439 | C15 | -2.162 | -0.154 | 2.593 |
| C16 | -1.088 | -0.603 | -1.15 | C16 | -1.088 | -1.312 | -0.923 |
| C17 | 5.291 | 3.707 | -0.498 | C17 | 4.684 | 3.734 | -1.316 |
| C18 | 6.903 | 1.837 | 0.094 | C18 | 4.072 | 3.506 | 1.136 |
| C19 | -1.658 | -1.708 | -3.164 | C19 | -0.88 | -0.808 | -3.222 |
| C20 | 4.416 | 0.317 | 1.303 | C20 | 4.678 | 0.426 | 0.816 |
| C21 | -0.138 | -1.872 | 0.828 | C21 | 0.099 | -1.722 | 1.246 |
| C22 | -2.512 | -0.885 | 0.919 | C22 | -2.283 | -0.754 | 1.197 |
| C23 | -1.068 | -0.716 | 0.369 | C23 | -0.898 | -0.798 | 0.497 |
| O1 | -1.778 | -0.076 | 3.1 | O1 | -1.386 | 0.724 | 2.917 |
| O2 | -0.696 | 0.358 | -1.782 | O2 | -1.51 | -2.421 | -1.196 |
| O3 | -6.183 | 2.902 | -1.267 | O3 | -6.197 | 2.124 | -1.801 |
| O4 | 5.248 | -1.213 | -0.915 | O4 | 5.336 | -1.622 | -0.998 |
| O5 | -3.482 | -1.546 | 2.987 | O5 | -3.065 | -0.683 | 3.442 |
| O6 | -1.591 | -1.714 | -1.723 | O6 | -0.734 | -0.402 | -1.845 |
| H1 | -4.643 | -1.301 | -0.75 | H1 | -4.525 | -1.685 | -0.069 |
| H2 | -2.591 | 1.843 | 1.345 | H2 | -2.389 | 1.964 | 0.713 |
| H3 | 1.12 | -3.127 | -1.275 | H3 | 1.2 | -3.504 | -0.539 |
| H4 | -6.219 | 0.364 | -1.697 | H4 | -6.208 | -0.414 | -1.376 |
| H5 | -4.152 | 3.499 | 0.397 | H5 | -4.058 | 3.226 | -0.593 |
| H6 | 3.455 | -2.844 | -2.04 | H6 | 3.471 | -3.467 | -1.523 |
| H7 | 1.826 | -0.296 | 1.875 | H7 | 2.125 | 0.046 | 1.692 |
| H8 | 3.456 | 2.155 | 0.582 | H8 | 5.099 | 1.133 | -1.219 |
| H9 | 5.91 | 4.46 | 0.011 | H9 | 5.014 | 3.195 | -2.21 |
| H10 | 5.605 | 3.711 | -1.552 | H10 | 3.738 | 4.242 | -1.553 |
| H11 | 4.248 | 4.037 | -0.453 | H11 | 5.416 | 4.528 | -1.106 |
| H12 | 7.294 | 1.844 | -0.933 | H12 | 3.949 | 2.83 | 1.985 |
| H13 | 7.558 | 2.5 | 0.678 | H13 | 4.792 | 4.284 | 1.428 |
| H14 | 7.011 | 0.823 | 0.484 | H14 | 3.114 | 4.019 | 0.972 |
| H15 | -2.083 | -2.672 | -3.44 | H15 | -0.557 | 0.048 | -3.813 |
| H16 | -0.659 | -1.592 | -3.59 | H16 | -0.253 | -1.677 | -3.43 |
| H17 | -2.3 | -0.893 | -3.509 | H17 | -1.924 | -1.051 | -3.436 |
| H18 | 4.053 | 0.435 | 2.334 | H18 | 4.376 | 0.849 | 1.778 |
| H19 | 5.424 | -0.102 | 1.369 | H19 | 5.672 | -0.019 | 0.963 |
| H20 | -0.553 | -2.821 | 0.473 | H20 | 0.152 | -1.387 | 2.287 |
| H21 | -0.16 | -1.897 | 1.923 | H21 | -0.311 | -2.738 | 1.249 |
| H22 | -2.878 | -1.88 | 0.652 | H22 | -2.65 | -1.779 | 1.304 |
| H23 | -0.664 | 0.231 | 0.735 | H23 | -0.49 | 0.215 | 0.454 |
| H24 | -5.983 | 3.805 | -0.969 | H24 | -5.996 | 3.074 | -1.83 |
| H25 | 5.433 | -1.788 | -1.676 | H25 | 5.468 | -2.372 | -1.601 |
| H26 | -3.462 | -1.392 | 3.952 | H26 | -2.969 | -0.218 | 4.296 |
| **BAL39** | | | | **BAL37** | | | |
| C1 | 4.143 | -1.012 | -1.231 | C1 | 4.598 | 0.064 | 0.733 |
| C2 | 4.261 | 0.515 | 0.625 | C2 | 3.548 | -1.22 | -1.01 |
| C3 | -1.534 | 2.081 | 1.179 | C3 | -1.707 | 1.993 | 1.483 |
| C4 | 5.264 | -1.689 | -0.757 | C4 | 5.732 | 0.229 | -0.059 |
| C5 | 5.384 | -0.151 | 1.11 | C5 | 4.677 | -1.065 | -1.812 |
| C6 | -2.63 | 1.989 | 2.035 | C6 | -2.819 | 2.665 | 0.975 |
| C7 | -2.314 | 0.149 | -0.014 | C7 | -2.249 | 0.09 | 0.123 |
| C8 | 3.621 | 0.097 | -0.55 | C8 | 3.488 | -0.658 | 0.273 |
| C9 | -1.355 | 1.161 | 0.139 | C9 | -1.401 | 0.693 | 1.064 |
| C10 | -3.425 | 0.03 | 0.825 | C10 | -3.369 | 0.734 | -0.406 |
| C11 | 5.89 | -1.259 | 0.419 | C11 | 5.775 | -0.337 | -1.339 |
| C12 | -3.573 | 0.971 | 1.859 | C12 | -3.645 | 2.042 | 0.036 |
| C13 | -5.696 | -0.566 | -0.094 | C13 | -5.543 | -0.49 | -0.773 |
| C14 | -6.203 | -0.969 | -1.27 | C14 | -5.974 | -1.76 | -0.714 |
| C15 | 2.69 | 2.293 | -1.222 | C15 | 1.883 | -2.274 | 1.256 |
| C16 | 0.901 | -0.892 | 0.011 | C16 | 1.468 | 1.474 | 0.455 |
| C17 | -7.461 | -0.336 | -1.82 | C17 | -7.276 | -2.095 | -0.022 |
| C18 | -5.618 | -2.048 | -2.148 | C18 | -5.258 | -2.952 | -1.3 |
| C19 | 0.784 | -2.676 | 1.554 | C19 | 1.749 | 3.264 | -1.06 |
| C20 | -4.458 | -1.067 | 0.617 | C20 | -4.278 | 0.044 | -1.41 |
| C21 | -0.147 | 1.245 | -0.772 | C21 | -0.18 | -0.038 | 1.584 |
| C22 | 2.373 | 0.81 | -1.073 | C22 | 2.242 | -0.798 | 1.148 |
| C23 | 1.13 | 0.602 | -0.167 | C23 | 1.034 | 0.024 | 0.619 |
| O1 | 2.53 | 3.137 | -0.362 | O1 | 1.146 | -2.876 | 0.499 |
| O2 | 0.654 | -1.657 | -0.904 | O2 | 1.856 | 2.177 | 1.37 |
| O3 | 6.986 | -1.957 | 0.84 | O3 | 6.91 | -0.147 | -2.074 |
| O4 | -4.67 | 0.846 | 2.673 | O4 | -4.749 | 2.663 | -0.491 |
| O5 | 3.226 | 2.569 | -2.427 | O5 | 2.525 | -2.87 | 2.281 |
| O6 | 0.996 | -1.272 | 1.294 | O6 | 1.366 | 1.894 | -0.816 |
| H1 | 3.663 | -1.356 | -2.144 | H1 | 4.574 | 0.511 | 1.723 |
| H2 | 3.886 | 1.38 | 1.165 | H2 | 2.707 | -1.796 | -1.387 |
| H3 | -0.811 | 2.881 | 1.324 | H3 | -1.072 | 2.489 | 2.213 |
| H4 | 5.664 | -2.547 | -1.287 | H4 | 6.588 | 0.79 | 0.301 |
| H5 | 5.869 | 0.19 | 2.022 | H5 | 4.705 | -1.511 | -2.804 |
| H6 | -2.76 | 2.711 | 2.838 | H6 | -3.049 | 3.674 | 1.311 |
| H7 | -2.192 | -0.576 | -0.816 | H7 | -2.03 | -0.923 | -0.211 |
| H8 | -6.221 | 0.228 | 0.437 | H8 | -6.161 | 0.277 | -0.304 |
| H9 | -7.852 | 0.442 | -1.157 | H9 | -7.764 | -1.205 | 0.387 |
| H10 | -7.278 | 0.114 | -2.806 | H10 | -7.978 | -2.581 | -0.715 |
| H11 | -8.25 | -1.088 | -1.967 | H11 | -7.114 | -2.805 | 0.801 |
| H12 | -4.71 | -2.5 | -1.741 | H12 | -4.315 | -2.699 | -1.79 |
| H13 | -6.349 | -2.853 | -2.311 | H13 | -5.041 | -3.693 | -0.518 |
| H14 | -5.377 | -1.647 | -3.142 | H14 | -5.895 | -3.461 | -2.037 |
| H15 | 0.904 | -2.794 | 2.63 | H15 | 1.602 | 3.422 | -2.128 |
| H16 | -0.222 | -2.97 | 1.246 | H16 | 1.118 | 3.943 | -0.481 |
| H17 | 1.522 | -3.276 | 1.017 | H17 | 2.795 | 3.419 | -0.788 |
| H18 | -3.992 | -1.889 | 0.067 | H18 | -4.554 | 0.767 | -2.19 |
| H19 | -4.75 | -1.466 | 1.597 | H19 | -3.717 | -0.755 | -1.903 |
| H20 | 0.074 | 2.296 | -0.987 | H20 | -0.426 | -1.092 | 1.749 |
| H21 | -0.36 | 0.754 | -1.728 | H21 | 0.126 | 0.382 | 2.549 |
| H22 | 2.147 | 0.414 | -2.068 | H22 | 2.49 | -0.439 | 2.151 |
| H23 | 1.328 | 1.046 | 0.812 | H23 | 0.74 | -0.369 | -0.357 |
| H24 | 7.325 | -1.562 | 1.66 | H24 | 6.813 | -0.585 | -2.936 |
| H25 | -4.652 | 1.549 | 3.343 | H25 | -4.818 | 3.557 | -0.12 |
| H26 | 3.454 | 3.52 | -2.433 | H26 | 2.292 | -3.819 | 2.258 |
| **BAL19** | | | | **BAL2** | | | |
| C1 | -4.763 | 0.121 | -0.685 | C1 | 4.34 | 0.228 | 0.907 |
| C2 | -3.758 | -0.904 | 1.246 | C2 | 4.375 | -0.597 | -1.353 |
| C3 | 1.683 | 1.353 | -1.799 | C3 | -1.526 | 1.587 | 1.459 |
| C4 | -5.863 | 0.503 | 0.08 | C4 | 5.419 | -0.589 | 1.23 |
| C5 | -4.853 | -0.529 | 2.023 | C5 | 5.455 | -1.422 | -1.043 |
| C6 | 2.865 | 1.992 | -1.423 | C6 | -2.694 | 1.209 | 2.121 |
| C7 | 2.094 | -0.347 | -0.156 | C7 | -2.15 | 0.212 | -0.406 |
| C8 | -3.693 | -0.585 | -0.117 | C8 | 3.798 | 0.237 | -0.388 |
| C9 | 1.276 | 0.171 | -1.171 | C9 | -1.231 | 1.092 | 0.184 |
| C10 | 3.284 | 0.265 | 0.243 | C10 | -3.333 | -0.182 | 0.227 |
| C11 | -5.912 | 0.178 | 1.44 | C11 | 5.981 | -1.421 | 0.254 |
| C12 | 3.664 | 1.454 | -0.411 | C12 | -3.595 | 0.331 | 1.51 |
| C13 | 5.269 | -1.203 | 0.753 | C13 | -5.451 | -0.358 | -1.128 |
| C14 | 6.597 | -1.066 | 0.9 | C14 | -6.772 | -0.456 | -0.906 |
| C15 | -2.26 | -2.475 | -0.837 | C15 | 2.913 | 2.553 | -0.363 |
| C16 | -1.498 | 1.3 | -0.668 | C16 | 1.049 | -0.823 | -0.409 |
| C17 | 7.546 | -2.048 | 0.253 | C17 | -7.749 | 0.38 | -1.701 |
| C18 | 7.273 | 0.026 | 1.693 | C18 | -7.415 | -1.372 | 0.106 |
| C19 | -1.575 | 3.332 | 0.535 | C19 | 0.767 | -3.007 | 0.444 |
| C20 | 4.162 | -0.343 | 1.324 | C20 | -4.322 | -1.11 | -0.457 |
| C21 | -0.018 | -0.52 | -1.549 | C21 | 0.055 | 1.473 | -0.52 |
| C22 | -2.485 | -0.975 | -0.97 | C22 | 2.595 | 1.113 | -0.742 |
| C23 | -1.198 | -0.19 | -0.595 | C23 | 1.282 | 0.641 | -0.06 |
| O1 | -1.555 | -3.01 | -0.002 | O1 | 2.681 | 3.065 | 0.716 |
| O2 | -1.847 | 1.877 | -1.681 | O2 | 0.905 | -1.239 | -1.544 |
| O3 | -7.014 | 0.573 | 2.144 | O3 | 7.037 | -2.204 | 0.625 |
| O4 | 4.83 | 2.05 | -0.009 | O4 | -4.752 | -0.067 | 2.128 |
| O5 | -2.983 | -3.17 | -1.737 | O5 | 3.538 | 3.206 | -1.362 |
| O6 | -1.322 | 1.911 | 0.515 | O6 | 1.007 | -1.604 | 0.681 |
| H1 | -4.735 | 0.381 | -1.739 | H1 | 3.92 | 0.875 | 1.672 |
| H2 | -2.949 | -1.462 | 1.71 | H2 | 3.972 | -0.61 | -2.363 |
| H3 | 1.073 | 1.783 | -2.589 | H3 | -0.838 | 2.275 | 1.946 |
| H4 | -6.689 | 1.05 | -0.364 | H4 | 5.836 | -0.592 | 2.233 |
| H5 | -4.886 | -0.787 | 3.079 | H5 | 5.889 | -2.064 | -1.806 |
| H6 | 3.172 | 2.91 | -1.921 | H6 | -2.912 | 1.6 | 3.113 |
| H7 | 1.794 | -1.266 | 0.346 | H7 | -1.938 | -0.187 | -1.396 |
| H8 | 4.913 | -2.037 | 0.146 | H8 | -5.119 | 0.35 | -1.889 |
| H9 | 7.014 | -2.827 | -0.302 | H9 | -7.242 | 1.027 | -2.424 |
| H10 | 8.229 | -1.538 | -0.441 | H10 | -8.458 | -0.256 | -2.249 |
| H11 | 8.18 | -2.538 | 1.006 | H11 | -8.353 | 1.016 | -1.038 |
| H12 | 6.577 | 0.768 | 2.088 | H12 | -6.693 | -1.92 | 0.715 |
| H13 | 7.836 | -0.399 | 2.536 | H13 | -8.059 | -0.798 | 0.786 |
| H14 | 8.006 | 0.554 | 1.067 | H14 | -8.067 | -2.103 | -0.392 |
| H15 | -1.389 | 3.646 | 1.562 | H15 | 0.768 | -3.473 | 1.429 |
| H16 | -0.898 | 3.847 | -0.152 | H16 | -0.199 | -3.148 | -0.046 |
| H17 | -2.609 | 3.537 | 0.251 | H17 | 1.559 | -3.427 | -0.18 |
| H18 | 4.567 | 0.449 | 1.958 | H18 | -3.778 | -1.684 | -1.221 |
| H19 | 3.526 | -0.969 | 1.965 | H19 | -4.71 | -1.834 | 0.265 |
| H20 | 0.129 | -1.605 | -1.538 | H20 | 0.275 | 2.529 | -0.333 |
| H21 | -0.31 | -0.239 | -2.567 | H21 | -0.058 | 1.349 | -1.603 |
| H22 | -2.726 | -0.764 | -2.016 | H22 | 2.455 | 1.075 | -1.826 |
| H23 | -0.915 | -0.444 | 0.43 | H23 | 1.392 | 0.731 | 1.023 |
| H24 | -6.928 | 0.282 | 3.067 | H24 | 7.33 | -2.732 | -0.135 |
| H25 | 4.959 | 2.868 | -0.517 | H25 | -4.798 | 0.341 | 3.007 |
| H26 | -2.835 | -4.119 | -1.56 | H26 | 3.759 | 4.099 | -1.033 |
| **BAL26** | | | | **BAL15** | | | |
| C1 | -4.579 | -0.217 | -0.603 | C1 | -4.165 | 0.929 | -1.159 |
| C2 | -3.464 | 1.127 | 1.053 | C2 | -4.001 | 0.132 | 1.105 |
| C3 | 1.72 | -2.081 | -1.501 | C3 | 1.507 | -2.362 | 1.036 |
| C4 | -5.483 | 0.815 | -0.844 | C4 | -5.037 | 1.936 | -0.751 |
| C5 | -4.363 | 2.167 | 0.824 | C5 | -4.873 | 1.134 | 1.527 |
| C6 | 3.03 | -1.779 | -1.875 | C6 | 2.794 | -2.246 | 1.559 |
| C7 | 2.15 | -1.184 | 0.683 | C7 | 2.186 | -1.102 | -0.89 |
| C8 | -3.556 | -0.08 | 0.345 | C8 | -3.631 | 0.014 | -0.242 |
| C9 | 1.256 | -1.788 | -0.214 | C9 | 1.179 | -1.791 | -0.199 |
| C10 | 3.466 | -0.864 | 0.34 | C10 | 3.485 | -0.964 | -0.393 |
| C11 | -5.377 | 2.014 | -0.13 | C11 | -5.394 | 2.042 | 0.598 |
| C12 | 3.898 | -1.17 | -0.964 | C12 | 3.779 | -1.548 | 0.853 |
| C13 | 4.457 | 1.31 | 1.144 | C13 | 4.666 | 1.244 | -0.697 |
| C14 | 5.527 | 2.084 | 0.902 | C14 | 5.74 | 1.877 | -0.199 |
| C15 | -2.54 | -1.554 | 2.059 | C15 | -3.202 | -2.426 | -0.272 |
| C16 | -1.212 | -0.47 | -1.394 | C16 | -0.753 | 0.538 | -0.554 |
| C17 | 5.376 | 3.581 | 0.758 | C17 | 5.658 | 3.334 | 0.193 |
| C18 | 6.944 | 1.585 | 0.757 | C18 | 7.095 | 1.248 | 0.015 |
| C19 | -0.802 | 1.25 | -2.96 | C19 | -0.074 | 2.651 | 0.251 |
| C20 | 4.399 | -0.19 | 1.332 | C20 | 4.545 | -0.192 | -1.161 |
| C21 | -0.168 | -2.095 | 0.203 | C21 | -0.224 | -1.905 | -0.76 |
| C22 | -2.558 | -1.216 | 0.574 | C22 | -2.658 | -1.071 | -0.706 |
| C23 | -1.128 | -0.882 | 0.069 | C23 | -1.213 | -0.863 | -0.174 |
| O1 | -1.802 | -1.055 | 2.887 | O1 | -2.95 | -2.983 | 0.779 |
| O2 | -1.62 | -1.194 | -2.284 | O2 | -0.614 | 0.921 | -1.701 |
| O3 | -6.289 | 2.993 | -0.405 | O3 | -6.253 | 3.045 | 0.945 |
| O4 | 5.194 | -0.865 | -1.288 | O4 | 5.058 | -1.418 | 1.328 |
| O5 | -3.489 | -2.457 | 2.376 | O5 | -4.056 | -2.934 | -1.183 |
| O6 | -0.774 | 0.783 | -1.595 | O6 | -0.515 | 1.305 | 0.521 |
| H1 | -4.669 | -1.142 | -1.166 | H1 | -3.893 | 0.858 | -2.209 |
| H2 | -2.689 | 1.256 | 1.802 | H2 | -3.614 | -0.572 | 1.837 |
| H3 | 1.056 | -2.554 | -2.22 | H3 | 0.753 | -2.913 | 1.595 |
| H4 | -6.274 | 0.704 | -1.58 | H4 | -5.447 | 2.643 | -1.466 |
| H5 | -4.279 | 3.094 | 1.385 | H5 | -5.15 | 1.209 | 2.577 |
| H6 | 3.382 | -2.02 | -2.876 | H6 | 3.039 | -2.703 | 2.516 |
| H7 | 1.813 | -0.96 | 1.694 | H7 | 1.953 | -0.659 | -1.856 |
| H8 | 3.487 | 1.803 | 1.222 | H8 | 3.742 | 1.818 | -0.794 |
| H9 | 5.732 | 3.921 | -0.224 | H9 | 4.662 | 3.751 | 0.014 |
| H10 | 4.335 | 3.901 | 0.873 | H10 | 5.901 | 3.472 | 1.256 |
| H11 | 5.981 | 4.113 | 1.506 | H11 | 6.386 | 3.936 | -0.37 |
| H12 | 7.03 | 0.498 | 0.815 | H12 | 7.116 | 0.18 | -0.208 |
| H13 | 7.366 | 1.904 | -0.206 | H13 | 7.854 | 1.743 | -0.607 |
| H14 | 7.588 | 2.019 | 1.534 | H14 | 7.416 | 1.379 | 1.057 |
| H15 | -0.419 | 2.27 | -2.926 | H15 | 0.051 | 3.118 | 1.228 |
| H16 | -0.166 | 0.622 | -3.588 | H16 | 0.874 | 2.637 | -0.292 |
| H17 | -1.824 | 1.236 | -3.345 | H17 | -0.823 | 3.186 | -0.337 |
| H18 | 4.028 | -0.404 | 2.344 | H18 | 5.505 | -0.709 | -1.088 |
| H19 | 5.396 | -0.632 | 1.26 | H19 | 4.263 | -0.195 | -2.223 |
| H20 | -0.181 | -2.42 | 1.248 | H20 | -0.622 | -2.903 | -0.548 |
| H21 | -0.567 | -2.917 | -0.401 | H21 | -0.204 | -1.784 | -1.849 |
| H22 | -2.918 | -2.096 | 0.034 | H22 | -2.629 | -1.055 | -1.799 |
| H23 | -0.733 | -0.044 | 0.648 | H23 | -1.222 | -0.947 | 0.916 |
| H24 | -6.106 | 3.769 | 0.15 | H24 | -6.423 | 3.008 | 1.901 |
| H25 | 5.369 | -1.15 | -2.2 | H25 | 5.129 | -1.884 | 2.177 |
| H26 | -3.458 | -2.585 | 3.345 | H26 | -4.407 | -3.77 | -0.818 |
| **BAL92** | | | | | | | |
| C1 | -3.828 | 1.113 | -1.064 | O6 | -0.162 | 0.971 | 0.89 |
| C2 | -3.869 | -0.072 | 1.03 | H1 | -3.476 | 1.212 | -2.087 |
| C3 | 1.62 | -2.713 | 0.907 | H2 | -3.565 | -0.909 | 1.651 |
| C4 | -4.695 | 2.073 | -0.548 | H3 | 0.851 | -3.36 | 1.322 |
| C5 | -4.738 | 0.881 | 1.559 | H4 | -5.024 | 2.913 | -1.153 |
| C6 | 2.875 | -2.67 | 1.512 | H5 | -5.095 | 0.785 | 2.582 |
| C7 | 2.367 | -1.127 | -0.733 | H6 | 3.08 | -3.28 | 2.39 |
| C8 | -3.399 | 0.029 | -0.286 | H7 | 2.174 | -0.521 | -1.616 |
| C9 | 1.342 | -1.938 | -0.226 | H8 | 4.986 | 1.061 | 1.108 |
| C10 | 3.634 | -1.057 | -0.148 | H9 | 4.97 | 3.314 | 1.513 |
| C11 | -5.154 | 1.96 | 0.77 | H10 | 5.591 | 4.276 | 0.155 |
| C12 | 3.876 | -1.842 | 0.994 | H11 | 3.86 | 4.218 | 0.462 |
| C13 | 4.791 | 1.171 | 0.041 | H12 | 4.28 | 1.896 | -2.534 |
| C14 | 4.654 | 2.417 | -0.441 | H13 | 3.478 | 3.373 | -1.975 |
| C15 | -3.055 | -2.385 | -0.724 | H14 | 5.208 | 3.384 | -2.288 |
| C16 | -0.471 | 0.452 | -0.308 | H15 | 0.542 | 2.567 | 1.922 |
| C17 | 4.777 | 3.612 | 0.477 | H16 | 1.425 | 2.235 | 0.399 |
| C18 | 4.391 | 2.769 | -1.886 | H17 | -0.194 | 2.991 | 0.342 |
| C19 | 0.442 | 2.282 | 0.876 | H18 | 5.681 | -0.651 | -0.637 |
| C20 | 4.711 | -0.142 | -0.708 | H19 | 4.517 | 0.023 | -1.772 |
| C21 | -0.032 | -1.948 | -0.863 | H20 | -0.462 | -2.953 | -0.797 |
| C22 | -2.429 | -1.003 | -0.864 | H21 | 0.044 | -1.692 | -1.926 |
| C23 | -1.03 | -0.96 | -0.195 | H22 | -2.308 | -0.796 | -1.931 |
| O1 | -2.921 | -3.126 | 0.231 | H23 | -1.131 | -1.214 | 0.863 |
| O2 | -0.301 | 1.035 | -1.364 | H24 | -6.244 | 2.736 | 2.148 |
| O3 | -6.003 | 2.927 | 1.227 | H25 | 5.164 | -2.357 | 2.326 |
| O4 | 5.123 | -1.761 | 1.56 | H26 | -4.245 | -3.556 | -1.601 |
| O5 | -3.834 | -2.688 | -1.781 |  |  |  |  |

**
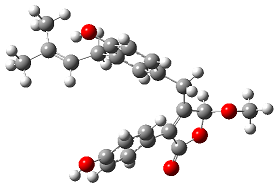

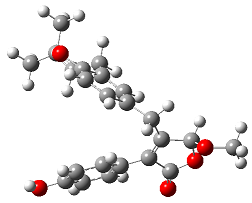

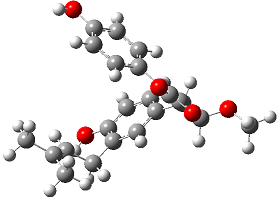
**

**a b c**

**
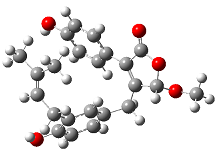

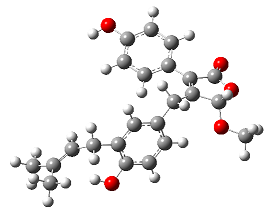

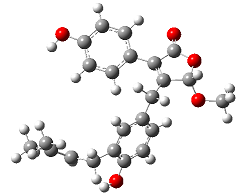
**

**d e f**

**
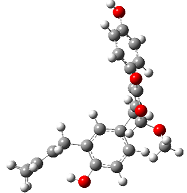

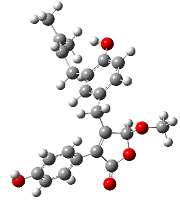

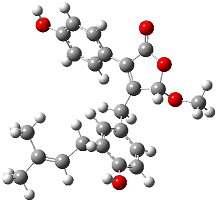
**

**g h i**

**
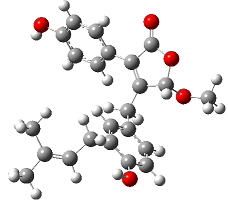

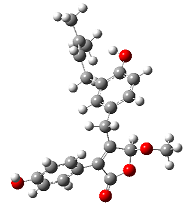

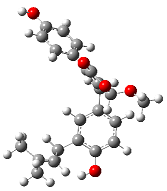
**

**j k l**

**
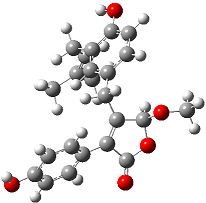

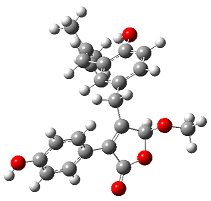

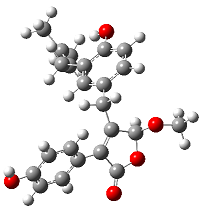
**

**m n o**

**
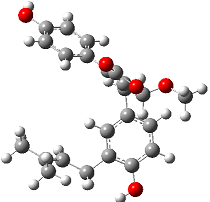
**

**p**

**Figure S53**. Optimized geometries of predominant conformers for compound (4*S*)-**2** at the B3LYP/6-31G(d,p) level in the gas phase.

**Table S5**. Important thermodynamic parameters (a.u.) and Boltzmann distributions of the optimized compound (4*S*)-**2** at B3LYP/6-31G(d,p) level in the gas phase.

| Conformations | E+ZPE | Gibbs | % |
| --- | --- | --- | --- |
| (4*S*)-**2**-a | -1266.614922 | -1266.675344 | 0.1 |
| (4*S*)-**2**-b | -1266.614933 | -1266.675184 | 0.1 |
| (4*S*)-**2**-c | -1266.610491 | -1266.672192 | 0 |
| (4*S*)-**2**-d | -1266.610410 | -1266.671367 | 0 |
| (4*S*)-**2**-e | -1266.616780 | -1266.676349 | 0.3 |
| (4*S*)-**2**-f | -1266.612791 | -1266.673668 | 0 |
| (4*S*)-**2**-g | -1266.619600 | -1266.680117 | 14.9 |
| (4*S*)-**2**-h | -1266.619291 | -1266.679808 | 10.8 |
| (4*S*)-**2**-i | -1266.615336 | -1266.676908 | 0.5 |
| (4*S*)-**2**-j | -1266.615054 | -1266.676657 | 0.4 |
| (4*S*)-**2**-k | -1266.619291 | -1266.679814 | 10.8 |
| (4*S*)-**2**-l | -1266.615363 | -1266.676783 | 0.4 |
| (4*S*)-**2**-m | -1266.615086 | -1266.676506 | 0.3 |
| (4*S*)-**2**-n | -1266.619648 | -1266.680684 | 27.2 |
| (4*S*)-**2**-o | -1266.619396 | -1266.680730 | 28.6 |
| (4*S*)-**2**-p | -1266.615387 | -1266.679197 | 5.6 |

E+ZPE, G: total energy with zero point energy (ZPE) and Gibbs free energy in the gas phase at B3LYP/6-31G(d,p) level., %: Boltzmann distributions, using the relative Gibbs free energies as weighting factors.

**Table S6.** Optimized Z-matrixes of compound (4*S*)-**2** in the gas phase (Å) at B3LYP/6-31G(d,p) level.

| (4*S*)-**2**-a | | | | (4*S*)-**2**-b | | | |
| --- | --- | --- | --- | --- | --- | --- | --- |
| O | 0.655799 | 4.94178 | 1.078525 | O | 0.965432 | 4.800929 | 1.018277 |
| C | -1.18149 | 2.524204 | -1.0066 | C | -1.15624 | 1.809439 | 1.328486 |
| C | -0.42888 | 3.641049 | -0.65843 | C | -0.337 | 2.870924 | 1.69717 |
| C | -0.07564 | 3.86241 | 0.676596 | C | 0.151337 | 3.746787 | 0.721707 |
| C | -0.48385 | 2.955221 | 1.66076 | C | -0.20104 | 3.557352 | -0.61907 |
| C | -1.22834 | 1.839874 | 1.303164 | C | -1.02657 | 2.49969 | -0.97659 |
| C | -1.58972 | 1.592525 | -0.03506 | C | -1.5122 | 1.593343 | -0.01287 |
| C | 5.869584 | 0.678 | -1.72184 | C | 5.827852 | 0.653858 | -1.70611 |
| C | 5.788521 | -1.86036 | -1.72336 | C | 5.738764 | -1.88402 | -1.74836 |
| C | 5.039894 | -0.5665 | -1.51245 | C | 4.993461 | -0.59116 | -1.51979 |
| C | 3.735219 | -0.47222 | -1.19274 | C | 3.687584 | -0.49758 | -1.20464 |
| C | 2.738774 | -1.58971 | -0.97125 | C | 2.686481 | -1.61543 | -1.00698 |
| O | 3.88739 | -1.25421 | 1.701156 | O | 3.82845 | -1.34126 | 1.674319 |
| C | -5.52174 | -2.82276 | -0.35894 | C | -5.68005 | -2.59398 | -0.37551 |
| O | -4.5468 | -1.97338 | 0.242728 | O | -4.64939 | -1.8159 | 0.229681 |
| O | -3.52742 | 1.322378 | -2.38178 | O | -3.49744 | 1.489722 | -2.32834 |
| C | 0.51383 | -1.45578 | 0.244337 | C | 0.458584 | -1.48614 | 0.203457 |
| C | 1.910283 | -1.43022 | 0.293641 | C | 1.854649 | -1.47505 | 0.257821 |
| C | 2.529943 | -1.28859 | 1.551508 | C | 2.471208 | -1.36373 | 1.520479 |
| C | 1.75358 | -1.19879 | 2.709564 | C | 1.691735 | -1.29284 | 2.677636 |
| C | 0.365265 | -1.23754 | 2.626513 | C | 0.303082 | -1.316 | 2.588981 |
| C | -0.28058 | -1.35866 | 1.391065 | C | -0.33928 | -1.40298 | 1.349115 |
| C | -1.79791 | -1.46078 | 1.327848 | C | -1.85694 | -1.48012 | 1.27471 |
| C | -3.31745 | 0.441728 | -1.58516 | C | -3.32368 | 0.577685 | -1.55955 |
| O | -3.95461 | -0.77103 | -1.67288 | O | -4.02626 | -0.59768 | -1.66653 |
| C | -3.50565 | -1.61795 | -0.61057 | C | -3.6056 | -1.4943 | -0.63406 |
| C | -2.47875 | -0.81393 | 0.150925 | C | -2.52395 | -0.76646 | 0.12865 |
| C | -2.38216 | 0.412618 | -0.41055 | C | -2.37307 | 0.467461 | -0.40283 |
| H | 0.850944 | 5.494689 | 0.310292 | H | 1.12626 | 4.818399 | 1.971043 |
| H | -1.47048 | 2.377026 | -2.03928 | H | -1.53512 | 1.15084 | 2.100901 |
| H | -0.12096 | 4.347222 | -1.42686 | H | -0.07763 | 3.019594 | 2.743108 |
| H | -0.21347 | 3.142817 | 2.694201 | H | 0.175598 | 4.250486 | -1.36371 |
| H | -1.5489 | 1.158679 | 2.082023 | H | -1.31287 | 2.374474 | -2.01321 |
| H | 6.272314 | 0.709571 | -2.74254 | H | 6.235699 | 0.699273 | -2.72422 |
| H | 5.289358 | 1.589368 | -1.55685 | H | 5.249595 | 1.56451 | -1.53091 |
| H | 6.734625 | 0.694822 | -1.04669 | H | 6.689708 | 0.65767 | -1.02666 |
| H | 6.689152 | -1.89386 | -1.09789 | H | 6.637638 | -1.92972 | -1.12113 |
| H | 5.19363 | -2.74937 | -1.50795 | H | 5.140782 | -2.77457 | -1.5482 |
| H | 6.131492 | -1.93272 | -2.76343 | H | 6.084282 | -1.94137 | -2.78851 |
| H | 3.313944 | 0.528049 | -1.08965 | H | 3.268613 | 0.502147 | -1.08824 |
| H | 2.050423 | -1.62434 | -1.82573 | H | 2.000817 | -1.63193 | -1.8641 |
| H | 3.2482 | -2.5606 | -0.95408 | H | 3.191991 | -2.58849 | -1.00531 |
| H | 4.290251 | -1.01814 | 0.848461 | H | 4.234655 | -1.09023 | 0.827303 |
| H | -6.22741 | -3.09261 | 0.428329 | H | -6.38491 | -2.84768 | 0.417874 |
| H | -6.05242 | -2.30746 | -1.16642 | H | -6.19648 | -2.02798 | -1.15788 |
| H | -5.05874 | -3.73644 | -0.75876 | H | -5.2749 | -3.51954 | -0.80931 |
| H | 0.034745 | -1.55208 | -0.72719 | H | -0.01806 | -1.55772 | -0.77134 |
| H | 2.258717 | -1.08813 | 3.663442 | H | 2.194602 | -1.20938 | 3.635563 |
| H | -0.22365 | -1.16404 | 3.537738 | H | -0.2886 | -1.2571 | 3.499549 |
| H | -2.09199 | -2.51879 | 1.334353 | H | -2.16625 | -2.53297 | 1.228074 |
| H | -2.22479 | -1.05422 | 2.254075 | H | -2.28354 | -1.11362 | 2.21783 |
| H | -3.0763 | -2.5168 | -1.08544 | H | -3.23294 | -2.40203 | -1.13891 |
| (4*S*)-**2**-c | | | | (4*S*)-**2**-d | | | |
| O | -1.44424 | -4.67704 | 1.734945 | O | -2.46246 | -3.44303 | 2.500508 |
| C | 0.544677 | -2.64981 | -0.61054 | C | 0.295213 | -1.06589 | 1.963259 |
| C | -0.3065 | -3.64538 | -0.14286 | C | -0.77548 | -1.70263 | 2.58078 |
| C | -0.61987 | -3.72114 | 1.217984 | C | -1.40476 | -2.78096 | 1.949799 |
| C | -0.0703 | -2.79132 | 2.10762 | C | -0.94112 | -3.22134 | 0.70522 |
| C | 0.772349 | -1.79716 | 1.631101 | C | 0.132727 | -2.58322 | 0.098634 |
| C | 1.09687 | -1.69638 | 0.264427 | C | 0.769467 | -1.48251 | 0.707904 |
| C | -4.42429 | -0.85776 | -2.63264 | C | -4.25625 | -1.66661 | -1.74788 |
| C | -2.8308 | 0.829911 | -3.6559 | C | -2.28399 | -0.94856 | -3.17092 |
| C | -3.58232 | 0.37905 | -2.4282 | C | -3.30018 | -0.56107 | -2.12605 |
| C | -3.54375 | 0.985463 | -1.23253 | C | -3.37845 | 0.639051 | -1.53266 |
| C | -2.75511 | 2.214197 | -0.83242 | C | -2.48189 | 1.841321 | -1.73565 |
| O | -3.88621 | 2.191964 | 1.876708 | O | -3.60322 | 3.237805 | 0.597516 |
| C | 5.465239 | 2.214182 | -0.59373 | C | 5.871433 | 1.213096 | -0.62464 |
| O | 4.44917 | 1.514966 | 0.122442 | O | 4.66732 | 0.964383 | 0.098385 |
| O | 2.898351 | -1.79375 | -2.20748 | O | 2.771161 | -2.68244 | -1.26629 |
| C | -0.55627 | 1.773605 | 0.358967 | C | -0.34837 | 1.804542 | -0.36304 |
| C | -1.93402 | 1.998642 | 0.429134 | C | -1.68925 | 2.182105 | -0.48202 |
| C | -2.54243 | 1.991438 | 1.697346 | C | -2.29387 | 2.83294 | 0.607762 |
| C | -1.7807 | 1.767382 | 2.845926 | C | -1.5656 | 3.085516 | 1.772637 |
| C | -0.41167 | 1.543958 | 2.740613 | C | -0.23685 | 2.684357 | 1.86361 |
| C | 0.226162 | 1.544529 | 1.494808 | C | 0.397086 | 2.037841 | 0.79548 |
| C | 1.737645 | 1.382598 | 1.40631 | C | 1.873731 | 1.680854 | 0.894575 |
| C | 2.83856 | -0.84932 | -1.46001 | C | 2.813301 | -1.53949 | -0.88483 |
| O | 3.597262 | 0.276101 | -1.66754 | O | 3.789248 | -0.6761 | -1.31654 |
| C | 3.318581 | 1.229827 | -0.63838 | C | 3.591021 | 0.603024 | -0.70779 |
| C | 2.265165 | 0.594272 | 0.236528 | C | 2.347526 | 0.46817 | 0.138589 |
| C | 1.992564 | -0.64346 | -0.23582 | C | 1.897051 | -0.80451 | 0.05203 |
| H | -1.74345 | -5.25591 | 1.021231 | H | -2.67826 | -3.04234 | 3.35304 |
| H | 0.798219 | -2.61637 | -1.66218 | H | 0.774402 | -0.24168 | 2.47722 |
| H | -0.72493 | -4.37025 | -0.83817 | H | -1.12198 | -1.36238 | 3.554144 |
| H | -0.31143 | -2.86611 | 3.162406 | H | -1.4276 | -4.06899 | 0.234199 |
| H | 1.1996 | -1.09853 | 2.339729 | H | 0.499976 | -2.94757 | -0.8523 |
| H | -3.80356 | -1.70614 | -2.94932 | H | -3.71113 | -2.5247 | -1.33372 |
| H | -4.95464 | -1.14711 | -1.72155 | H | -4.98618 | -1.3401 | -1.00238 |
| H | -5.16726 | -0.70358 | -3.42623 | H | -4.80341 | -2.03564 | -2.62512 |
| H | -3.52935 | 1.069553 | -4.4681 | H | -2.78191 | -1.32224 | -4.07459 |
| H | -2.20441 | 1.707732 | -3.48833 | H | -1.62569 | -0.13006 | -3.46726 |
| H | -2.1868 | 0.023711 | -4.02954 | H | -1.65291 | -1.76674 | -2.80112 |
| H | -4.13228 | 0.541007 | -0.42938 | H | -4.14994 | 0.768524 | -0.77282 |
| H | -2.08859 | 2.53059 | -1.63785 | H | -1.7839 | 1.675821 | -2.55917 |
| H | -3.44344 | 3.064905 | -0.68451 | H | -3.08911 | 2.708563 | -2.04616 |
| H | -4.29997 | 2.391004 | 1.027665 | H | -3.982 | 3.079274 | -0.27615 |
| H | 6.253924 | 2.439376 | 0.125861 | H | 6.60537 | 1.560963 | 0.103985 |
| H | 5.87232 | 1.60075 | -1.40428 | H | 6.238108 | 0.301905 | -1.10865 |
| H | 5.0788 | 3.154313 | -1.0131 | H | 5.722218 | 1.990624 | -1.38768 |
| H | -0.08697 | 1.768366 | -0.62189 | H | 0.118014 | 1.294552 | -1.20187 |
| H | -2.28006 | 1.766607 | 3.809126 | H | -2.0606 | 3.589204 | 2.596419 |
| H | 0.168664 | 1.370955 | 3.643627 | H | 0.316271 | 2.883353 | 2.778535 |
| H | 2.20786 | 2.373909 | 1.371455 | H | 2.474969 | 2.533568 | 0.552046 |
| H | 2.106516 | 0.937274 | 2.338806 | H | 2.144917 | 1.567154 | 1.952667 |
| H | 2.957923 | 2.141959 | -1.1444 | H | 3.468961 | 1.327891 | -1.53075 |
| (4*S*)-**2**-e | | | | (4*S*)-**2**-f | | | |
| O | 1.619402 | 5.526832 | -0.11531 | O | -0.09242 | 5.404632 | -0.48731 |
| C | 1.248092 | 1.863888 | -0.0233 | C | 0.560197 | 1.790782 | -0.21602 |
| C | 0.900797 | 3.210575 | -0.07674 | C | -0.14031 | 2.982026 | -0.3799 |
| C | 1.897111 | 4.191941 | -0.06785 | C | 0.537982 | 4.204233 | -0.33612 |
| C | 3.243114 | 3.811901 | -0.01669 | C | 1.923479 | 4.22113 | -0.13766 |
| C | 3.583136 | 2.467303 | 0.030579 | C | 2.617021 | 3.029322 | 0.018824 |
| C | 2.593649 | 1.461829 | 0.04086 | C | 1.949988 | 1.786581 | -0.00465 |
| C | -6.68562 | 1.664428 | -0.91018 | C | -5.93517 | 1.574258 | -0.62902 |
| C | -7.05711 | -0.36455 | 0.571054 | C | -5.57682 | 0.877316 | 1.784435 |
| C | -6.07238 | 0.626429 | -0.0005 | C | -5.32538 | 0.577128 | 0.327551 |
| C | -4.75241 | 0.644419 | 0.265287 | C | -4.63365 | -0.47499 | -0.13488 |
| C | -3.95902 | -0.28393 | 1.159566 | C | -3.95211 | -1.56855 | 0.66057 |
| O | -3.86767 | -1.66744 | -1.42548 | O | -3.25675 | -3.03516 | -1.78772 |
| C | 3.25775 | -4.47188 | -0.21096 | C | 4.232296 | -3.72976 | 0.100054 |
| O | 2.532 | -3.24508 | -0.14219 | O | 3.195354 | -2.75018 | 0.05502 |
| O | 5.160274 | 0.131362 | -0.96964 | O | 4.86844 | 1.197373 | -0.72124 |
| C | -1.45068 | -0.68271 | 1.167799 | C | -1.43583 | -1.34828 | 0.931568 |
| C | -2.68417 | -0.81366 | 0.522281 | C | -2.52675 | -1.82943 | 0.201242 |
| C | -2.71441 | -1.48597 | -0.71563 | C | -2.26327 | -2.53333 | -0.98714 |
| C | -1.53833 | -2.01427 | -1.25436 | C | -0.94828 | -2.74075 | -1.40868 |
| C | -0.32741 | -1.88496 | -0.58017 | C | 0.118342 | -2.25361 | -0.65902 |
| C | -0.26352 | -1.20963 | 0.644944 | C | -0.11027 | -1.54764 | 0.528944 |
| C | 1.028088 | -1.06745 | 1.450387 | C | 1.018669 | -1.02912 | 1.419342 |
| C | 4.275549 | -0.46256 | -0.40603 | C | 4.131509 | 0.396091 | -0.20389 |
| O | 4.367932 | -1.80598 | -0.13883 | O | 4.562575 | -0.86164 | 0.139275 |
| C | 3.190426 | -2.23596 | 0.553244 | C | 3.488438 | -1.58244 | 0.752866 |
| C | 2.321067 | -1.00649 | 0.68399 | C | 2.308706 | -0.63768 | 0.751628 |
| C | 2.969592 | 0.04398 | 0.132087 | C | 2.692285 | 0.534526 | 0.197394 |
| H | 0.662131 | 5.649584 | -0.16442 | H | -1.03487 | 5.250732 | -0.63558 |
| H | 0.461733 | 1.119264 | -0.06315 | H | 0.020093 | 0.854018 | -0.28585 |
| H | -0.14698 | 3.498021 | -0.1365 | H | -1.21417 | 2.958384 | -0.55327 |
| H | 4.006455 | 4.582603 | -0.01974 | H | 2.438471 | 5.175578 | -0.11476 |
| H | 4.627617 | 2.183276 | 0.049853 | H | 3.691314 | 3.05244 | 0.151079 |
| H | -7.44948 | 2.246001 | -0.37799 | H | -5.55322 | 2.586293 | -0.43986 |
| H | -5.93834 | 2.360071 | -1.30027 | H | -5.72694 | 1.321858 | -1.67211 |
| H | -7.19267 | 1.19279 | -1.76152 | H | -7.02447 | 1.627423 | -0.5024 |
| H | -7.62754 | -0.85026 | -0.23023 | H | -6.65368 | 0.873209 | 1.997461 |
| H | -6.59131 | -1.14448 | 1.175485 | H | -5.10278 | 0.168288 | 2.465343 |
| H | -7.79041 | 0.153329 | 1.202453 | H | -5.21592 | 1.882099 | 2.039061 |
| H | -4.15852 | 1.430103 | -0.2029 | H | -4.52822 | -0.56631 | -1.2163 |
| H | -3.68207 | 0.256247 | 2.074574 | H | -3.94133 | -1.32858 | 1.726321 |
| H | -4.58056 | -1.12657 | 1.485631 | H | -4.54016 | -2.50037 | 0.588052 |
| H | -4.53208 | -1.03046 | -1.11325 | H | -4.11308 | -2.90637 | -1.36129 |
| H | 2.601477 | -5.19342 | -0.6996 | H | 3.8435 | -4.61635 | -0.40283 |
| H | 4.177012 | -4.35521 | -0.79385 | H | 5.131435 | -3.37747 | -0.41555 |
| H | 3.511565 | -4.83561 | 0.794729 | H | 4.487875 | -3.9845 | 1.138276 |
| H | -1.42207 | -0.15674 | 2.120754 | H | -1.63439 | -0.7962 | 1.848478 |
| H | -1.59428 | -2.52384 | -2.21087 | H | -0.78061 | -3.28866 | -2.3302 |
| H | 0.573383 | -2.30981 | -1.00858 | H | 1.134386 | -2.43057 | -0.99322 |
| H | 0.951129 | -0.15951 | 2.061073 | H | 0.643424 | -0.15844 | 1.970919 |
| H | 1.095647 | -1.90821 | 2.155141 | H | 1.250139 | -1.79536 | 2.172561 |
| H | 3.522743 | -2.61097 | 1.536753 | H | 3.819191 | -1.8283 | 1.776757 |
| (4*S*)-**2**-g | | | | (4*S*)-**2**-h | | | |
| O | 5.333823 | -4.29812 | 0.793041 | O | 5.310385 | -4.3424 | 0.64833 |
| C | 3.854333 | -1.51835 | -1.11882 | C | 3.594089 | -1.13736 | 1.233754 |
| C | 4.507734 | -2.71688 | -0.8516 | C | 4.242425 | -2.33974 | 1.504943 |
| C | 4.699469 | -3.1339 | 0.470254 | C | 4.672435 | -3.15232 | 0.451159 |
| C | 4.240379 | -2.33489 | 1.522826 | C | 4.460816 | -2.74407 | -0.87106 |
| C | 3.585997 | -1.14057 | 1.246342 | C | 3.817217 | -1.54205 | -1.13179 |
| C | 3.36396 | -0.7128 | -0.07625 | C | 3.353784 | -0.72023 | -0.08555 |
| C | -6.94119 | -2.69376 | 0.200579 | C | -6.93194 | -2.69065 | 0.192789 |
| C | -6.30769 | -1.57204 | -1.98771 | C | -6.29766 | -1.56102 | -1.9912 |
| C | -5.87567 | -1.98482 | -0.60128 | C | -5.86651 | -1.97785 | -0.60574 |
| C | -4.6479 | -1.78768 | -0.08432 | C | -4.63947 | -1.78085 | -0.08697 |
| C | -3.44849 | -1.13282 | -0.73474 | C | -3.44031 | -1.12236 | -0.73421 |
| O | -4.82491 | 1.153724 | 0.47255 | O | -4.81925 | 1.156121 | 0.48636 |
| C | 1.123215 | 4.764151 | -0.02956 | C | 1.147246 | 4.770695 | -0.02951 |
| O | 1.478968 | 3.482259 | 0.486083 | O | 1.496322 | 3.486691 | 0.485426 |
| O | 3.923023 | 1.260408 | -2.33977 | O | 3.918018 | 1.250823 | -2.34944 |
| C | -1.39317 | -0.17051 | 0.406261 | C | -1.38541 | -0.16197 | 0.408939 |
| C | -2.76342 | -0.09526 | 0.14062 | C | -2.75613 | -0.08794 | 0.145577 |
| C | -3.48416 | 0.992322 | 0.675441 | C | -3.478 | 0.996034 | 0.686372 |
| C | -2.82752 | 1.970034 | 1.428125 | C | -2.82197 | 1.971545 | 1.44244 |
| C | -1.45887 | 1.873573 | 1.663335 | C | -1.45277 | 1.876499 | 1.675094 |
| C | -0.72039 | 0.795152 | 1.163667 | C | -0.71324 | 0.80155 | 1.169698 |
| C | 0.7756 | 0.675735 | 1.413259 | C | 0.783422 | 0.683931 | 1.416064 |
| C | 3.065009 | 1.427626 | -1.50893 | C | 3.063508 | 1.422758 | -1.51646 |
| O | 2.257451 | 2.537037 | -1.51072 | O | 2.26204 | 2.537224 | -1.51451 |
| C | 1.316263 | 2.445344 | -0.4299 | C | 1.324686 | 2.450706 | -0.43024 |
| C | 1.610223 | 1.127525 | 0.247867 | C | 1.614329 | 1.131436 | 0.246614 |
| C | 2.657062 | 0.542688 | -0.37056 | C | 2.654282 | 0.540243 | -0.3768 |
| H | 5.619527 | -4.73819 | -0.01852 | H | 5.406525 | -4.49828 | 1.597148 |
| H | 3.730969 | -1.19332 | -2.14469 | H | 3.301157 | -0.50062 | 2.061886 |
| H | 4.875745 | -3.32826 | -1.67296 | H | 4.426208 | -2.6387 | 2.534713 |
| H | 4.417728 | -2.6564 | 2.543487 | H | 4.812934 | -3.37812 | -1.67772 |
| H | 3.270997 | -0.5125 | 2.072977 | H | 3.677192 | -1.22228 | -2.15734 |
| H | -7.2724 | -3.60482 | -0.31429 | H | -7.26287 | -3.59944 | -0.32622 |
| H | -6.58675 | -2.97542 | 1.195266 | H | -6.57759 | -2.97665 | 1.186278 |
| H | -7.83146 | -2.06311 | 0.318568 | H | -7.82235 | -2.06069 | 0.313456 |
| H | -7.22485 | -0.97187 | -1.94477 | H | -7.21608 | -0.96283 | -1.94726 |
| H | -5.55311 | -0.99865 | -2.52851 | H | -5.54354 | -0.98429 | -2.52904 |
| H | -6.54622 | -2.45894 | -2.58859 | H | -6.5336 | -2.44628 | -2.59547 |
| H | -4.46412 | -2.15389 | 0.926062 | H | -4.4561 | -2.15043 | 0.922292 |
| H | -2.71142 | -1.90818 | -0.98103 | H | -2.70265 | -1.89608 | -0.98377 |
| H | -3.73263 | -0.6738 | -1.68923 | H | -3.72448 | -0.65944 | -1.68679 |
| H | -5.20781 | 0.299651 | 0.209045 | H | -5.20081 | 0.303292 | 0.216869 |
| H | 1.22334 | 5.470286 | 0.796416 | H | 1.253765 | 5.476364 | 0.796082 |
| H | 1.788919 | 5.060526 | -0.84696 | H | 1.81267 | 5.062788 | -0.84866 |
| H | 0.084494 | 4.770862 | -0.38846 | H | 0.107708 | 4.783833 | -0.38588 |
| H | -0.832 | -1.01078 | 0.001608 | H | -0.82322 | -0.99909 | -0.00082 |
| H | -3.41178 | 2.793186 | 1.826313 | H | -3.40702 | 2.792099 | 1.844803 |
| H | -0.95851 | 2.641082 | 2.247221 | H | -0.95285 | 2.642581 | 2.261292 |
| H | 1.041529 | 1.286512 | 2.28582 | H | 1.051086 | 1.298543 | 2.28536 |
| H | 1.033477 | -0.3584 | 1.659277 | H | 1.043178 | -0.34916 | 1.665071 |
| H | 0.31485 | 2.482594 | -0.88286 | H | 0.321602 | 2.493257 | -0.87909 |
| (4*S*)-**2**-i | | | | (4*S*)-**2**-j | | | |
| O | 4.460186 | 4.622463 | -0.92901 | O | 4.404312 | 4.681495 | -0.79063 |
| C | 3.481998 | 1.640019 | 0.999626 | C | 3.069281 | 1.294357 | -1.34172 |
| C | 3.96484 | 2.914672 | 0.722113 | C | 3.535979 | 2.575565 | -1.62434 |
| C | 3.997153 | 3.382896 | -0.59611 | C | 3.941072 | 3.41511 | -0.5818 |
| C | 3.552277 | 2.559052 | -1.63563 | C | 3.888831 | 2.955674 | 0.739541 |
| C | 3.069311 | 1.288197 | -1.34876 | C | 3.42564 | 1.67558 | 1.011449 |
| C | 3.007492 | 0.805799 | -0.02768 | C | 2.988623 | 0.822725 | -0.02125 |
| C | -5.83097 | 3.404758 | -0.44103 | C | -5.81749 | 3.390386 | -0.43157 |
| C | -3.97658 | 3.620186 | 1.27655 | C | -3.96559 | 3.597838 | 1.28979 |
| C | -4.80421 | 2.700552 | 0.413589 | C | -4.79121 | 2.682378 | 0.420506 |
| C | -4.68346 | 1.365403 | 0.371572 | C | -4.66969 | 1.347518 | 0.371568 |
| C | -3.71489 | 0.489663 | 1.13803 | C | -3.70201 | 0.468371 | 1.135276 |
| O | -4.91795 | -1.95816 | 0.052339 | O | -4.90651 | -1.97481 | 0.04094 |
| C | 1.456584 | -4.90001 | -0.09314 | C | 1.508665 | -4.9027 | -0.08686 |
| O | 1.607504 | -3.57043 | -0.58838 | O | 1.644946 | -3.57229 | -0.58411 |
| O | 3.97031 | -1.13604 | 2.12533 | O | 3.96718 | -1.10505 | 2.136921 |
| C | -1.65318 | -0.26671 | -0.13911 | C | -1.64052 | -0.28519 | -0.14377 |
| C | -2.97839 | -0.4939 | 0.242877 | C | -2.96596 | -0.51265 | 0.237015 |
| C | -3.61875 | -1.64593 | -0.25036 | C | -3.60696 | -1.66273 | -0.26014 |
| C | -2.9395 | -2.53103 | -1.09079 | C | -2.92798 | -2.5458 | -1.10289 |
| C | -1.62018 | -2.27562 | -1.45357 | C | -1.60828 | -2.2902 | -1.46433 |
| C | -0.9557 | -1.13668 | -0.9842 | C | -0.9434 | -1.15305 | -0.99144 |
| C | 0.490834 | -0.8506 | -1.35903 | C | 0.503277 | -0.86617 | -1.36518 |
| C | 3.077645 | -1.38749 | 1.354447 | C | 3.08104 | -1.3685 | 1.363092 |
| O | 2.411453 | -2.58689 | 1.379478 | O | 2.429188 | -2.57642 | 1.385787 |
| C | 1.38725 | -2.58644 | 0.372725 | C | 1.40971 | -2.58931 | 0.374649 |
| C | 1.465695 | -1.22565 | -0.27851 | C | 1.475806 | -1.22885 | -0.2787 |
| C | 2.48051 | -0.5326 | 0.278418 | C | 2.478351 | -0.52263 | 0.283232 |
| H | 4.75816 | 5.074853 | -0.12871 | H | 4.406248 | 4.866795 | -1.73904 |
| H | 3.481963 | 1.278291 | 2.020627 | H | 2.795331 | 0.642226 | -2.16436 |
| H | 4.323327 | 3.546564 | 1.532049 | H | 3.598407 | 2.916786 | -2.65548 |
| H | 3.60689 | 2.923422 | -2.65584 | H | 4.220381 | 3.613042 | 1.536159 |
| H | 2.765818 | 0.645995 | -2.16879 | H | 3.409072 | 1.319163 | 2.034346 |
| H | -5.35059 | 4.119449 | -1.12202 | H | -5.33688 | 4.10915 | -1.10813 |
| H | -6.41453 | 2.702978 | -1.04269 | H | -6.39991 | 2.691405 | -1.03759 |
| H | -6.52748 | 3.98579 | 0.177592 | H | -6.51509 | 3.967624 | 0.189356 |
| H | -4.61791 | 4.168097 | 1.979049 | H | -4.60804 | 4.138903 | 1.99655 |
| H | -3.21267 | 3.103984 | 1.86055 | H | -3.20005 | 3.07953 | 1.86974 |
| H | -3.47558 | 4.377522 | 0.66034 | H | -3.4668 | 4.361052 | 0.679092 |
| H | -5.34671 | 0.826588 | -0.30582 | H | -5.33182 | 0.811806 | -0.30938 |
| H | -2.98128 | 1.094657 | 1.67556 | H | -2.96821 | 1.070912 | 1.675262 |
| H | -4.2567 | -0.06286 | 1.925723 | H | -4.24448 | -0.08651 | 1.92086 |
| H | -5.26886 | -1.31505 | 0.680974 | H | -5.25682 | -1.33404 | 0.672321 |
| H | 1.579181 | -5.5663 | -0.94864 | H | 1.641457 | -5.56906 | -0.94079 |
| H | 2.216068 | -5.12987 | 0.661456 | H | 2.268784 | -5.12208 | 0.670194 |
| H | 0.458342 | -5.04843 | 0.342057 | H | 0.511151 | -5.06237 | 0.346049 |
| H | -1.15168 | 0.623564 | 0.234935 | H | -1.1383 | 0.603242 | 0.233667 |
| H | -3.46415 | -3.40956 | -1.45166 | H | -3.45297 | -3.42306 | -1.46631 |
| H | -1.09952 | -2.9709 | -2.10609 | H | -1.08789 | -2.98401 | -2.1187 |
| H | 0.744217 | -1.41549 | -2.26546 | H | 0.760569 | -1.43837 | -2.26584 |
| H | 0.612014 | 0.209331 | -1.59995 | H | 0.622619 | 0.192149 | -1.61447 |
| H | 0.43506 | -2.76143 | 0.894396 | H | 0.457095 | -2.77402 | 0.892288 |
| (4*S*)-**2**-k | | | | (4*S*)-**2**-l | | | |
| O | -5.30971 | -4.34287 | -0.6482 | O | -5.79778 | -2.97197 | -1.56599 |
| C | -3.59385 | -1.13764 | -1.23378 | C | -3.5415 | -1.29139 | 0.812172 |
| C | -4.24205 | -2.34011 | -1.50491 | C | -4.44613 | -2.21674 | 0.302115 |
| C | -4.67195 | -3.15268 | -0.45108 | C | -4.90993 | -2.09607 | -1.01255 |
| C | -4.46042 | -2.74435 | 0.871122 | C | -4.46805 | -1.03418 | -1.80896 |
| C | -3.81696 | -1.54224 | 1.131797 | C | -3.56211 | -0.11612 | -1.29189 |
| C | -3.35361 | -0.72043 | 0.085522 | C | -3.06641 | -0.23113 | 0.020564 |
| C | 6.931306 | -2.69101 | -0.19363 | C | 4.502474 | -2.78486 | 2.696025 |
| C | 6.298292 | -1.56096 | 1.99049 | C | 2.856456 | -3.98047 | 1.18058 |
| C | 5.866362 | -1.97793 | 0.605311 | C | 3.822799 | -2.83439 | 1.348314 |
| C | 4.639091 | -1.78085 | 0.087117 | C | 4.095187 | -1.91031 | 0.415086 |
| C | 3.440432 | -1.12188 | 0.734787 | C | 3.514073 | -1.78679 | -0.97726 |
| O | 4.819189 | 1.156517 | -0.48551 | O | 5.145328 | 0.588129 | -1.54836 |
| C | -1.14813 | 4.770851 | 0.029063 | C | 0.152283 | 4.553032 | 1.344028 |
| O | -1.49674 | 3.486697 | -0.48581 | O | -0.51161 | 3.602928 | 0.511796 |
| O | -3.91802 | 1.250598 | 2.349349 | O | -2.88535 | 0.833098 | 2.877794 |
| C | 1.385453 | -0.16181 | -0.40862 | C | 1.559365 | -0.18537 | -1.23379 |
| C | 2.756127 | -0.08761 | -0.14506 | C | 2.939428 | -0.40628 | -1.2532 |
| C | 3.477969 | 0.996418 | -0.68579 | C | 3.779694 | 0.692544 | -1.51172 |
| C | 2.821971 | 1.971902 | -1.44189 | C | 3.240021 | 1.959831 | -1.74395 |
| C | 1.452804 | 1.876722 | -1.6747 | C | 1.861028 | 2.14701 | -1.71663 |
| C | 0.71331 | 0.801673 | -1.16946 | C | 0.997453 | 1.074762 | -1.46494 |
| C | -0.7833 | 0.683937 | -1.41606 | C | -0.51156 | 1.264575 | -1.42473 |
| C | -3.06363 | 1.422649 | 1.51629 | C | -2.14671 | 1.181969 | 1.990587 |
| O | -2.26242 | 2.537342 | 1.514172 | O | -1.14988 | 2.102641 | 2.194062 |
| C | -1.32501 | 2.450854 | 0.430002 | C | -0.41695 | 2.291935 | 0.973537 |
| C | -1.61438 | 1.131448 | -0.24675 | C | -1.05895 | 1.361249 | -0.02834 |
| C | -2.65425 | 0.540132 | 0.376685 | C | -2.09407 | 0.729567 | 0.563083 |
| H | -5.40606 | -4.49868 | -1.59701 | H | -6.03883 | -3.63676 | -0.9073 |
| H | -3.30102 | -0.50087 | -2.06192 | H | -3.2061 | -1.38047 | 1.838259 |
| H | -4.42581 | -2.63915 | -2.53466 | H | -4.79837 | -3.03293 | 0.929516 |
| H | -4.81249 | -3.3784 | 1.677792 | H | -4.8546 | -0.93664 | -2.81773 |
| H | -3.67699 | -1.22238 | 2.157314 | H | -3.25951 | 0.7245 | -1.90734 |
| H | 7.26216 | -3.59991 | 0.325224 | H | 5.06482 | -3.70803 | 2.888293 |
| H | 6.576528 | -2.97685 | -1.18701 | H | 5.194748 | -1.94245 | 2.774772 |
| H | 7.821845 | -2.06129 | -0.31458 | H | 3.76526 | -2.69664 | 3.504651 |
| H | 7.21612 | -0.96192 | 1.945933 | H | 2.090036 | -3.95312 | 1.965499 |
| H | 5.544087 | -0.98506 | 2.529098 | H | 2.346943 | -3.9869 | 0.215497 |
| H | 6.53555 | -2.44617 | 2.594309 | H | 3.37632 | -4.94113 | 1.28933 |
| H | 4.455194 | -2.15058 | -0.92199 | H | 4.810494 | -1.13131 | 0.680676 |
| H | 2.702671 | -1.89525 | 0.985108 | H | 4.292375 | -2.01999 | -1.72492 |
| H | 3.725302 | -0.65853 | 1.686941 | H | 2.733825 | -2.53255 | -1.14422 |
| H | 5.200797 | 0.303484 | -0.21672 | H | 5.402808 | -0.33759 | -1.45523 |
| H | -1.25472 | 5.476414 | -0.79661 | H | 0.066717 | 5.518743 | 0.843098 |
| H | -1.81378 | 5.062824 | 0.848071 | H | -0.31742 | 4.609983 | 2.331505 |
| H | -0.10866 | 4.784345 | 0.385619 | H | 1.214888 | 4.299158 | 1.463457 |
| H | 0.823296 | -0.99899 | 0.001054 | H | 0.902494 | -1.02938 | -1.03328 |
| H | 3.406985 | 2.792521 | -1.84415 | H | 3.917373 | 2.782588 | -1.94784 |
| H | 0.952855 | 2.642761 | -2.26092 | H | 1.450194 | 3.136977 | -1.89347 |
| H | -1.05085 | 1.298486 | -2.28543 | H | -0.77052 | 2.186349 | -1.96166 |
| H | -1.04295 | -0.34918 | -1.66505 | H | -1.00597 | 0.441531 | -1.94857 |
| H | -0.32195 | 2.493601 | 0.878889 | H | 0.62906 | 2.033188 | 1.193593 |
| (4*S*)-**2**-m | | | | (4*S*)-**2**-n | | | |
| O | -5.76964 | -3.05267 | -1.44034 | O | -5.62649 | -3.95901 | -0.83079 |
| C | -3.56842 | -0.10552 | -1.27214 | C | -3.78024 | -1.46014 | 1.148444 |
| C | -4.47275 | -1.03187 | -1.78545 | C | -4.53438 | -2.58972 | 0.848391 |
| C | -4.88905 | -2.10929 | -0.99702 | C | -4.88814 | -2.86751 | -0.47657 |
| C | -4.40444 | -2.24122 | 0.309691 | C | -4.48958 | -1.9979 | -1.49738 |
| C | -3.50621 | -1.31079 | 0.814206 | C | -3.73432 | -0.87303 | -1.1885 |
| C | -3.05425 | -0.23216 | 0.028572 | C | -3.34902 | -0.58668 | 0.134757 |
| C | 4.479341 | -2.77313 | 2.705756 | C | 6.388558 | -1.56264 | 2.340933 |
| C | 2.818254 | -3.96506 | 1.204007 | C | 6.345196 | -2.75501 | 0.098934 |
| C | 3.796727 | -2.82766 | 1.359726 | C | 5.610699 | -1.88018 | 1.085987 |
| C | 4.077006 | -1.91464 | 0.417963 | C | 4.355218 | -1.4206 | 0.924589 |
| C | 3.494094 | -1.79707 | -0.97414 | C | 3.410627 | -1.65809 | -0.23391 |
| O | 5.137746 | 0.564051 | -1.5686 | O | 4.842361 | 0.754958 | -1.0765 |
| C | 0.151413 | 4.563547 | 1.344196 | C | -0.761 | 4.731716 | 0.359257 |
| O | -0.51166 | 3.612181 | 0.512768 | O | -1.2508 | 3.530928 | -0.2357 |
| O | -2.86819 | 0.831871 | 2.88531 | O | -3.54017 | 1.182448 | 2.615902 |
| C | 1.548526 | -0.18554 | -1.23477 | C | 1.33564 | -0.33646 | -0.86308 |
| C | 2.927078 | -0.41501 | -1.25812 | C | 2.726682 | -0.39882 | -0.74164 |
| C | 3.773001 | 0.676975 | -1.52724 | C | 3.479413 | 0.721022 | -1.15083 |
| C | 3.240115 | 1.945974 | -1.76575 | C | 2.838245 | 1.844054 | -1.68152 |
| C | 1.86238 | 2.141785 | -1.73407 | C | 1.451849 | 1.868538 | -1.80458 |
| C | 0.993403 | 1.076518 | -1.47202 | C | 0.67647 | 0.779014 | -1.39184 |
| C | -0.51425 | 1.275768 | -1.42597 | C | -0.84117 | 0.800277 | -1.49664 |
| C | -2.13366 | 1.183732 | 1.996353 | C | -2.76145 | 1.380944 | 1.716678 |
| O | -1.13905 | 2.108356 | 2.196265 | O | -1.88319 | 2.435271 | 1.735436 |
| C | -0.41164 | 2.300976 | 0.973233 | C | -1.06695 | 2.398421 | 0.554818 |
| C | -1.05513 | 1.369564 | -0.02707 | C | -1.51927 | 1.175666 | -0.20891 |
| C | -2.08453 | 0.73288 | 0.568342 | C | -2.53358 | 0.592437 | 0.463075 |
| H | -6.03681 | -2.83849 | -2.34397 | H | -5.85353 | -4.45887 | -0.03546 |
| H | -3.28544 | 0.74555 | -1.88257 | H | -3.53034 | -1.24242 | 2.179658 |
| H | -4.86501 | -0.90891 | -2.7927 | H | -4.8544 | -3.2555 | 1.647238 |
| H | -4.74807 | -3.07391 | 0.914043 | H | -4.79199 | -2.2101 | -2.51722 |
| H | -3.15327 | -1.40697 | 1.833903 | H | -3.46659 | -0.18708 | -1.98526 |
| H | 5.031737 | -3.70068 | 2.905584 | H | 7.330965 | -1.053 | 2.103452 |
| H | 5.18104 | -1.93774 | 2.77555 | H | 5.819903 | -0.92942 | 3.026591 |
| H | 3.744553 | -2.6693 | 3.514698 | H | 6.659698 | -2.48378 | 2.872743 |
| H | 2.052598 | -3.92176 | 1.988903 | H | 6.539195 | -3.74099 | 0.540383 |
| H | 2.308015 | -3.97602 | 0.239359 | H | 5.80296 | -2.91128 | -0.83505 |
| H | 3.328069 | -4.92996 | 1.322256 | H | 7.325411 | -2.32678 | -0.14393 |
| H | 4.800708 | -1.14069 | 0.675523 | H | 3.935424 | -0.81473 | 1.728039 |
| H | 4.269436 | -2.04004 | -1.72173 | H | 3.93706 | -2.14527 | -1.06337 |
| H | 2.708967 | -2.53915 | -1.13439 | H | 2.631015 | -2.36301 | 0.083236 |
| H | 5.390188 | -0.3622 | -1.46728 | H | 5.140744 | 0.068642 | -0.45559 |
| H | 0.061819 | 5.529466 | 0.844338 | H | -0.89605 | 5.522731 | -0.3803 |
| H | -0.31599 | 4.618208 | 2.332877 | H | -1.32033 | 4.98029 | 1.267224 |
| H | 1.215063 | 4.312767 | 1.460909 | H | 0.306582 | 4.643621 | 0.605035 |
| H | 0.887365 | -1.02402 | -1.02535 | H | 0.749077 | -1.19652 | -0.54544 |
| H | 3.92166 | 2.763312 | -1.97731 | H | 3.447318 | 2.686135 | -1.99368 |
| H | 1.45694 | 3.133187 | -1.91543 | H | 0.96422 | 2.748027 | -2.21568 |
| H | -0.76912 | 2.201598 | -1.95777 | H | -1.13631 | 1.526848 | -2.26485 |
| H | -1.01628 | 0.457931 | -1.95098 | H | -1.20659 | -0.1768 | -1.82491 |
| H | 0.636042 | 2.044918 | 1.188576 | H | -0.02407 | 2.330659 | 0.897415 |
| (4*S*)-**2**-o | | | | (4*S*)-**2**-p | | | |
| O | -5.64571 | -3.97171 | -0.68714 | O | 5.58059 | 2.634901 | -1.41564 |
| C | -3.7574 | -0.84689 | -1.16741 | C | 3.137472 | -0.12003 | -1.46939 |
| C | -4.5215 | -1.97061 | -1.47232 | C | 4.062765 | 0.813625 | -1.92961 |
| C | -4.90021 | -2.85256 | -0.45522 | C | 4.669454 | 1.695237 | -1.02944 |
| C | -4.52006 | -2.59178 | 0.866524 | C | 4.353411 | 1.623066 | 0.332387 |
| C | -3.76123 | -1.46742 | 1.161504 | C | 3.432743 | 0.686574 | 0.782452 |
| C | -3.34796 | -0.57911 | 0.149052 | C | 2.790756 | -0.19369 | -0.11057 |
| C | 6.40372 | -1.51522 | 2.331041 | C | -0.9197 | 4.804478 | 0.790648 |
| C | 6.312903 | -2.78306 | 0.132395 | C | -2.20817 | 3.436523 | 2.49576 |
| C | 5.604408 | -1.86418 | 1.09811 | C | -1.95586 | 3.734481 | 1.038734 |
| C | 4.353295 | -1.39244 | 0.937848 | C | -2.57207 | 3.130752 | 0.010738 |
| C | 3.388929 | -1.65761 | -0.19824 | C | -3.63895 | 2.068904 | 0.058367 |
| O | 4.835642 | 0.71444 | -1.13042 | O | -5.55513 | -0.00291 | -0.6603 |
| C | -0.73418 | 4.726443 | 0.366681 | C | -0.71432 | -4.88148 | 0.774458 |
| O | -1.23591 | 3.52978 | -0.22651 | O | -0.05419 | -3.85147 | 0.040345 |
| O | -3.5092 | 1.182425 | 2.638134 | O | 2.838193 | -1.74862 | 2.516683 |
| C | 1.321288 | -0.33653 | -0.85116 | C | -1.89356 | 0.368751 | -0.7243 |
| C | 2.712629 | -0.40825 | -0.7398 | C | -3.23054 | 0.716443 | -0.51653 |
| C | 3.471923 | 0.691247 | -1.19056 | C | -4.21768 | -0.23551 | -0.8393 |
| C | 2.836117 | 1.803863 | -1.74878 | C | -3.86155 | -1.479 | -1.36498 |
| C | 1.448825 | 1.838079 | -1.85952 | C | -2.52024 | -1.79242 | -1.56768 |
| C | 0.667487 | 0.768573 | -1.40801 | C | -1.51564 | -0.87238 | -1.25023 |
| C | -0.85077 | 0.800657 | -1.49967 | C | -0.04419 | -1.20585 | -1.44878 |
| C | -2.7372 | 1.380094 | 1.733406 | C | 1.971942 | -1.87262 | 1.687237 |
| O | -1.85298 | 2.430171 | 1.747396 | O | 0.91962 | -2.73767 | 1.856893 |
| C | -1.04882 | 2.394041 | 0.55863 | C | 0.036461 | -2.64357 | 0.728474 |
| C | -1.51425 | 1.176038 | -0.20464 | C | 0.648231 | -1.59803 | -0.17421 |
| C | -2.52451 | 0.595176 | 0.474944 | C | 1.799712 | -1.16423 | 0.378421 |
| H | -5.85342 | -4.02564 | -1.62934 | H | 5.721347 | 2.568024 | -2.36933 |
| H | -3.5047 | -0.15276 | -1.96202 | H | 2.704354 | -0.82174 | -2.17443 |
| H | -4.83504 | -2.15337 | -2.49793 | H | 4.323061 | 0.846852 | -2.9854 |
| H | -4.83353 | -3.27719 | 1.646689 | H | 4.843735 | 2.303293 | 1.020575 |
| H | -3.48959 | -1.26107 | 2.189697 | H | 3.210057 | 0.621635 | 1.840695 |
| H | 7.350631 | -1.02918 | 2.063817 | H | 0.046916 | 4.524984 | 1.229093 |
| H | 5.853897 | -0.84978 | 3.001339 | H | -0.76882 | 4.989119 | -0.27618 |
| H | 6.66773 | -2.42148 | 2.891241 | H | -1.21248 | 5.751496 | 1.262649 |
| H | 6.49708 | -3.75663 | 0.604537 | H | -2.56307 | 4.334362 | 3.018008 |
| H | 5.756806 | -2.96185 | -0.78934 | H | -2.93821 | 2.641952 | 2.659439 |
| H | 7.296535 | -2.37878 | -0.13627 | H | -1.27544 | 3.139074 | 2.991271 |
| H | 3.953155 | -0.75362 | 1.725643 | H | -2.28468 | 3.432457 | -0.99637 |
| H | 3.896963 | -2.18009 | -1.01765 | H | -4.00524 | 1.934112 | 1.084395 |
| H | 2.605563 | -2.3411 | 0.154553 | H | -4.49944 | 2.448601 | -0.51878 |
| H | 5.134094 | 0.04257 | -0.49378 | H | -5.6839 | 0.873793 | -0.27771 |
| H | -0.87284 | 5.520244 | -0.36924 | H | -0.78298 | -5.74316 | 0.108343 |
| H | -1.28291 | 4.975232 | 1.281048 | H | -0.14671 | -5.15556 | 1.669887 |
| H | 0.335422 | 4.632532 | 0.601272 | H | -1.72612 | -4.5698 | 1.069445 |
| H | 0.729627 | -1.18048 | -0.50129 | H | -1.12769 | 1.095511 | -0.46737 |
| H | 3.449939 | 2.630541 | -2.09134 | H | -4.64952 | -2.18412 | -1.60834 |
| H | 0.966037 | 2.710071 | -2.29193 | H | -2.25182 | -2.76432 | -1.9723 |
| H | -1.14722 | 1.531724 | -2.26301 | H | 0.040782 | -2.03913 | -2.15826 |
| H | -1.22691 | -0.17284 | -1.82729 | H | 0.476695 | -0.35287 | -1.89336 |
| H | -0.00274 | 2.320711 | 0.890114 | H | -0.94647 | -2.34872 | 1.124124 |

**
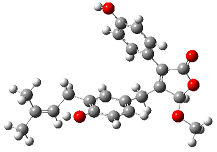

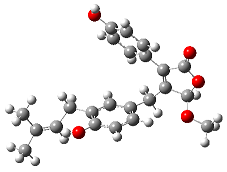

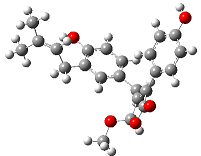
**

**a b c**

**
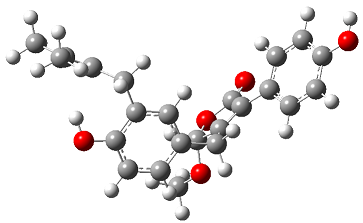

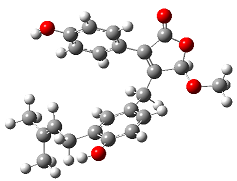

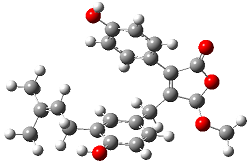
**

**d e f**

**
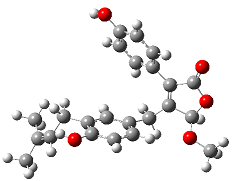

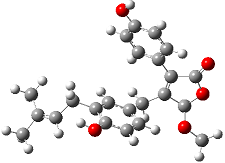

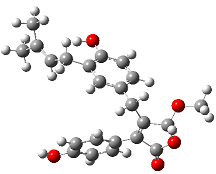
**

**g h i**

**
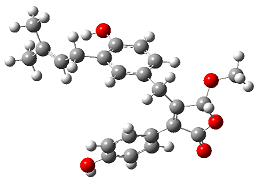

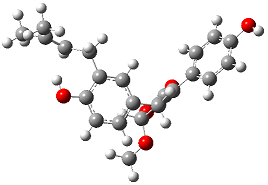

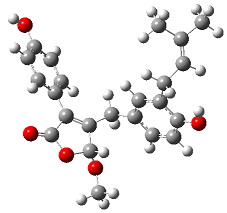
**

**j k l**

**
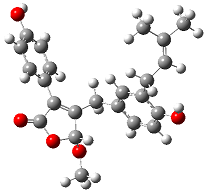

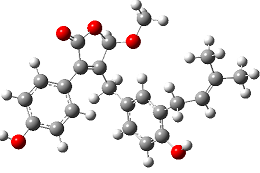

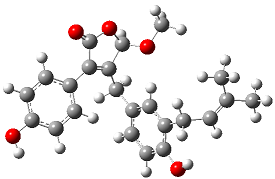
**

**m n o**

**
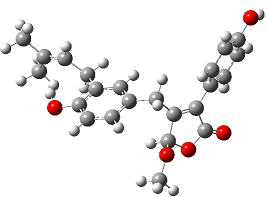

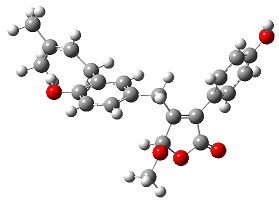

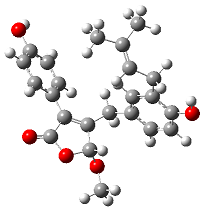
**

**p q r**

**Figure S54**. Optimized geometries of predominant conformers for compound (4*R*)-**2** at the B3LYP/6-31G(d,p) level in the gas phase.

**Table S7**. Important thermodynamic parameters (a.u.) and Boltzmann distributions of the optimized compound (4*R*)-**2** at B3LYP/6-31G(d,p) level in the gas phase.

| Conformations | E+ZPE | Gibbs | % |
| --- | --- | --- | --- |
| (4*R*)-**2**-a | -1266.615227 | -1266.675591 | 0.2 |
| (4*R*)-**2**-b | -1266.615930 | -1266.676173 | 0.3 |
| (4*R*)-**2**-c | -1266.617412 | -1266.677047 | 0.7 |
| (4*R*)-**2**-d | -1266.677047 | -1266.680681 | 32 |
| (4*R*)-**2**-e | -1266.611405 | -1266.672185 | 0 |
| (4*R*)-**2**-f | -1266.612201 | -1266.672829 | 0 |
| (4*R*)-**2**-g | -1266.611001 | -1266.672054 | 0 |
| (4*R*)-**2**-h | -1266.611686 | -1266.672417 | 0 |
| (4*R*)-**2**-i | -1266.615348 | -1266.675621 | 0.2 |
| (4*R*)-**2**-j | -1266.615987 | -1266.675986 | 0.2 |
| (4*R*)-**2**-k | -1266.619396 | -1266.680742 | 34.1 |
| (4*R*)-**2**-l | -1266.615334 | -1266.676896 | 0.6 |
| (4*R*)-**2**-m | -1266.615055 | -1266.676659 | 0.5 |
| (4*R*)-**2**-n | -1266.613243 | -1266.673908 | 0 |
| (4*R*)-**2**-o | -1266.613083 | -1266.673730 | 17.5 |
| (4*R*)-**2**-p | -1266.619599 | -1266.680113 | 12.9 |
| (4*R*)-**2**-q | -1266.619292 | -1266.679824 | 0.8 |
| (4*R*)-**2**-r | -1266.615355 | -1266.677201 | 0.2 |

E+ZPE, G: total energy with zero point energy (ZPE) and Gibbs free energy in the gas phase at B3LYP/6-31G(d,p) level., %: Boltzmann distributions, using the relative Gibbs free energies as weighting factors.

**Table S8.** Optimized Z-matrixes of compound (4*R*)-**2** in the gas phase (Å) at B3LYP/6-31G(d,p) level.

| (4*R*)-**2**-a | | | | (4*R*)-**2**-b | | | |
| --- | --- | --- | --- | --- | --- | --- | --- |
| C | 2.890783 | 0.258555 | -0.26602 | C | 2.881117 | 0.286069 | -0.26588 |
| C | 2.502491 | -0.91685 | -0.80855 | C | 2.51782 | -0.89768 | -0.80781 |
| C | 3.624565 | -1.91691 | -0.65402 | C | 3.658819 | -1.87509 | -0.64729 |
| O | 4.674884 | -1.22281 | 0.024085 | O | 4.691269 | -1.16012 | 0.037624 |
| C | 4.272279 | 0.064346 | 0.28662 | C | 4.261733 | 0.118598 | 0.296659 |
| C | 1.208394 | -1.36924 | -1.43745 | C | 1.234503 | -1.37596 | -1.43989 |
| C | -0.01717 | -1.25253 | -0.54124 | C | 0.005266 | -1.27609 | -0.54665 |
| C | -0.00995 | -1.776 | 0.75866 | C | 0.012359 | -1.81271 | 0.747902 |
| C | -1.1512 | -1.72181 | 1.549891 | C | -1.12997 | -1.76893 | 1.53825 |
| C | -2.32469 | -1.13621 | 1.062776 | C | -2.30389 | -1.17983 | 1.056055 |
| C | -2.35713 | -0.58858 | -0.23332 | C | -2.33633 | -0.6198 | -0.23473 |
| C | -1.19616 | -0.66959 | -1.01075 | C | -1.17455 | -0.69143 | -1.01178 |
| O | 4.988857 | 0.844423 | 0.861617 | O | 4.958434 | 0.912397 | 0.878026 |
| O | 3.17526 | -3.01324 | 0.079601 | O | 3.228856 | -2.98124 | 0.082817 |
| C | 4.135513 | -4.06104 | 0.203257 | C | 4.209332 | -4.00994 | 0.207947 |
| O | -3.40318 | -1.10415 | 1.901512 | O | -3.38261 | -1.15715 | 1.894845 |
| C | -3.59767 | 0.119021 | -0.75441 | C | -3.57602 | 0.093411 | -0.75023 |
| C | -4.85369 | -0.72403 | -0.70385 | C | -4.83484 | -0.74528 | -0.69969 |
| C | -6.07116 | -0.38468 | -0.23932 | C | -6.05114 | -0.40121 | -0.23548 |
| C | -7.21752 | -1.36595 | -0.30302 | C | -7.20157 | -1.37761 | -0.30033 |
| C | -6.43106 | 0.960182 | 0.344571 | C | -6.40559 | 0.945075 | 0.348332 |
| C | 2.204473 | 1.555033 | -0.16648 | C | 2.169181 | 1.569033 | -0.17315 |
| C | 2.459418 | 2.426447 | 0.911627 | C | 1.24921 | 1.971818 | -1.15909 |
| C | 1.816693 | 3.652736 | 1.014747 | C | 0.581604 | 3.18615 | -1.07851 |
| C | 0.906683 | 4.05094 | 0.029895 | C | 0.815391 | 4.036077 | 0.008282 |
| C | 0.649806 | 3.207989 | -1.05662 | C | 1.730215 | 3.659628 | 0.996545 |
| C | 1.290392 | 1.976494 | -1.14574 | C | 2.402392 | 2.445438 | 0.901714 |
| O | 0.307246 | 5.267946 | 0.177659 | O | 0.129141 | 5.21504 | 0.045003 |
| H | 4.040248 | -2.25085 | -1.62007 | H | 4.086849 | -2.19901 | -1.61132 |
| H | 1.02789 | -0.81953 | -2.36884 | H | 1.047236 | -0.83264 | -2.3736 |
| H | 1.335986 | -2.4184 | -1.73042 | H | 1.38173 | -2.42375 | -1.72818 |
| H | 0.894332 | -2.23727 | 1.144443 | H | 0.917721 | -2.27477 | 1.130405 |
| H | -1.15966 | -2.13335 | 2.553953 | H | -1.13886 | -2.19067 | 2.538112 |
| H | -1.21991 | -0.25346 | -2.01643 | H | -1.19794 | -0.26438 | -2.01273 |
| H | 3.637284 | -4.87936 | 0.725165 | H | 3.725548 | -4.8393 | 0.725997 |
| H | 4.467831 | -4.41024 | -0.78471 | H | 4.552476 | -4.35009 | -0.77946 |
| H | 5.006564 | -3.73277 | 0.779553 | H | 5.07137 | -3.66569 | 0.788486 |
| H | -4.20502 | -0.94685 | 1.375479 | H | -4.18369 | -0.98839 | 1.370953 |
| H | -3.74244 | 1.05495 | -0.20162 | H | -3.71544 | 1.028248 | -0.19439 |
| H | -3.40951 | 0.410477 | -1.79633 | H | -3.38932 | 0.38841 | -1.79129 |
| H | -4.73631 | -1.72617 | -1.11719 | H | -4.72169 | -1.74751 | -1.1141 |
| H | -6.91312 | -2.32477 | -0.73025 | H | -6.90057 | -2.33814 | -0.72617 |
| H | -7.63437 | -1.55148 | 0.695088 | H | -7.62117 | -1.56043 | 0.697166 |
| H | -8.03842 | -0.96591 | -0.91225 | H | -8.01959 | -0.9746 | -0.91149 |
| H | -5.57851 | 1.633012 | 0.450181 | H | -5.55059 | 1.614976 | 0.452413 |
| H | -7.17478 | 1.460522 | -0.28896 | H | -7.1479 | 1.447873 | -0.28487 |
| H | -6.89513 | 0.84214 | 1.331458 | H | -6.86936 | 0.829062 | 1.335657 |
| H | 3.17752 | 2.137331 | 1.668034 | H | 1.069312 | 1.336151 | -2.01805 |
| H | 2.010877 | 4.317564 | 1.849518 | H | -0.11881 | 3.495638 | -1.84682 |
| H | -0.04667 | 3.51656 | -1.83348 | H | 1.921197 | 4.318588 | 1.840909 |
| H | 1.091296 | 1.347823 | -2.00558 | H | 3.123643 | 2.174902 | 1.661916 |
| H | -0.28507 | 5.422592 | -0.56996 | H | 0.386848 | 5.705713 | 0.836766 |
| (4*R*)-**2**-c | | | | (4*R*)-**2**-d | | | |
| C | -2.55561 | -0.79893 | -0.18595 | C | -2.53384 | 0.592381 | -0.46293 |
| C | -1.77233 | -1.45484 | 0.700451 | C | -1.51947 | 1.175598 | 0.208974 |
| C | -1.76386 | -2.9238 | 0.345724 | C | -1.06741 | 2.398527 | -0.55463 |
| O | -2.57783 | -3.04488 | -0.82543 | O | -1.88386 | 2.435515 | -1.73507 |
| C | -3.06506 | -1.80862 | -1.17231 | C | -2.76202 | 1.381103 | -1.71635 |
| C | -1.07981 | -0.97153 | 1.944927 | C | -0.8411 | 0.800035 | 1.496527 |
| C | 0.302432 | -0.33905 | 1.782947 | C | 0.676528 | 0.779044 | 1.391515 |
| C | 0.725987 | 0.621322 | 2.711333 | C | 1.451743 | 1.868882 | 1.803737 |
| C | 1.987966 | 1.199148 | 2.620371 | C | 2.838125 | 1.84464 | 1.680488 |
| C | 2.86136 | 0.82247 | 1.595893 | C | 3.479456 | 0.721549 | 1.150119 |
| C | 2.468789 | -0.1494 | 0.655186 | C | 2.726893 | -0.3986 | 0.74144 |
| C | 1.189811 | -0.70596 | 0.765493 | C | 1.335857 | -0.33648 | 0.863074 |
| O | -3.79206 | -1.66147 | -2.12254 | O | -3.54091 | 1.182723 | -2.61544 |
| O | -0.46834 | -3.37701 | 0.113115 | O | -1.2512 | 3.530892 | 0.236137 |
| C | -0.37935 | -4.78145 | -0.12328 | C | -0.76165 | 4.731823 | -0.35874 |
| O | 4.089352 | 1.420437 | 1.569154 | O | 4.842377 | 0.755769 | 1.075568 |
| C | 3.426965 | -0.62115 | -0.42785 | C | 3.410994 | -1.65795 | 0.234109 |
| C | 3.994216 | 0.49517 | -1.27779 | C | 4.355586 | -1.42076 | -0.92445 |
| C | 5.277435 | 0.714464 | -1.62247 | C | 5.611121 | -1.88026 | -1.08566 |
| C | 5.644334 | 1.873843 | -2.5182 | C | 6.388952 | -1.56309 | -2.34072 |
| C | 6.444497 | -0.14781 | -1.20579 | C | 6.345737 | -2.7546 | -0.09827 |
| C | -2.93816 | 0.618104 | -0.26196 | C | -3.34907 | -0.58689 | -0.13469 |
| C | -4.18069 | 0.997657 | -0.81187 | C | -3.73409 | -0.87356 | 1.188578 |
| C | -4.57171 | 2.328386 | -0.8572 | C | -4.48915 | -1.99858 | 1.49737 |
| C | -3.72241 | 3.32501 | -0.36277 | C | -4.8878 | -2.86804 | 0.476461 |
| C | -2.47855 | 2.972152 | 0.170424 | C | -4.53432 | -2.58993 | -0.84851 |
| C | -2.09491 | 1.635317 | 0.218536 | C | -3.78038 | -1.46021 | -1.14847 |
| O | -4.15585 | 4.616345 | -0.4343 | O | -5.62596 | -3.9597 | 0.830596 |
| H | -2.24291 | -3.53835 | 1.127391 | H | -0.02458 | 2.330917 | -0.89741 |
| H | -1.73209 | -0.23906 | 2.436392 | H | -1.13627 | 1.526378 | 2.264946 |
| H | -0.9972 | -1.82277 | 2.635098 | H | -1.2063 | -0.17718 | 1.82462 |
| H | 0.057278 | 0.929115 | 3.511827 | H | 0.963988 | 2.748423 | 2.214586 |
| H | 2.315924 | 1.952558 | 3.329148 | H | 3.447064 | 2.686963 | 1.992267 |
| H | 0.886479 | -1.45869 | 0.044505 | H | 0.749427 | -1.19679 | 0.54584 |
| H | 0.683201 | -5.01669 | -0.20064 | H | -0.89672 | 5.522729 | 0.380934 |
| H | -0.81578 | -5.34942 | 0.710325 | H | 0.30591 | 4.643909 | -0.60467 |
| H | -0.88731 | -5.05953 | -1.05239 | H | -1.32114 | 4.980436 | -1.2666 |
| H | 4.475125 | 1.304749 | 0.684029 | H | 5.140849 | 0.069154 | 0.455032 |
| H | 4.235748 | -1.20343 | 0.029901 | H | 3.937462 | -2.14481 | 1.063732 |
| H | 2.883812 | -1.31908 | -1.07786 | H | 2.631461 | -2.36305 | -0.08283 |
| H | 3.245395 | 1.186406 | -1.66548 | H | 3.935741 | -0.81523 | -1.72813 |
| H | 4.771097 | 2.469758 | -2.79545 | H | 5.820327 | -0.92997 | -3.0265 |
| H | 6.371863 | 2.53522 | -2.03072 | H | 7.331424 | -1.05349 | -2.10341 |
| H | 6.119689 | 1.516468 | -3.44085 | H | 6.659964 | -2.48438 | -2.87233 |
| H | 6.175123 | -0.93417 | -0.49901 | H | 5.803294 | -2.91104 | 0.835572 |
| H | 6.892304 | -0.62822 | -2.08519 | H | 6.540374 | -3.74053 | -0.53954 |
| H | 7.234401 | 0.462556 | -0.75092 | H | 7.325665 | -2.32585 | 0.144842 |
| H | -4.8368 | 0.236707 | -1.21486 | H | -3.4663 | -0.18773 | 1.985424 |
| H | -5.53015 | 2.616158 | -1.27589 | H | -4.79135 | -2.21103 | 2.51723 |
| H | -1.80443 | 3.742283 | 0.539625 | H | -4.85442 | -3.25559 | -1.64743 |
| H | -1.11417 | 1.387785 | 0.607108 | H | -3.53071 | -1.24224 | -2.17969 |
| H | -3.4769 | 5.199198 | -0.0692 | H | -5.853 | -4.45948 | 0.035228 |
| (4*R*)-**2**-e | | | | (4*R*)-**2**-f | | | |
| C | 2.384799 | 0.740531 | -0.42398 | C | 2.370979 | 0.761358 | -0.4191 |
| C | 2.222228 | -0.47996 | -0.98037 | C | 2.228128 | -0.45981 | -0.97971 |
| C | 3.583255 | -1.10492 | -1.1829 | C | 3.59887 | -1.06452 | -1.17767 |
| O | 4.526693 | -0.15614 | -0.67645 | O | 4.52628 | -0.1048 | -0.661 |
| C | 3.853758 | 0.944173 | -0.2025 | C | 3.835212 | 0.983346 | -0.1862 |
| C | 0.989855 | -1.2741 | -1.33823 | C | 1.008172 | -1.26984 | -1.34483 |
| C | 0.063441 | -1.56174 | -0.16363 | C | 0.082257 | -1.5733 | -0.17366 |
| C | 0.546753 | -2.19232 | 0.990763 | C | 0.5685 | -2.21205 | 0.975012 |
| C | -0.30883 | -2.48626 | 2.045486 | C | -0.28594 | -2.51977 | 2.026819 |
| C | -1.66396 | -2.15238 | 1.966875 | C | -1.64262 | -2.1915 | 1.950796 |
| C | -2.17644 | -1.50888 | 0.826801 | C | -2.15792 | -1.53921 | 0.816999 |
| C | -1.29284 | -1.23475 | -0.22301 | C | -1.27546 | -1.25144 | -0.23005 |
| O | 4.435988 | 1.879766 | 0.285515 | O | 4.401331 | 1.923785 | 0.312213 |
| O | 3.652556 | -2.31557 | -0.49702 | O | 3.68277 | -2.2775 | -0.49792 |
| C | 4.871455 | -3.0286 | -0.70025 | C | 4.911835 | -2.97275 | -0.70128 |
| O | -2.44246 | -2.48509 | 3.044268 | O | -2.4201 | -2.53757 | 3.024946 |
| C | -3.65437 | -1.14184 | 0.756986 | C | -3.63737 | -1.17744 | 0.749271 |
| C | -4.00208 | -0.10875 | -0.28201 | C | -3.98608 | -0.12397 | -0.26907 |
| C | -4.84931 | -0.2213 | -1.31644 | C | -4.82246 | -0.22284 | -1.31376 |
| C | -5.09724 | 0.950176 | -2.23694 | C | -5.07621 | 0.967027 | -2.20872 |
| C | -5.62878 | -1.46395 | -1.66885 | C | -5.58365 | -1.46633 | -1.70172 |
| C | 1.407945 | 1.775795 | -0.05402 | C | 1.377266 | 1.78157 | -0.05311 |
| C | 1.636846 | 2.623413 | 1.048214 | C | 0.198831 | 1.959031 | -0.80085 |
| C | 0.722018 | 3.605291 | 1.404127 | C | -0.73656 | 2.928873 | -0.46544 |
| C | -0.44582 | 3.779234 | 0.653934 | C | -0.51597 | 3.75103 | 0.645421 |
| C | -0.68476 | 2.959868 | -0.45474 | C | 0.652436 | 3.597738 | 1.398187 |
| C | 0.232596 | 1.970756 | -0.79643 | C | 1.589264 | 2.630944 | 1.047153 |
| O | -1.30634 | 4.763729 | 1.044282 | O | -1.46814 | 4.683869 | 0.937499 |
| H | 3.831064 | -1.25747 | -2.24732 | H | 3.854445 | -1.20737 | -2.24157 |
| H | 0.424719 | -0.76479 | -2.12701 | H | 0.438819 | -0.76564 | -2.13374 |
| H | 1.323445 | -2.22397 | -1.77245 | H | 1.356349 | -2.21362 | -1.78074 |
| H | 1.598913 | -2.4522 | 1.05889 | H | 1.621925 | -2.46745 | 1.041041 |
| H | 0.051519 | -2.97796 | 2.943163 | H | 0.076424 | -3.01868 | 2.919742 |
| H | -1.68909 | -0.74936 | -1.11051 | H | -1.67427 | -0.75799 | -1.11184 |
| H | 4.764208 | -3.98205 | -0.18094 | H | 4.815795 | -3.93067 | -0.18805 |
| H | 5.045346 | -3.21562 | -1.76944 | H | 5.091739 | -3.15095 | -1.77096 |
| H | 5.723967 | -2.4788 | -0.28859 | H | 5.755541 | -2.41402 | -0.28347 |
| H | -3.35521 | -2.21932 | 2.878544 | H | -3.33512 | -2.28116 | 2.856649 |
| H | -4.25639 | -2.04935 | 0.619517 | H | -4.23433 | -2.08492 | 0.590792 |
| H | -3.95785 | -0.72744 | 1.734065 | H | -3.94631 | -0.78486 | 1.733581 |
| H | -3.49425 | 0.84616 | -0.14549 | H | -3.49168 | 0.833496 | -0.10552 |
| H | -4.51689 | 1.829975 | -1.94549 | H | -4.51314 | 1.847891 | -1.8889 |
| H | -4.8402 | 0.699199 | -3.27433 | H | -4.80371 | 0.744277 | -3.24872 |
| H | -6.15884 | 1.230361 | -2.24066 | H | -6.14186 | 1.231273 | -2.21847 |
| H | -5.40025 | -2.31769 | -1.02881 | H | -5.34968 | -2.33269 | -1.08068 |
| H | -6.70821 | -1.27457 | -1.60458 | H | -6.66585 | -1.29164 | -1.64179 |
| H | -5.42815 | -1.76052 | -2.70615 | H | -5.3716 | -1.73539 | -2.74428 |
| H | 2.547786 | 2.511993 | 1.622356 | H | 0.021833 | 1.34645 | -1.67724 |
| H | 0.898425 | 4.251662 | 2.257117 | H | -1.6356 | 3.071405 | -1.05561 |
| H | -1.58058 | 3.101704 | -1.05589 | H | 0.833599 | 4.238886 | 2.258242 |
| H | 0.039849 | 1.361384 | -1.67176 | H | 2.499807 | 2.536682 | 1.624881 |
| H | -2.05809 | 4.788056 | 0.437676 | H | -1.18938 | 5.187522 | 1.713826 |
| (4*R*)-**2**-g | | | | (4*R*)-**2**-h | | | |
| C | 2.740759 | 0.150375 | -0.46153 | C | 2.73402 | 0.176677 | -0.46247 |
| C | 2.178929 | -0.99461 | -0.90752 | C | 2.192891 | -0.9774 | -0.911 |
| C | 3.239084 | -2.07084 | -0.94879 | C | 3.271622 | -2.03508 | -0.95056 |
| O | 4.43869 | -1.45395 | -0.47232 | O | 4.458711 | -1.3985 | -0.46731 |
| C | 4.18017 | -0.1416 | -0.15781 | C | 4.176221 | -0.09173 | -0.15184 |
| C | 0.760555 | -1.34929 | -1.2802 | C | 0.781238 | -1.35545 | -1.28659 |
| C | -0.25266 | -1.17872 | -0.1558 | C | -0.23499 | -1.20211 | -0.16231 |
| C | -0.04628 | -1.78213 | 1.092803 | C | -0.02751 | -1.81826 | 1.079909 |
| C | -0.993 | -1.66075 | 2.102371 | C | -0.97512 | -1.71011 | 2.090218 |
| C | -2.16886 | -0.93652 | 1.882534 | C | -2.15233 | -0.98585 | 1.877567 |
| C | -2.40355 | -0.31769 | 0.643803 | C | -2.38763 | -0.35362 | 0.64575 |
| C | -1.43146 | -0.45781 | -0.35374 | C | -1.41525 | -0.4816 | -0.35308 |
| O | 5.038157 | 0.585581 | 0.275131 | O | 5.019487 | 0.648855 | 0.288195 |
| O | 2.855846 | -3.14082 | -0.14293 | O | 2.904653 | -3.11398 | -0.14937 |
| C | 3.749804 | -4.25182 | -0.19036 | C | 3.817003 | -4.20981 | -0.19853 |
| O | -3.05763 | -0.87375 | 2.924644 | O | -3.04151 | -0.9353 | 2.920107 |
| C | -3.68223 | 0.461984 | 0.384409 | C | -3.66631 | 0.428706 | 0.39517 |
| C | -4.87685 | -0.43483 | 0.137109 | C | -4.85942 | -0.46545 | 0.131399 |
| C | -5.7783 | -0.36072 | -0.85331 | C | -5.76355 | -0.37126 | -0.8549 |
| C | -6.90583 | -1.36123 | -0.94247 | C | -6.88889 | -1.37238 | -0.96317 |
| C | -5.78282 | 0.680871 | -1.94457 | C | -5.7734 | 0.6947 | -1.92233 |
| C | 2.176847 | 1.493725 | -0.26238 | C | 2.146245 | 1.509834 | -0.26495 |
| C | 2.657483 | 2.334053 | 0.761636 | C | 1.119234 | 1.990361 | -1.09827 |
| C | 2.13103 | 3.603645 | 0.958242 | C | 0.56688 | 3.251298 | -0.91935 |
| C | 1.114513 | 4.077143 | 0.121969 | C | 1.030972 | 4.071568 | 0.115049 |
| C | 0.634516 | 3.265935 | -0.91181 | C | 2.05704 | 3.618286 | 0.950042 |
| C | 1.160804 | 1.991147 | -1.09403 | C | 2.611273 | 2.357098 | 0.756321 |
| O | 0.636808 | 5.33365 | 0.356647 | O | 0.451426 | 5.299076 | 0.253894 |
| H | 3.452242 | -2.42437 | -1.97211 | H | 3.495196 | -2.38152 | -1.97403 |
| H | 0.437893 | -0.76341 | -2.14824 | H | 0.450086 | -0.77354 | -2.15397 |
| H | 0.756729 | -2.39608 | -1.60661 | H | 0.795081 | -2.40171 | -1.61423 |
| H | 0.864028 | -2.34712 | 1.269402 | H | 0.884455 | -2.38231 | 1.251069 |
| H | -0.84255 | -2.12111 | 3.073388 | H | -0.82439 | -2.18109 | 3.056122 |
| H | -1.61232 | 0.014236 | -1.31731 | H | -1.59635 | 0.001505 | -1.31094 |
| H | 3.297372 | -5.04089 | 0.412038 | H | 3.375748 | -5.00884 | 0.399001 |
| H | 3.877769 | -4.61102 | -1.22135 | H | 3.954255 | -4.56243 | -1.23058 |
| H | 4.729009 | -3.98874 | 0.222497 | H | 4.790424 | -3.93242 | 0.218687 |
| H | -3.79192 | -0.29276 | 2.690059 | H | -3.78039 | -0.35926 | 2.687554 |
| H | -3.8896 | 1.127221 | 1.241033 | H | -3.87571 | 1.08086 | 1.261247 |
| H | -3.51808 | 1.137065 | -0.45882 | H | -3.50062 | 1.117105 | -0.43674 |
| H | -4.99322 | -1.24485 | 0.857824 | H | -4.97237 | -1.2916 | 0.834246 |
| H | -6.86785 | -2.09437 | -0.13246 | H | -6.84697 | -2.12282 | -0.16931 |
| H | -7.88224 | -0.86052 | -0.90397 | H | -7.86643 | -0.87506 | -0.91148 |
| H | -6.87399 | -1.90543 | -1.89556 | H | -6.85806 | -1.89558 | -1.92798 |
| H | -4.94443 | 1.377485 | -1.88748 | H | -4.93683 | 1.392199 | -1.85202 |
| H | -5.75875 | 0.203885 | -2.93284 | H | -5.75125 | 0.240227 | -2.9212 |
| H | -6.70786 | 1.271041 | -1.90925 | H | -6.69977 | 1.281562 | -1.87088 |
| H | 3.45876 | 1.985531 | 1.400604 | H | 0.762636 | 1.378504 | -1.9188 |
| H | 2.499148 | 4.245167 | 1.751639 | H | -0.21715 | 3.620596 | -1.57176 |
| H | -0.14464 | 3.633635 | -1.5763 | H | 2.42744 | 4.254489 | 1.751135 |
| H | 0.790378 | 1.387589 | -1.91458 | H | 3.419443 | 2.026237 | 1.395933 |
| H | -0.04678 | 5.540137 | -0.29432 | H | 0.867968 | 5.763085 | 0.992281 |
| (4*R*)-**2**-i | | | | (4*R*)-**2**-j | | | |
| C | -2.73935 | 0.629326 | 0.209863 | C | -2.74364 | 0.622517 | 0.225076 |
| C | -2.66502 | -0.59486 | 0.777693 | C | -2.66459 | -0.60909 | 0.777072 |
| C | -3.9727 | -1.31625 | 0.547892 | C | -3.9747 | -1.32702 | 0.551383 |
| O | -4.78838 | -0.41424 | -0.20461 | O | -4.79929 | -0.41365 | -0.17753 |
| C | -4.08694 | 0.743661 | -0.43929 | C | -4.09962 | 0.746011 | -0.40528 |
| C | -1.54927 | -1.31919 | 1.489245 | C | -1.5448 | -1.34494 | 1.469792 |
| C | -0.28063 | -1.50021 | 0.666728 | C | -0.27607 | -1.50503 | 0.643281 |
| C | -0.33143 | -2.05601 | -0.61892 | C | -0.32645 | -2.01991 | -0.65912 |
| C | 0.833025 | -2.24976 | -1.35234 | C | 0.83888 | -2.19372 | -1.39651 |
| C | 2.076696 | -1.90347 | -0.8132 | C | 2.082568 | -1.86816 | -0.84456 |
| C | 2.16021 | -1.36281 | 0.483432 | C | 2.165632 | -1.37098 | 0.469457 |
| C | 0.969329 | -1.16523 | 1.191467 | C | 0.974184 | -1.19217 | 1.181081 |
| O | -4.56549 | 1.65565 | -1.06533 | O | -4.58646 | 1.666306 | -1.01332 |
| O | -3.73731 | -2.50021 | -0.14797 | O | -3.74634 | -2.49909 | -0.16661 |
| C | -4.9011 | -3.30354 | -0.33679 | C | -4.91109 | -3.30129 | -0.35402 |
| O | 3.180633 | -2.13503 | -1.58509 | O | 3.187403 | -2.07556 | -1.62208 |
| C | 3.50567 | -1.0386 | 1.112564 | C | 3.510861 | -1.07166 | 1.111296 |
| C | 4.354992 | -0.09711 | 0.286094 | C | 4.363423 | -0.10374 | 0.319678 |
| C | 5.655664 | -0.21378 | -0.04235 | C | 5.662315 | -0.21599 | -0.0172 |
| C | 6.338215 | 0.854831 | -0.86305 | C | 6.35048 | 0.881471 | -0.79404 |
| C | 6.554656 | -1.35311 | 0.373495 | C | 6.55494 | -1.37723 | 0.349177 |
| C | -1.77003 | 1.733749 | 0.157256 | C | -1.77267 | 1.725263 | 0.172514 |
| C | -1.73902 | 2.616207 | -0.94132 | C | -0.82059 | 1.917084 | 1.191205 |
| C | -0.82505 | 3.659682 | -1.00217 | C | 0.09624 | 2.958432 | 1.144611 |
| C | 0.078795 | 3.860525 | 0.046292 | C | 0.086662 | 3.843607 | 0.060993 |
| C | 0.05434 | 3.006779 | 1.154189 | C | -0.85581 | 3.678188 | -0.95851 |
| C | -0.85834 | 1.957975 | 1.201441 | C | -1.77624 | 2.636917 | -0.89877 |
| O | 0.95331 | 4.902797 | -0.06279 | O | 1.012482 | 4.846492 | 0.057349 |
| H | -4.51835 | -1.52807 | 1.483226 | H | -4.50978 | -1.55404 | 1.489155 |
| H | -1.30288 | -0.80704 | 2.426729 | H | -1.2994 | -0.85194 | 2.417935 |
| H | -1.93034 | -2.30587 | 1.778711 | H | -1.92285 | -2.33859 | 1.738821 |
| H | -1.29192 | -2.32867 | -1.04615 | H | -1.28695 | -2.27643 | -1.09631 |
| H | 0.804369 | -2.6673 | -2.35357 | H | 0.81075 | -2.57899 | -2.41063 |
| H | 1.030314 | -0.74511 | 2.193796 | H | 1.034685 | -0.80406 | 2.196235 |
| H | -4.56743 | -4.22749 | -0.81137 | H | -4.58193 | -4.21651 | -0.84829 |
| H | -5.37652 | -3.54121 | 0.625476 | H | -5.37387 | -3.55613 | 0.610005 |
| H | -5.62852 | -2.80008 | -0.9818 | H | -5.64759 | -2.78853 | -0.98121 |
| H | 3.91622 | -1.59601 | -1.24903 | H | 3.92262 | -1.54828 | -1.26668 |
| H | 3.319931 | -0.58053 | 2.093145 | H | 3.324914 | -0.6465 | 2.106392 |
| H | 4.04961 | -1.96922 | 1.314089 | H | 4.052181 | -2.01003 | 1.281344 |
| H | 3.824907 | 0.787614 | -0.06772 | H | 3.83882 | 0.799026 | 0.005913 |
| H | 5.651124 | 1.657235 | -1.1432 | H | 5.66847 | 1.700118 | -1.03704 |
| H | 7.173886 | 1.298567 | -0.30642 | H | 7.190388 | 1.295152 | -0.22098 |
| H | 6.766744 | 0.434041 | -1.78154 | H | 6.773868 | 0.496951 | -1.73073 |
| H | 6.033147 | -2.14711 | 0.910302 | H | 6.029424 | -2.19007 | 0.85289 |
| H | 7.044976 | -1.7989 | -0.5007 | H | 7.041852 | -1.78879 | -0.54356 |
| H | 7.357968 | -0.98278 | 1.023267 | H | 7.360935 | -1.03928 | 1.013087 |
| H | -2.44772 | 2.482994 | -1.74856 | H | -0.80992 | 1.255394 | 2.049072 |
| H | -0.80032 | 4.332653 | -1.85252 | H | 0.820927 | 3.105915 | 1.938219 |
| H | 0.743763 | 3.165997 | 1.980784 | H | -0.87274 | 4.367163 | -1.80042 |
| H | -0.87417 | 1.32199 | 2.078772 | H | -2.51295 | 2.532477 | -1.68466 |
| H | 1.502708 | 4.939634 | 0.731279 | H | 0.886279 | 5.388593 | -0.73273 |
| (4*R*)-**2**-k | | | | (4*R*)-**2**-l | | | |
| C | 2.524053 | 0.595236 | 0.47511 | C | -2.48101 | -0.53236 | 0.278491 |
| C | 1.513982 | 1.176246 | -0.20464 | C | -1.46605 | -1.22525 | -0.27837 |
| C | 1.048218 | 2.393988 | 0.558854 | C | -1.38752 | -2.58608 | 0.372804 |
| O | 1.851929 | 2.429732 | 1.747959 | O | -2.41181 | -2.5867 | 1.379433 |
| C | 2.736253 | 1.379767 | 1.733896 | C | -3.07815 | -1.38737 | 1.354402 |
| C | 0.850891 | 0.801233 | -1.49996 | C | -0.49113 | -0.84995 | -1.35875 |
| C | -0.66738 | 0.768707 | -1.40853 | C | 0.95538 | -1.13635 | -0.98407 |
| C | -1.44896 | 1.837897 | -1.86039 | C | 1.619438 | -2.27565 | -1.45319 |
| C | -2.83625 | 1.803323 | -1.74982 | C | 2.938745 | -2.53134 | -1.09055 |
| C | -3.4718 | 0.690647 | -1.19141 | C | 3.618383 | -1.64619 | -0.25049 |
| C | -2.71227 | -0.40853 | -0.74032 | C | 2.978442 | -0.49385 | 0.242554 |
| C | -1.32092 | -0.33645 | -0.85151 | C | 1.653249 | -0.26634 | -0.13933 |
| O | 3.507954 | 1.181831 | 2.63882 | O | -3.9709 | -1.13609 | 2.125237 |
| O | 1.235596 | 3.529979 | -0.22584 | O | -1.60756 | -3.57004 | -0.58837 |
| C | 0.733613 | 4.726446 | 0.367535 | C | -1.45658 | -4.89963 | -0.09318 |
| O | -4.83554 | 0.713433 | -1.1314 | O | 4.917533 | -1.95876 | 0.052128 |
| C | -3.38836 | -1.65788 | -0.19847 | C | 3.715352 | 0.489627 | 1.137447 |
| C | -4.3524 | -1.39252 | 0.937835 | C | 4.684265 | 1.364832 | 0.370806 |
| C | -5.60348 | -1.86417 | 1.098545 | C | 4.806334 | 2.699843 | 0.413489 |
| C | -6.40246 | -1.51493 | 2.331599 | C | 5.833237 | 3.403542 | -0.44138 |
| C | -6.31221 | -2.78329 | 0.133212 | C | 3.980205 | 3.619777 | 1.277565 |
| C | 3.347694 | -0.57888 | 0.149111 | C | -3.00812 | 0.805997 | -0.0276 |
| C | 3.760617 | -1.46754 | 1.161411 | C | -3.06972 | 1.288492 | -1.34866 |
| C | 4.51963 | -2.59174 | 0.866323 | C | -3.55279 | 2.559308 | -1.63554 |
| C | 4.900326 | -2.852 | -0.45537 | C | -3.99799 | 3.383005 | -0.59604 |
| C | 4.521964 | -1.96972 | -1.47231 | C | -3.9659 | 2.914685 | 0.722152 |
| C | 3.757662 | -0.84617 | -1.16728 | C | -3.48295 | 1.640075 | 0.999674 |
| O | 5.64601 | -3.97102 | -0.68738 | O | -4.46111 | 4.622542 | -0.92894 |
| H | 0.002011 | 2.320536 | 0.889922 | H | -0.43535 | -2.76096 | 0.894554 |
| H | 1.147258 | 1.532718 | -2.26293 | H | -0.74459 | -1.4144 | -2.26542 |
| H | 1.227362 | -0.17202 | -1.82794 | H | -0.61215 | 0.210105 | -1.59924 |
| H | -0.96636 | 2.70992 | -2.29294 | H | 1.098444 | -2.97097 | -2.1054 |
| H | -3.45027 | 2.629742 | -2.09262 | H | 3.46308 | -3.41012 | -1.45126 |
| H | -0.72907 | -1.18014 | -0.50136 | H | 1.152066 | 0.624187 | 0.234534 |
| H | 0.872657 | 5.520502 | -0.36804 | H | -1.57907 | -5.56589 | -0.94872 |
| H | -0.33611 | 4.632485 | 0.601563 | H | -0.45836 | -5.04801 | 0.342075 |
| H | 1.281898 | 4.974895 | 1.282261 | H | -2.2161 | -5.12958 | 0.661348 |
| H | -5.13379 | 0.041925 | -0.49429 | H | 5.268922 | -1.31526 | 0.680095 |
| H | -3.89661 | -2.18041 | -1.01772 | H | 2.981996 | 1.095046 | 1.674844 |
| H | -2.60488 | -2.34133 | 0.154138 | H | 4.256901 | -0.06298 | 1.925257 |
| H | -3.95202 | -0.75354 | 1.725372 | H | 5.346596 | 0.825762 | -0.3073 |
| H | -5.85244 | -0.84937 | 3.001621 | H | 6.415914 | 2.701521 | -1.04361 |
| H | -7.34941 | -1.02889 | 2.064512 | H | 5.353085 | 4.118883 | -1.12185 |
| H | -6.66638 | -2.42105 | 2.892058 | H | 6.530529 | 3.983768 | 0.177111 |
| H | -5.75638 | -2.96217 | -0.78866 | H | 3.215627 | 3.104074 | 1.861137 |
| H | -6.49614 | -3.75679 | 0.605574 | H | 4.622459 | 4.165959 | 1.980561 |
| H | -7.29597 | -2.37916 | -0.13522 | H | 3.480233 | 4.378523 | 0.662258 |
| H | 3.488556 | -1.26156 | 2.189562 | H | -2.76597 | 0.646396 | -2.16869 |
| H | 4.832838 | -3.27743 | 1.646344 | H | -3.60724 | 2.923758 | -2.65573 |
| H | 4.835928 | -2.15208 | -2.49784 | H | -4.32464 | 3.546478 | 1.532057 |
| H | 3.505231 | -0.15176 | -1.96174 | H | -3.48308 | 1.278257 | 2.020642 |
| H | 5.853922 | -4.02472 | -1.62955 | H | -4.75928 | 5.074838 | -0.12865 |
| (4*R*)-**2**-m | | | | (4*R*)-**2**-n | | | |
| C | -2.47896 | -0.52224 | 0.283194 | C | -2.16026 | 1.262243 | -0.18749 |
| C | -1.47628 | -1.22836 | -0.27863 | C | -1.11979 | 1.268959 | -1.05186 |
| C | -1.41037 | -2.58895 | 0.374498 | C | -0.52102 | 2.655839 | -1.07996 |
| O | -2.42999 | -2.57618 | 1.385459 | O | -1.28436 | 3.426777 | -0.14562 |
| C | -3.08191 | -1.36829 | 1.362751 | C | -2.26313 | 2.637294 | 0.405166 |
| C | -0.50353 | -0.86542 | -1.36482 | C | -0.61174 | 0.205558 | -1.9861 |
| C | 0.94305 | -1.15279 | -0.99108 | C | 0.373244 | -0.8178 | -1.42068 |
| C | 1.607337 | -2.2905 | -1.46345 | C | 0.435724 | -2.09295 | -1.99945 |
| C | 2.926948 | -2.54655 | -1.10199 | C | 1.339214 | -3.04528 | -1.54059 |
| C | 3.606414 | -1.66338 | -0.25974 | C | 2.201712 | -2.73777 | -0.48562 |
| C | 2.966002 | -0.51277 | 0.236928 | C | 2.161687 | -1.46979 | 0.119331 |
| C | 1.640652 | -0.28484 | -0.14389 | C | 1.245248 | -0.53032 | -0.36608 |
| O | -3.96828 | -1.10501 | 2.136369 | O | -3.04374 | 3.073714 | 1.213861 |
| O | -1.64555 | -3.57173 | -0.58451 | O | 0.826372 | 2.626349 | -0.73296 |
| C | -1.50953 | -4.90224 | -0.08747 | C | 1.481411 | 3.889445 | -0.8383 |
| O | 4.905856 | -1.97593 | 0.041374 | O | 3.065616 | -3.72475 | -0.08725 |
| C | 3.702565 | 0.468252 | 1.134762 | C | 3.105075 | -1.11595 | 1.258525 |
| C | 4.670674 | 1.346612 | 0.370682 | C | 4.520837 | -0.85669 | 0.788778 |
| C | 4.793718 | 2.681316 | 0.420071 | C | 5.305594 | 0.195748 | 1.063546 |
| C | 5.820179 | 3.38854 | -0.43244 | C | 6.700657 | 0.28247 | 0.491706 |
| C | 3.969746 | 3.597328 | 1.290334 | C | 4.916704 | 1.362405 | 1.937133 |
| C | -2.98913 | 0.823178 | -0.02117 | C | -3.10345 | 0.193301 | 0.169805 |
| C | -3.42643 | 1.675829 | 1.011581 | C | -2.73862 | -1.16645 | 0.110294 |
| C | -3.88953 | 2.955982 | 0.739803 | C | -3.64425 | -2.17326 | 0.418597 |
| C | -3.94139 | 3.415684 | -0.58146 | C | -4.94959 | -1.84381 | 0.798944 |
| C | -3.53602 | 2.576351 | -1.62405 | C | -5.32925 | -0.49932 | 0.876778 |
| C | -3.06942 | 1.295073 | -1.34157 | C | -4.41643 | 0.503989 | 0.571639 |
| O | -4.40456 | 4.682121 | -0.79016 | O | -5.80077 | -2.86942 | 1.088243 |
| H | -0.45784 | -2.77384 | 0.892214 | H | -0.64681 | 3.139352 | -2.06409 |
| H | -0.76086 | -1.43709 | -2.26581 | H | -1.47518 | -0.34028 | -2.38573 |
| H | -0.6226 | 0.193057 | -1.61359 | H | -0.14123 | 0.707874 | -2.84297 |
| H | 1.086547 | -2.9844 | -2.1174 | H | -0.23455 | -2.34803 | -2.81678 |
| H | 3.45149 | -3.42424 | -1.46502 | H | 1.386838 | -4.03603 | -1.98037 |
| H | 1.138892 | 0.604008 | 0.233145 | H | 1.220072 | 0.45393 | 0.090343 |
| H | -1.64225 | -5.56844 | -0.94155 | H | 2.535941 | 3.710245 | -0.62358 |
| H | -0.51211 | -5.06213 | 0.345587 | H | 1.383559 | 4.300608 | -1.85286 |
| H | -2.26981 | -5.12167 | 0.669399 | H | 1.074752 | 4.60732 | -0.11861 |
| H | 5.256642 | -1.33481 | 0.672132 | H | 3.549782 | -3.43365 | 0.695526 |
| H | 2.969044 | 1.071391 | 1.674474 | H | 2.703365 | -0.25088 | 1.790261 |
| H | 4.244708 | -0.08659 | 1.920589 | H | 3.106717 | -1.93087 | 2.00384 |
| H | 5.331749 | 0.81043 | -0.31093 | H | 4.931708 | -1.62603 | 0.134126 |
| H | 6.401242 | 2.689186 | -1.03933 | H | 6.942451 | -0.58233 | -0.13181 |
| H | 5.339899 | 4.108276 | -1.10821 | H | 6.81972 | 1.186626 | -0.11981 |
| H | 6.518985 | 3.964567 | 0.188242 | H | 7.451487 | 0.347753 | 1.290229 |
| H | 3.204262 | 3.079588 | 1.87087 | H | 3.891746 | 1.308991 | 2.30756 |
| H | 4.613352 | 4.137588 | 1.996648 | H | 5.582781 | 1.431471 | 2.807078 |
| H | 3.471109 | 4.361179 | 0.6803 | H | 5.027921 | 2.306819 | 1.388763 |
| H | -3.41015 | 1.319204 | 2.034409 | H | -1.72349 | -1.44154 | -0.15011 |
| H | -4.2213 | 3.613197 | 1.536457 | H | -3.35459 | -3.21792 | 0.380576 |
| H | -3.59814 | 2.917784 | -2.65514 | H | -6.34049 | -0.23655 | 1.180459 |
| H | -2.79524 | 0.643114 | -2.16427 | H | -4.71737 | 1.540503 | 0.654117 |
| H | -4.40629 | 4.867594 | -1.73854 | H | -6.65753 | -2.50596 | 1.348899 |
| (4*R*)-**2**-o | | | | (4*R*)-**2**-p | | | |
| C | -2.16211 | 1.268041 | -0.18374 | C | 2.657528 | 0.542636 | 0.37029 |
| C | -1.12188 | 1.276005 | -1.04802 | C | 1.61036 | 1.127104 | -0.24792 |
| C | -0.52254 | 2.662712 | -1.0734 | C | 1.316741 | 2.445325 | 0.429233 |
| O | -1.28533 | 3.431907 | -0.13768 | O | 2.258349 | 2.537501 | 1.509718 |
| C | -2.26469 | 2.641548 | 0.412258 | C | 3.066021 | 1.428188 | 1.508002 |
| C | -0.61592 | 0.212828 | -1.98362 | C | 0.775201 | 0.674592 | -1.41266 |
| C | 0.367586 | -0.81278 | -1.41984 | C | -0.72068 | 0.794593 | -1.16274 |
| C | 0.424848 | -2.08895 | -1.99692 | C | -1.45868 | 1.873735 | -1.6616 |
| C | 1.326256 | -3.04364 | -1.53863 | C | -2.82722 | 1.970814 | -1.42605 |
| C | 2.191955 | -2.73752 | -0.48583 | C | -3.48421 | 0.992964 | -0.67385 |
| C | 2.157525 | -1.46844 | 0.117196 | C | -2.76395 | -0.0953 | -0.13979 |
| C | 1.243161 | -0.52664 | -0.36774 | C | -1.3938 | -0.17117 | -0.40578 |
| O | -3.04439 | 3.075424 | 1.222589 | O | 3.924493 | 1.261417 | 2.338478 |
| O | 0.82512 | 2.631407 | -0.72634 | O | 1.479314 | 3.481712 | -0.48735 |
| C | 1.480273 | 3.894898 | -0.82676 | C | 1.124041 | 4.763966 | 0.027766 |
| O | 3.052511 | -3.72735 | -0.08716 | O | -4.8249 | 1.154789 | -0.4707 |
| C | 3.10464 | -1.11602 | 1.253732 | C | -3.44954 | -1.13284 | 0.735152 |
| C | 4.519915 | -0.86063 | 0.780277 | C | -4.6487 | -1.78743 | 0.083978 |
| C | 5.308148 | 0.189719 | 1.053141 | C | -5.87673 | -1.98459 | 0.600354 |
| C | 6.701941 | 0.272782 | 0.477709 | C | -6.94207 | -2.69293 | -0.20227 |
| C | 4.924565 | 1.357327 | 1.927764 | C | -6.30926 | -1.57238 | 1.986804 |
| C | -3.10466 | 0.19806 | 0.172024 | C | 3.364306 | -0.71298 | 0.07623 |
| C | -4.42632 | 0.505165 | 0.558242 | C | 3.585576 | -1.14142 | -1.24627 |
| C | -5.3385 | -0.49555 | 0.861846 | C | 4.23985 | -2.33586 | -1.52252 |
| C | -4.95062 | -1.83905 | 0.799649 | C | 4.69961 | -3.13429 | -0.46981 |
| C | -3.64005 | -2.16487 | 0.436089 | C | 4.508643 | -2.71659 | 0.851947 |
| C | -2.73184 | -1.15679 | 0.12697 | C | 3.855333 | -1.51797 | 1.118924 |
| O | -5.88682 | -2.7809 | 1.110365 | O | 5.333844 | -4.29865 | -0.79236 |
| H | -0.64756 | 3.148215 | -2.05664 | H | 0.315515 | 2.482957 | 0.882556 |
| H | -1.48084 | -0.33135 | -2.38246 | H | 1.04103 | 1.284515 | -2.28585 |
| H | -0.14531 | 0.714744 | -2.84065 | H | 1.032732 | -0.35983 | -1.65786 |
| H | -0.24735 | -2.3425 | -2.8132 | H | -0.95797 | 2.641298 | -2.2451 |
| H | 1.370778 | -4.03478 | -1.97797 | H | -3.41114 | 2.794488 | -1.82363 |
| H | 1.222123 | 0.458508 | 0.087014 | H | -0.83302 | -1.01198 | -0.00173 |
| H | 2.534774 | 3.71491 | -0.61249 | H | 1.224525 | 5.469731 | -0.79848 |
| H | 1.382641 | 4.309896 | -1.83977 | H | 0.085294 | 4.771223 | 0.386576 |
| H | 1.073399 | 4.610027 | -0.10451 | H | 1.789812 | 5.060376 | 0.845088 |
| H | 3.546171 | -3.43316 | 0.688491 | H | -5.20815 | 0.300541 | -0.20825 |
| H | 2.706505 | -0.24942 | 1.785627 | H | -2.71271 | -1.90827 | 0.981875 |
| H | 3.105925 | -1.93039 | 1.999647 | H | -3.7342 | -0.67373 | 1.689434 |
| H | 4.927216 | -1.63085 | 0.124371 | H | -4.46456 | -2.15328 | -0.92647 |
| H | 6.940147 | -0.59288 | -0.14599 | H | -6.58695 | -2.97515 | -1.19656 |
| H | 6.821505 | 1.176358 | -0.13452 | H | -7.2745 | -3.60355 | 0.312574 |
| H | 7.454889 | 0.336721 | 1.274313 | H | -7.8317 | -2.06156 | -0.32126 |
| H | 3.900197 | 1.307138 | 2.300273 | H | -5.55484 | -0.99932 | 2.52818 |
| H | 5.592599 | 1.424075 | 2.796372 | H | -7.22635 | -0.97209 | 1.943778 |
| H | 5.037691 | 2.301471 | 1.379377 | H | -6.54808 | -2.45955 | 2.587138 |
| H | -4.73137 | 1.541536 | 0.628097 | H | 3.270061 | -0.51381 | -2.07306 |
| H | -6.35552 | -0.25622 | 1.153547 | H | 4.416613 | -2.6579 | -2.54312 |
| H | -3.32508 | -3.2058 | 0.404097 | H | 4.877178 | -3.32753 | 1.673396 |
| H | -1.71332 | -1.43052 | -0.12137 | H | 3.732571 | -1.19241 | 2.144699 |
| H | -5.4892 | -3.65893 | 1.039778 | H | 5.619806 | -4.7384 | 0.01928 |
| (4*R*)-**2**-q | | | | (4*R*)-**2**-r | | | |
| C | 2.65423 | 0.540113 | 0.376854 | C | -1.86022 | -1.15197 | 0.326085 |
| C | 1.614353 | 1.131374 | -0.24661 | C | -0.7142 | -1.60951 | -0.2186 |
| C | 1.324843 | 2.45074 | 0.430171 | C | -0.19179 | -2.74783 | 0.626489 |
| O | 2.262114 | 2.537112 | 1.514583 | O | -1.11762 | -2.8692 | 1.717456 |
| C | 3.063426 | 1.422566 | 1.516574 | C | -2.11577 | -1.93747 | 1.576011 |
| C | 0.783428 | 0.683919 | -1.41604 | C | 0.043248 | -1.17047 | -1.43927 |
| C | -0.71323 | 0.801534 | -1.16959 | C | 1.517886 | -0.91255 | -1.16601 |
| C | -1.45266 | 1.876716 | -1.67472 | C | 2.50207 | -1.83244 | -1.54186 |
| C | -2.82182 | 1.971824 | -1.44209 | C | 3.846844 | -1.57945 | -1.28484 |
| C | -3.47796 | 0.99617 | -0.68623 | C | 4.226979 | -0.40006 | -0.64118 |
| C | -2.75622 | -0.08798 | -0.14572 | C | 3.258728 | 0.538671 | -0.23322 |
| C | -1.38547 | -0.16209 | -0.4091 | C | 1.918772 | 0.259105 | -0.51286 |
| O | 3.917883 | 1.250495 | 2.349584 | O | -3.0028 | -1.8225 | 2.384474 |
| O | 1.496813 | 3.486612 | -0.48553 | O | -0.14344 | -3.90816 | -0.14283 |
| C | 1.148294 | 4.770785 | 0.029382 | C | 0.431302 | -5.02352 | 0.536229 |
| O | -4.81919 | 1.15636 | -0.48621 | O | 5.568469 | -0.2153 | -0.43852 |
| C | -3.44045 | -1.12254 | 0.733906 | C | 3.688413 | 1.824254 | 0.465817 |
| C | -4.63976 | -1.78069 | 0.086715 | C | 2.596344 | 2.526477 | 1.229019 |
| C | -5.86674 | -1.97769 | 0.605629 | C | 2.080806 | 3.745628 | 1.008138 |
| C | -6.93245 | -2.69001 | -0.19294 | C | 0.999205 | 4.294421 | 1.907803 |
| C | -6.29756 | -1.5611 | 1.991269 | C | 2.497455 | 4.674316 | -0.10529 |
| C | 3.353703 | -0.72036 | 0.08558 | C | -2.78219 | -0.09875 | -0.12571 |
| C | 3.816934 | -1.54231 | 1.131812 | C | -3.40885 | 0.75309 | 0.804998 |
| C | 4.460487 | -2.74434 | 0.871043 | C | -4.26497 | 1.764708 | 0.391614 |
| C | 4.672244 | -3.15244 | -0.45121 | C | -4.53019 | 1.943676 | -0.97119 |
| C | 4.242462 | -2.33974 | -1.50498 | C | -3.93826 | 1.093101 | -1.91024 |
| C | 3.594159 | -1.13734 | -1.23373 | C | -3.07782 | 0.083373 | -1.4864 |
| O | 5.310154 | -4.34255 | -0.64839 | O | -5.37889 | 2.953742 | -1.32038 |
| H | 0.321741 | 2.493554 | 0.878938 | H | 0.79148 | -2.53778 | 1.072276 |
| H | 1.051007 | 1.29857 | -2.28533 | H | -0.04761 | -1.95388 | -2.20255 |
| H | 1.043195 | -0.34915 | -1.66511 | H | -0.42384 | -0.26986 | -1.84823 |
| H | -0.9526 | 2.642849 | -2.2607 | H | 2.216002 | -2.75352 | -2.04219 |
| H | -3.40684 | 2.792493 | -1.84421 | H | 4.620151 | -2.2803 | -1.5817 |
| H | -0.8234 | -0.99938 | 0.00044 | H | 1.168656 | 0.984943 | -0.21049 |
| H | 1.255297 | 5.476412 | -0.79618 | H | 0.476416 | -5.83888 | -0.1877 |
| H | 0.108705 | 4.784439 | 0.385596 | H | 1.447379 | -4.79273 | 0.885886 |
| H | 1.813725 | 5.062517 | 0.848644 | H | -0.18302 | -5.32644 | 1.390604 |
| H | -5.20082 | 0.303561 | -0.21667 | H | 5.717881 | 0.637398 | -0.01171 |
| H | -2.70282 | -1.89636 | 0.983233 | H | 4.483104 | 1.580062 | 1.191362 |
| H | -3.72437 | -0.65964 | 1.686581 | H | 4.149537 | 2.502266 | -0.26371 |
| H | -4.45664 | -2.14999 | -0.9227 | H | 2.194738 | 1.95371 | 2.064755 |
| H | -6.57844 | -2.97544 | -1.18671 | H | 0.732407 | 3.592103 | 2.701679 |
| H | -7.26322 | -3.59908 | 0.325675 | H | 1.317759 | 5.234655 | 2.376657 |
| H | -7.82288 | -2.05995 | -0.31294 | H | 0.091029 | 4.524613 | 1.336004 |
| H | -5.54264 | -0.98591 | 2.529629 | H | 3.24372 | 4.245271 | -0.77637 |
| H | -7.21502 | -0.96143 | 1.947561 | H | 1.627628 | 4.957795 | -0.71119 |
| H | -6.53504 | -2.44644 | 2.594811 | H | 2.907163 | 5.607384 | 0.302723 |
| H | 3.676775 | -1.22259 | 2.15734 | H | -3.2261 | 0.606626 | 1.862726 |
| H | 4.812478 | -3.37853 | 1.677619 | H | -4.74343 | 2.422598 | 1.109244 |
| H | 4.426397 | -2.63858 | -2.53473 | H | -4.16025 | 1.210024 | -2.96884 |
| H | 3.301399 | -0.50047 | -2.06181 | H | -2.65742 | -0.59211 | -2.224 |
| H | 5.406307 | -4.4984 | -1.59721 | H | -5.49185 | 2.95646 | -2.28008 |
